# Pore Engineering with Benzoate Derivatives in Supramolecular Copper–Adenine Systems for Water and Drug Sorption

**DOI:** 10.1021/acs.inorgchem.6c04013

**Published:** 2026-09-23

**Authors:** Sandra Mena-Gutiérrez, Jon Pascual-Colino, Ainara Castellanos-Rubio, Jon B. Etxebarria, Antonio Luque

**Affiliations:** † Departamento de Química Orgánica e Inorgánica, Universidad del País Vasco/Euskal Herriko Unibertsitatea, EHU, E-48940 Leioa, Spain; ‡ Dipartimento di Chimica, UNIMI, Università degli studi di Milano, Via golgi 19, Milano 21033, Italy; § IKERBASQUE, Basque Foundation for Science, E-48011 Bilbao, Spain; ∥ Biobizkaia Research Institute, E-48903 Barakaldo, Spain; ⊥ Departamento de Genética, Antropología Física y Fisiología Animal, EHU, E-48940 Leio, Spain; # BC Materials, Basque Center for Materials, Applications and Nanostructures, EHU Science Park, E-48940 Leioa, Spain

## Abstract

Porous supramolecular metal–organic frameworks (SMOFs) are versatile functional materials for controlled drug delivery and tailored water vapor sorption whose properties are deeply dependent on their specific internal microenvironment. Precisely tuning the internal pore environment of a coordination framework without perturbing its underlying topology remains a fundamental challenge in crystal engineering. Herein, we address this challenge in a family of water-stable copper–adenine supramolecular metal–organic frameworks (SMOFs). By systematically varying the monocarboxylate counterion, fine control over the accessible void space (ranging from 12 to 29%) was achieved, which dictates their water vapor sorption behavior through the simultaneous modification of steric hindrance and hydrophobicity. Furthermore, this work introduces a nondestructive magnetic sustentation technique that leverages the room-temperature paramagnetic nature of these frameworks to quantify the loading of highly insoluble drugs (5-fluorouracil, cycloserine, and gabapentin). In this context, drug uptake and release act as complementary experimental probes to validate our structural design. The structurally optimized **Cu**_**7**_ **Bz** material achieved a notable 31.4% loading of 5-fluorouracil. Finally, in vitro assays on *HCT116* colorectal cancer cells reveal that the compact pore architecture of **5-FU@Cu**_**7**_ **Bz** effectively mitigates the toxic “*burst-release*” effect, providing a sustained and potent antiproliferative response. These results establish counterion-directed pore engineering as a practical strategy for the design of advanced solid-state materials and introduce magnetic sustentation as a robust tool for evaluating complex host–guest systems.

## Graphical abstract

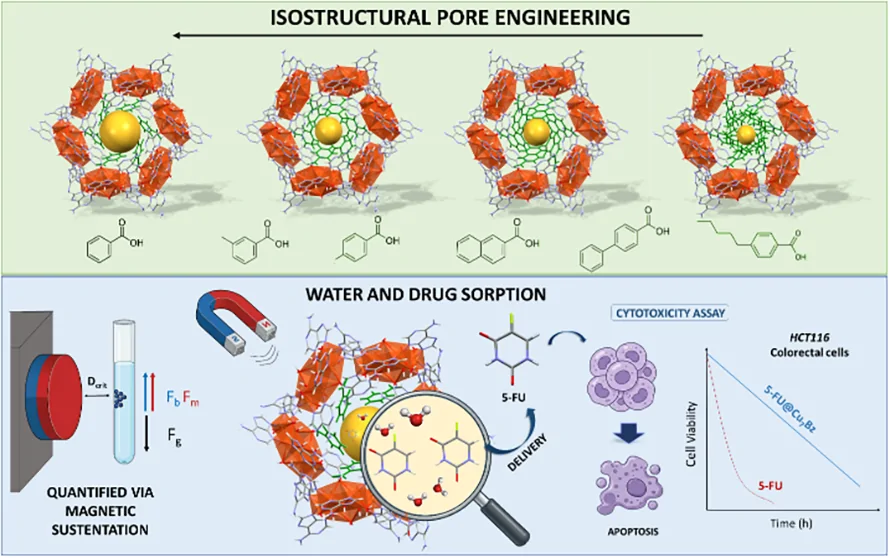

## 1 Introduction

Precise control over the pore dimensions and chemical environment of a framework, without perturbing its underlying connectivity, remains one of the central challenges in crystal engineering and coordination chemistry.^1,2^ Meeting this challenge has direct implications for understanding and mastering complex host-guest interactions, such as controlling water vapor affinity and the selective encapsulation of poorly soluble therapeutics. Both processes demand the development of advanced materials capable of precise molecular recognition and controlled mass transport. Modulating water sorption behavior in porous solids requires sorbents with finely tuned hydrophilicity and optimal pore thermodynamics to dictate their water affinity at varying humidity levels.^3,4^ In parallel, a major limitation in modern biomedicine is the inadequate aqueous solubility of many potent active pharmaceutical ingredients (APIs), including essential antitumoral and antibiotic agents. This physical constraint drives the need for the development of intelligent nanocarriers that can facilitate the encapsulation, protection, and sustained release of these APIs.^5,6^ Meeting these disparate yet fundamentally related demands requires a deep level of control over the internal environment of solid-state carriers. Consequently, crystal design has emerged as a powerful strategy for the rational construction of functional solid-state materials, enabling the deliberate control of structure–property relationships at the molecular and supramolecular levels.^7–10^ Through the careful selection of molecular building blocks and non-covalent or coordination interactions, crystal design has enabled the creation of materials with tailored properties for a range of applications,^11^ including gas storage and separation,^12^ catalysis,^13^ sensing^14^ and biomedicine.^15^ In this context, porosity plays a central role; the size, shape, and chemical environment of pores significantly influence mass transport phenomena and host–guest interactions.^16^

Metal–organic frameworks (MOFs) and related metal–organic porous solids are among the most influential porous materials due to their structural modularity and synthetic versatility.^17,18^ The recent recognition of this field with the 2025 Nobel Prize in Chemistry (awarded to Susumu Kitagawa, Richard Robson and Omar M. Yaghi) highlights the field’s broad impact on the development of materials science.^19^ A fundamental milestone in this area is the isoreticular approach, where a fixed network topology is preserved while the length of the organic linker is systematically varied to expand the pore dimensions predictably.^20,21^ This strategy provides robust control over pore dimensions and surface area, and has contributed to the recognition of reticular chemistry as a cornerstone of modern materials science. One example of these well-known materials is the IRMOF (isoreticular metal–organic framework) series, in which a common cubic metal center SBU topology is preserved, while the length and functionality of the organic linkers vary systematically. Although this strategy provides robust control over pore size and surface area,^22,23^ modifying the intrinsic chemical environment of the pore without altering the underlying framework topology remains a significant challenge.

The principles of crystal design and isoreticularity have been extended beyond coordination polymers and extended frameworks to porous supramolecular metal–organic systems (SMOFs).^24–30^ These materials are synthesized through milder processes, and their highly adaptable structures can undergo reversible assembly/disassembly. Their supramolecular architecture is governed by a combination of multiple weaker interactions, such as hydrogen bonding and π-stacking, rather than the covalent interactions used in MOFs. Recently, our research group demonstrated how to modulate pore size in copper-adenine supramolecular structures by intercalating progressively longer dicarboxylate counterions (from fumarate to biphenyl-4,4’-dicarboxylate) between π-stacked adeninato ligands, creating a *sandwich effect* (Figure 1).^31^ Expanding upon this, we now present a fundamentally distinct design principle. Herein, we report a family of robust, flexible and water-stable copper–adenine supramolecular materials derived from a previously reported monocarboxylate based structure.^32^ The overall crystal architecture of these compounds is preserved while the internal pore environment is systematically engineered. Unlike the conventional isoreticular approach, where pore size increases by linker elongation, without altering the underlying structural motif, our isostructural approach enables the controlled restriction and functionalization of voids space within a fixed structural framework (Figure 2).^33^ By utilizing an isostructural series of copper-adenine frameworks, we can isolate the effect of pore dimensions from other structural variables. In this system, the previously observed sandwich effect is precluded by steric hindrance when two heptameric entities are in close proximity, a configuration particularly evident in the case of monocarboxylate anions.

**Figure 1:**
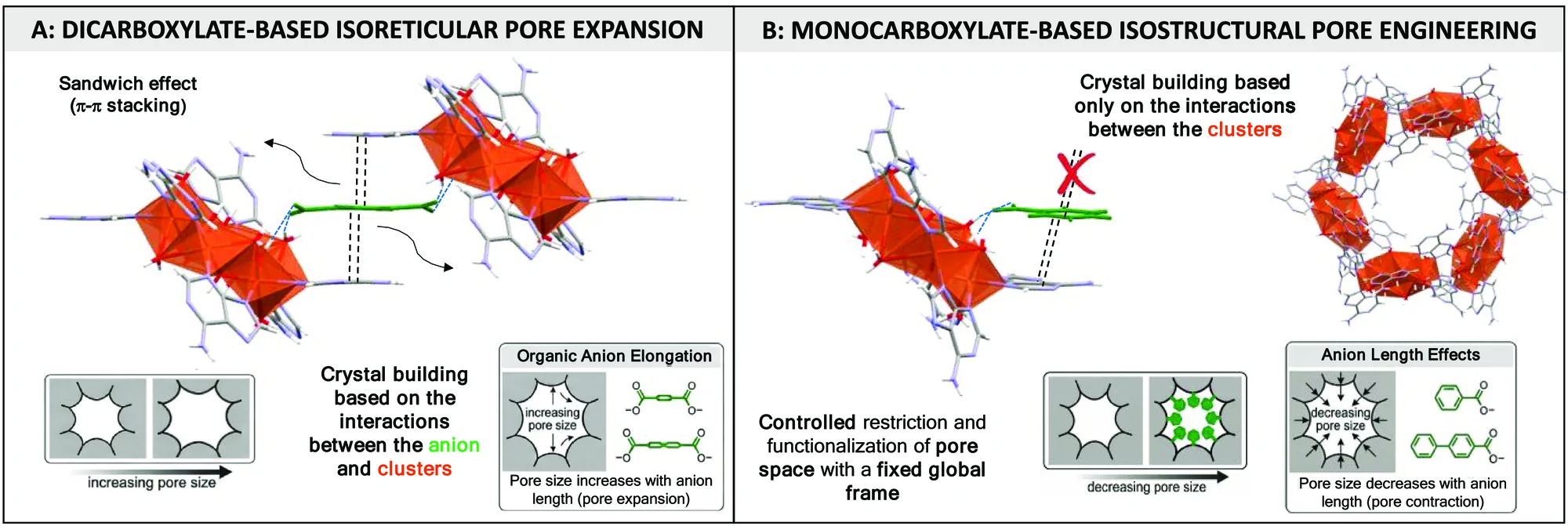
Complementary design principles for pore modulation in supramolecular structures for (a) dicarboxylate and (b) monocarboxylate based isostructural compounds.

In these systems, the anions are not intercalated between the adeninato ligands, instead, they interact with the pore surface through H-bonding and π-stacking interactions. By varying the monocarboxylate counterion, we achieve precise modulation of both the accessible void volume and the chemical nature of the pore surface, without altering the global topology. This strategy provides an additional, largely unexplored level of control over host–guest interactions, allowing the independent tuning of steric confinement and intermolecular interaction strength. As a result, the materials exhibit highly differentiated adsorption behaviors that directly correlate with the nature of the counterion. We validate the broad potential of this concept in two demanding and technologically relevant applications: efficient atmospheric water vapour adsorption and the encapsulation and controlled release of poorly soluble therapeutic agents as complementary experimental probes, as both steric effects and specific intermolecular interactions play a decisive role in their sorption and transport.^34^ Although these materials do not possess the exceptionally large pore sizes characteristic of expanded isoreticular series, their intrinsic structural flexibility facilitates the adsorption of diverse guest molecules. Consequently, the materials can undergo guest-induced expansion, accommodating larger molecules and higher loading capacities than would be predicted from their static crystallographic dimensions. In the context of biomedical applications, a persistent challenge in the design of advanced drug delivery systems concerns the “*burst-release”* effect, whereby a substantial proportion of the therapeutic cargo is released prematurely. Achieving a sustained release kinetics requires a level of pore engineering that goes beyond simple encapsulation, demanding nanometer control over the pore windows to kinetically entrap guest molecules.

**Figure 2:**
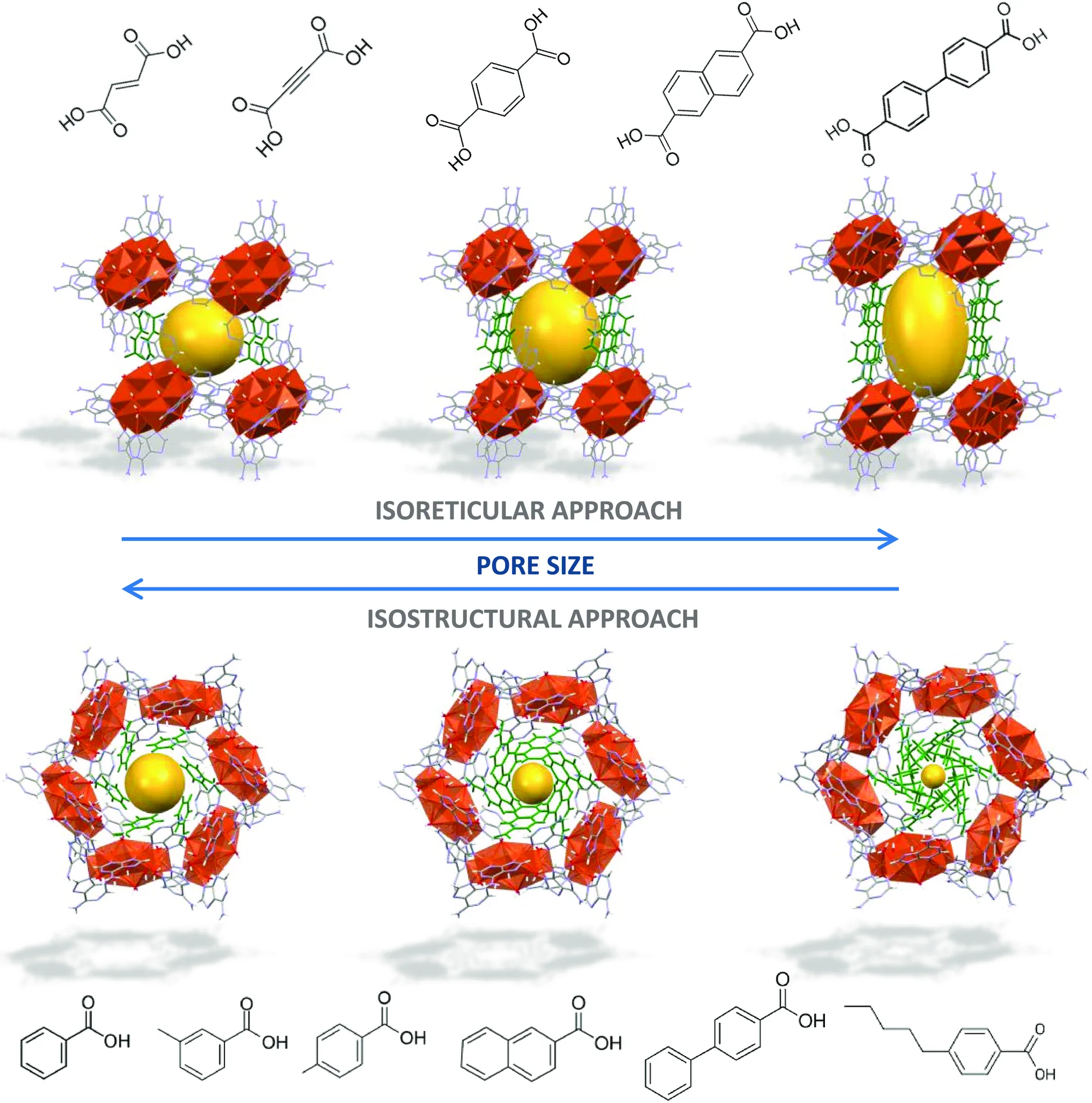
Scheme of isoreticular and isostructural approaches within the family of copper­(II)-adeninato based SMOFs. The voids are highlighted in yellow and the counterions in green.

This study established that counterion engineering in isostructural supramolecular systems provides a versatile strategy for pore modulation and functionalization. It demonstrates how subtle changes in the supramolecular environment directly dictate the macroscopic functional performance of the material in both water adsorption and sustained drug delivery kinetics, offering a coherent structure-property relationship for advanced material design.

## 2 Results and Discussion

### 2.1 Structure Description

This work reports the crystal structures of new compounds for the **Cu**_**7**_ **Bz** system which crystallize in the trigonal *R*3̅ space group with *Z* = 9 (Table 1 and S1), analogous to the previously reported benzoate compound.^32^ Crystallographic data of the compounds are summarized in Table 2 and S2. The synthesis and crystal structure of the **Cu**_**7**_ **Bz** compound were previously published by our research group. For the new compounds, single crystals were selected, removed from the solution, covered with Paratone oil and used for single-crystal X-ray data collection. A distinction is made between pristine compounds measured directly from their mother liquor via SC-XRD (**Cu**_**7**_ **Bz**) and those measured after filtration or drying, which are denoted with the suffix “D” (**Cu**_**7**_ **Bz_D**).

**Table 1:** Formula, Monocarboxylate Anion and Code for the Compounds Reported in This Paper

**formula**	**anion**	**code**
$\left[{\mathrm{Cu}}_{7}{\left(\mu ‐\mathrm{adeninato}\right)}_{6}{\left(\mu_{3}‐\mathrm{OH}\right)}_{6}{\left(\mu ‐{\mathrm{OH}}_{2}\right)}_{6}\right]\cdot {\left(C_{7}H_{5}O_{2}\right)}_{2}\cdot 17H_{2}O$	benzoate	**Cu**_**7**_ **Bz**
$\left[{\mathrm{Cu}}_{7}{\left(\mu ‐\mathrm{adeninato}\right)}_{6}{\left(\mu_{3}‐\mathrm{OH}\right)}_{6}{\left(\mu ‐{\mathrm{OH}}_{2}\right)}_{6}\right]\cdot {\left(C_{7}H_{5}O_{2}\right)}_{2}\cdot 13H_{2}O$	benzoate	**Cu**_**7**_ **Bz_D**
$\left[{\mathrm{Cu}}_{7}{\left(\mu ‐\mathrm{adeninato}\right)}_{6}{\left(\mu_{3}‐\mathrm{OH}\right)}_{6}{\left(\mu ‐{\mathrm{OH}}_{2}\right)}_{6}\right]\cdot {\left(C_{8}H_{7}O_{2}\right)}_{2}\cdot 12H_{2}O$	3-methylbenzoate	**Cu**_**7**_ **3MBz**
$\left[{\mathrm{Cu}}_{7}{\left(\mu ‐\mathrm{adeninato}\right)}_{6}{\left(\mu_{3}‐\mathrm{OH}\right)}_{6}{\left(\mu ‐{\mathrm{OH}}_{2}\right)}_{6}\right]\cdot {\left(C_{8}H_{7}O_{2}\right)}_{2}\cdot 11H_{2}O$	3-methylbenzoate	**Cu**_**7**_ **3MBz_D**
$\left[{\mathrm{Cu}}_{7}{\left(\mu ‐\mathrm{adeninato}\right)}_{6}{\left(\mu_{3}‐\mathrm{OH}\right)}_{6}{\left(\mu ‐{\mathrm{OH}}_{2}\right)}_{6}\right]\cdot {\left(C_{8}H_{7}O_{2}\right)}_{2}\cdot 11H_{2}O$	4-methylbenzoate	**Cu**_**7**_ **4MBz**
$\left[{\mathrm{Cu}}_{7}{\left(\mu ‐\mathrm{adeninato}\right)}_{6}{\left(\mu_{3}‐\mathrm{OH}\right)}_{6}{\left(\mu ‐{\mathrm{OH}}_{2}\right)}_{6}\right]\cdot {\left(C_{8}H_{7}O_{2}\right)}_{2}\cdot 10H_{2}O$	4-methylbenzoate	**Cu**_**7**_ **4MBz_D**
$\left[{\mathrm{Cu}}_{7}{\left(\mu ‐\mathrm{adeninato}\right)}_{6}{\left(\mu_{3}‐\mathrm{OH}\right)}_{6}{\left(\mu ‐{\mathrm{OH}}_{2}\right)}_{6}\right]\cdot {\left(C_{11}H_{7}O_{2}\right)}_{2}\cdot 12H_{2}O$	naphthalene-2-carboxylate	**Cu**_**7**_ **2Naph**
$\left[{\mathrm{Cu}}_{7}{\left(\mu ‐\mathrm{adeninato}\right)}_{6}{\left(\mu_{3}‐\mathrm{OH}\right)}_{6}{\left(\mu ‐{\mathrm{OH}}_{2}\right)}_{6}\right]\cdot {\left(C_{11}H_{7}O_{2}\right)}_{2}\cdot 11H_{2}O$	naphthalene-2-carboxylate	**Cu**_**7**_ **2Naph_D**
$\left[{\mathrm{Cu}}_{7}{\left(\mu ‐\mathrm{adeninato}\right)}_{6}{\left(\mu_{3}‐\mathrm{OH}\right)}_{6}{\left(\mu ‐{\mathrm{OH}}_{2}\right)}_{6}\right]\cdot {\left(C_{13}H_{9}O_{2}\right)}_{2}\cdot 12H_{2}O$	biphenyl-4-carboxylate	**Cu**_**7**_ **4Bi**
$\left[{\mathrm{Cu}}_{7}{\left(\mu ‐\mathrm{adeninato}\right)}_{6}{\left(\mu_{3}‐\mathrm{OH}\right)}_{6}{\left(\mu ‐{\mathrm{OH}}_{2}\right)}_{6}\right]\cdot {\left(C_{13}H_{9}O_{2}\right)}_{2}\cdot 9H_{2}O$	biphenyl-4-carboxylate	**Cu**_**7**_ **4Bi_D**
$\left[{\mathrm{Cu}}_{7}{\left(\mu ‐\mathrm{adeninato}\right)}_{6}{\left(\mu_{3}‐\mathrm{OH}\right)}_{6}{\left(\mu ‐{\mathrm{OH}}_{2}\right)}_{6}\right]\cdot {\left(C_{12}H_{15}O_{2}\right)}_{2}\cdot 8H_{2}O$	4-pentylbenzoate	**Cu**_**7**_ **4PBz**

**Table 2:** Main Crystallographic Data of the Compounds

	**Cu**_**7**_ **Bz**	**Cu**_**7**_ **3MBz**	**Cu**_**7**_ **4MBz**	**Cu**_**7**_ **2Naph**	**Cu**_**7**_ **4Bi**	**Cu**_**7**_ **4PBz**
***a***	31.2961(8)	30.9041(7)	30.7143(6)	31.4361(7)	31.5881(7)	31.0890(5)
***b***	31.2961(8)	30.9041(7)	30.7143(6)	31.4361(7)	31.5881(7)	31.0890(6)
***c***	20.9992(3)	20.8746(4)	20.8768(2)	20.8606(3)	20.7720(4)	20.7700(4)
***V* (Å** ^**3**^ **)**	17812.0(9)	17265.6(10)	17055.9(7)	17853.1(8)	17949.6(9)	17385.2(7)
***V*/*Z***	1979.1	1918.4	1895.1	1983.7	1994.4	1931.7
**voids (%)**	29	21	19	20	18	12

	**Cu**_**7**_ **Bz_D**	**Cu**_**7**_ **3MBz_D**	**Cu**_**7**_ **4MBz_D**	**Cu**_**7**_ **2Naph_D**	**Cu**_**7**_ **4Bi_D**	
***a***	30.4500(3)	30.7245(10)	30.4929(7)	30.9597(10)	31.2649(5)	
***b***	30.4500(3)	30.7245(10)	30.4929(7)	30.9597(10)	31.2649(5)	
***c***	21.0004(2)	20.8620(5)	20.8902(4)	20.8368(5)	20.7654(3)	
***V* (Å** ^**3**^ **)**	16862.9(4)	17056.0(1)	16821.7(8)	17296.4(1)	17578.6(6)	
***V*/Z**	1873.7	1895.1	1869.1	1921.8	1953.2	
**voids (%)**	22	21	17	17	17	

All compounds incorporate the centrosymmetric cationic [Cu_7_(μ-adeninato)_6_(μ_3_-OH)_6_(μ-OH_2_)_6_]^2+^ discrete building block^26,28,31,32^ whose charge is counterbalanced by aromatic benzoate monocarboxylic derivative anions. The heptameric building unit exhibits a wheel-shaped moiety with a central [Cu­(OH)_6_]^4–^ core connected to six outer copper­(II) metal centers by μ_3_–OH bridges in a radial and planar arrangement (Figure 3a,b). This discrete entity constitutes a robust disk with approximate dimensions of 18 × 18 × 12 Å^3^, defined by its van der Waals surface. The external copper atoms are connected by a double bridge of semicoordinated water molecules and peripheral adeninato ligands with a bidentate μ-κ*N*3:κ*N*9 coordination. The copper­(II) centers show an octahedral geometry with the characteristic Jahn–Teller tetragonal elongation, a feature that is more pronounced for the external Cu atoms than for the inner ones due to the lower rigidity of their coordination environment.

**Figure 3:**
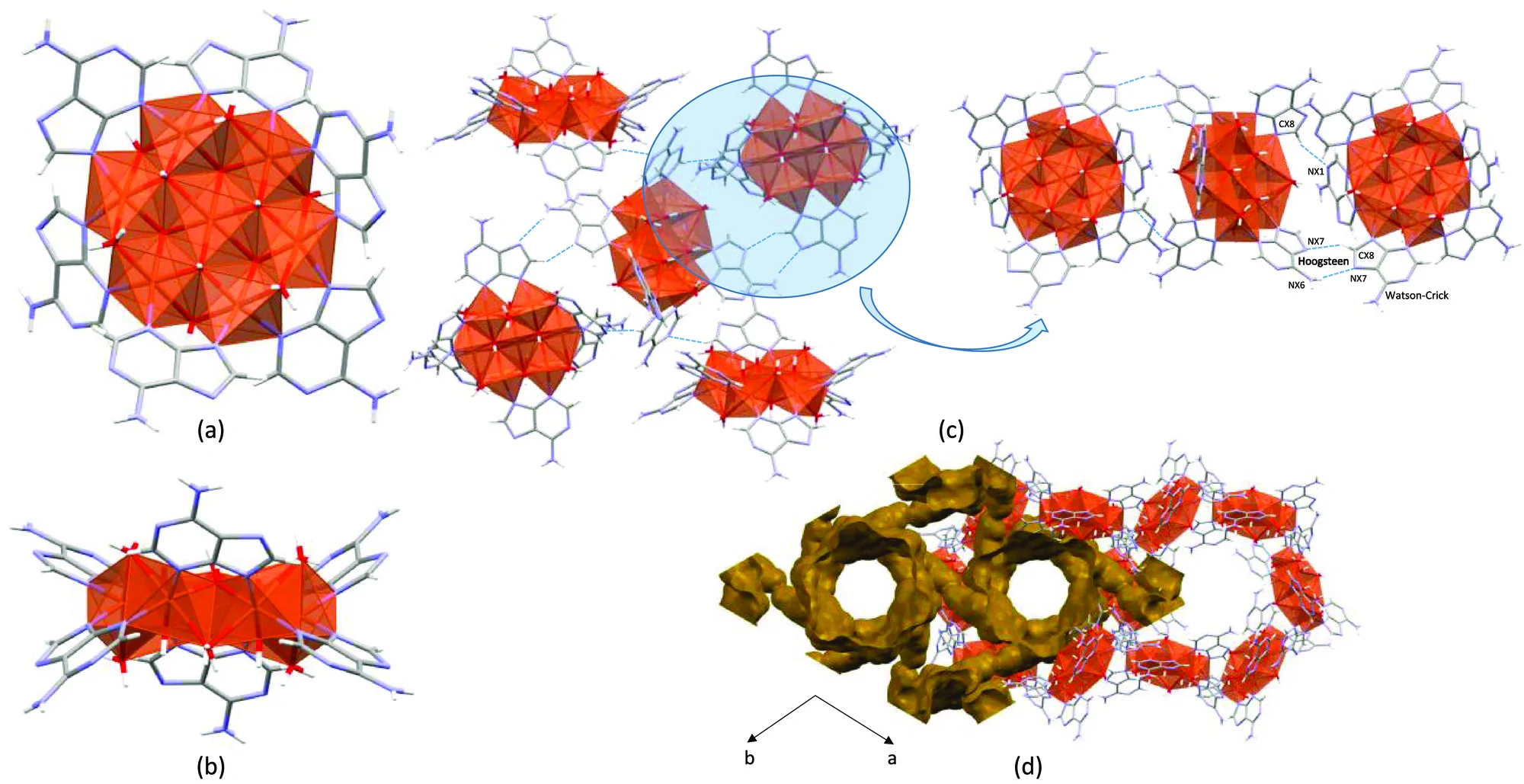
Heptanuclear entity [Cu_7_(μ-adeninato)_6_(μ_3_-OH)_6_(μ-OH_2_)_6_]^2+^: (a) front and (b) side (Color code: H, white; C, grey; N, blue; O, red; and Cu, orange), (c) hydrogen-bonding interaction that connect adjacent cationic entities in the compounds and (d) schematic packing showing the pores without organic anions.

All members of the isostructural family exhibit identical supramolecular interaction patterns between the cationic entities (Figure 3c). In contrast to analogous isoreticular compounds that contain the heptameric Cu_7_ cation,^26,31,32^ no π–π stacking interactions are observed between adeninato ligands. The supramolecular assembly of the copper-adeninato units is only governed by complementary hydrogen-bonding interactions with adeninato ligands from four adjacent heptameric clusters, forming an *R*_2_^2^(8) hydrogen-bonded ring. This arrangement involves the Hoogsteen face of the adeninato ligands (N–H···N) interacting with a neighboring imidazolic atoms (N···H–C). These intercluster connections are reinforced by an additional C–H···N to form a helicoidal arrangement with an internal pore (Figure 3d).

Considering the aforementioned supramolecular interactions, the 3D configuration of all the compounds can be topologically characterized as a uninodal four-connected *nbo* network.^35–37^ The nodes are attributed to the heptameric units, while the linkers correspond to the previously delineated hydrogen bond interactions. The total volume of this hypothetical empty framework for the case of **Cu**_**7**_ **Bz**, excluding the anionic counterions, represents the 43% of the unit cell. These interconnected channels are partially occupied by the benzoate anions, which reduce the empty space but do not occlude the pores, even when the size of the organic anion is increased (Figure 4). Therefore, the supramolecular interactions that define the porosity of the framework do not directly involve the monocarboxylate anions. This is in stark contrast to the previously reported isoreticular family containing dicarboxylate anions, where the organic anions are intercalated between the π-stacked adeninato ligands via hydrogen-bonding and π–π stacking interactions, directly determining the shape of the porous structure with a *pcu* topology.^31^

**Figure 4:**
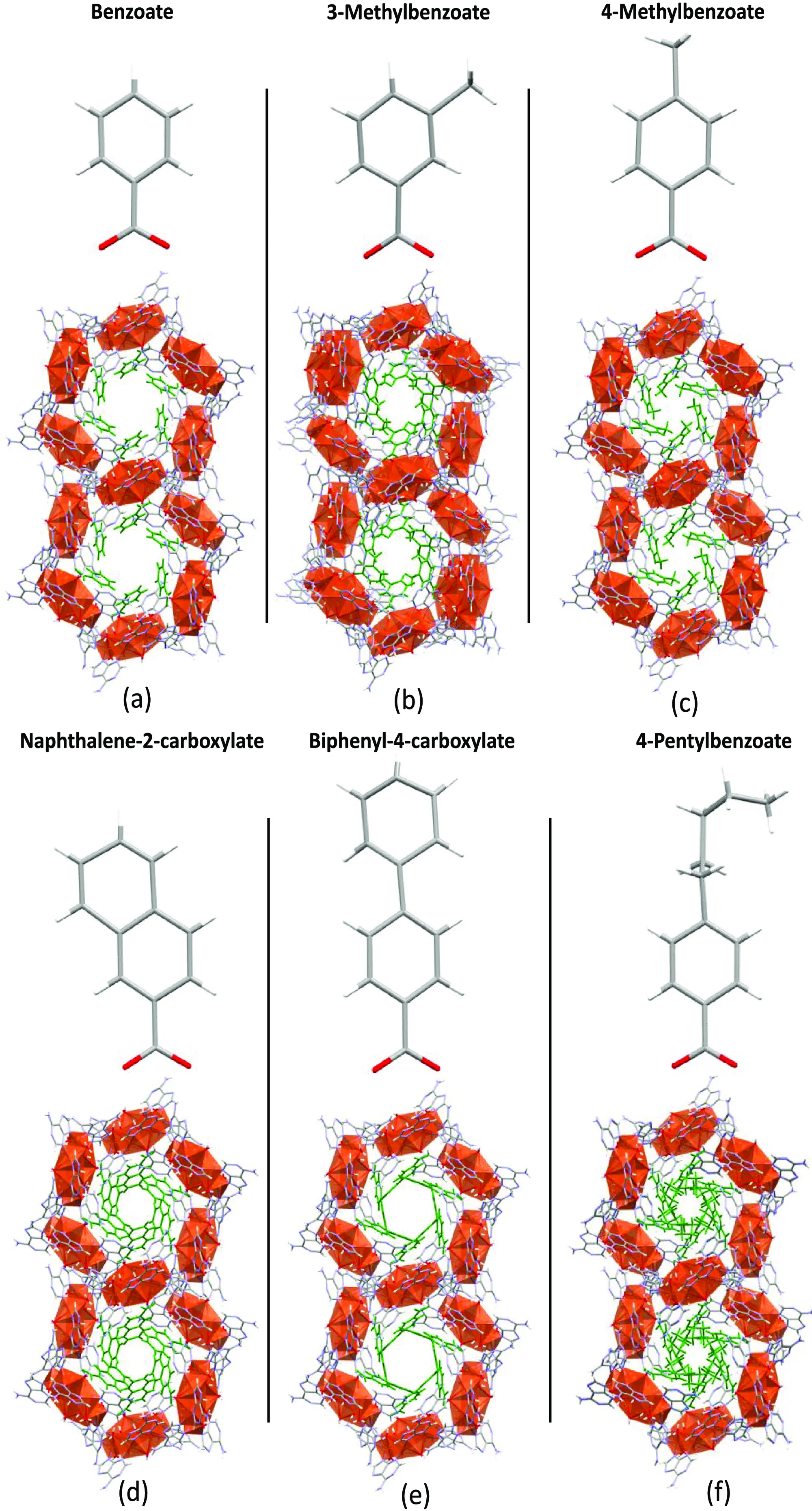
(a–f) Summary of the packing of the different benzoate derivative compounds with the anion highlighted in green for a better view.

As shown in Figure 5, the carboxylate anions interact with the inorganic clusters through O–H···O hydrogen-bonding interactions, involving the oxygen atoms of carboxylate group as hydrogen bond acceptors, and hydrogen atoms of both a hydroxide group and a coordinated water molecule as donors (Table S4 and Figure S4). This engagement of the anions is reinforced by π–π stacking between the organic anions and the aromatic rings of the adeninato ligands, involved in double N–H···N hydrogen bond. The strength of these interactions depends strictly on the features of the anion (Table S6).

**Figure 5:**
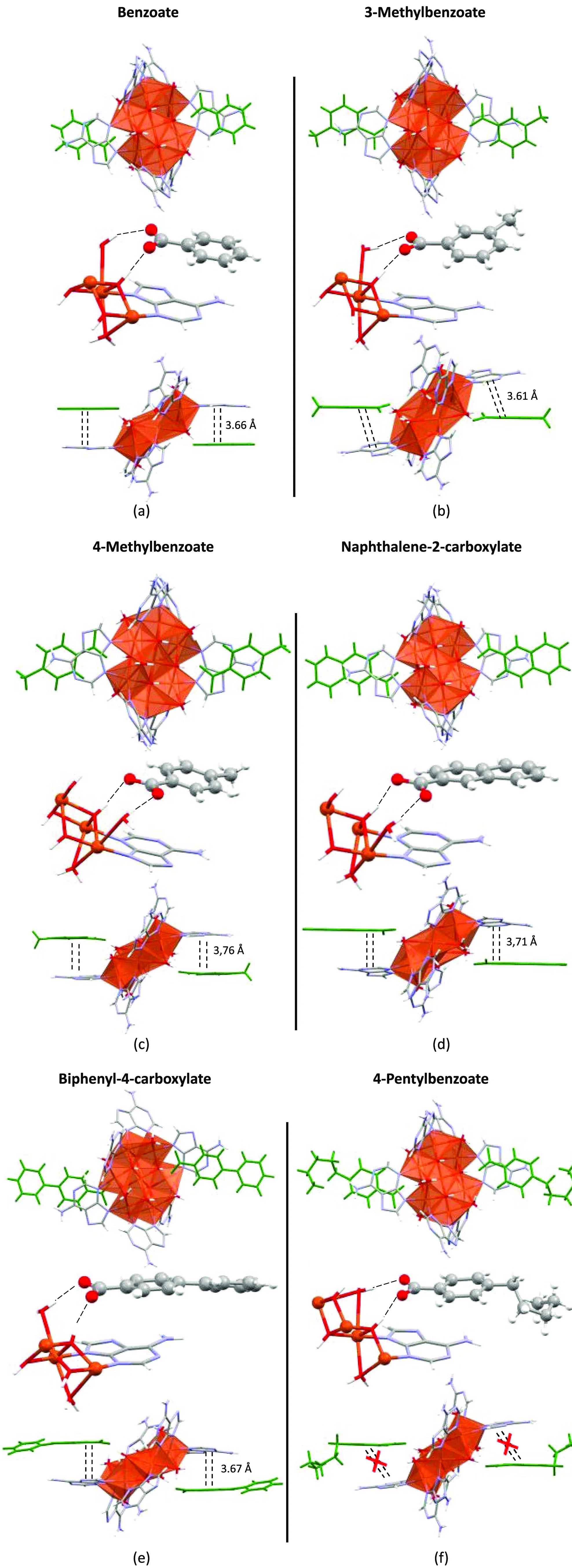
Supramolecular interaction between the adeninato ligands and the benzoate derivative anions in all the compounds.

All these supramolecular interactions give rise to channels along the c-axis with an irregular shape, exhibiting maximum dimensions ranging from 6.78 Å for the **Cu**_**7**_ **2Naph** compound to 5.34 Å for the **Cu**_**7**_ **4PBz** compound. These channels intersect through narrower corridors, with *d*_min_ decreasing from 4.00 Å for the unsubstituted **Cu**_**7**_ **Bz** compound to 1.89 Å for the **Cu**_**7**_ **4PBz** analogue (Figures S7–S8). The total channel volumes, partially occupied by crystallization water molecules, range from 5098 (**Cu**_**7**_ **Bz**) to 2054 Å^3^ (**Cu**_**7**_ **4PBz**), corresponding to an accessible void fraction ranging from 29 to 12% (Table S8).

Complementary computational analyses indicate that the specific surface area decreases accordingly, with values between 205 to below 20 m^2^·g^–1^ for the most voluminous derivative system. The calculated pore volumes fall within the 0.200–0.075 cm^3^·g^–1^ range, with the benzoate-containing material exhibiting the highest values, Figures 6 and S6.

**Figure 6:**
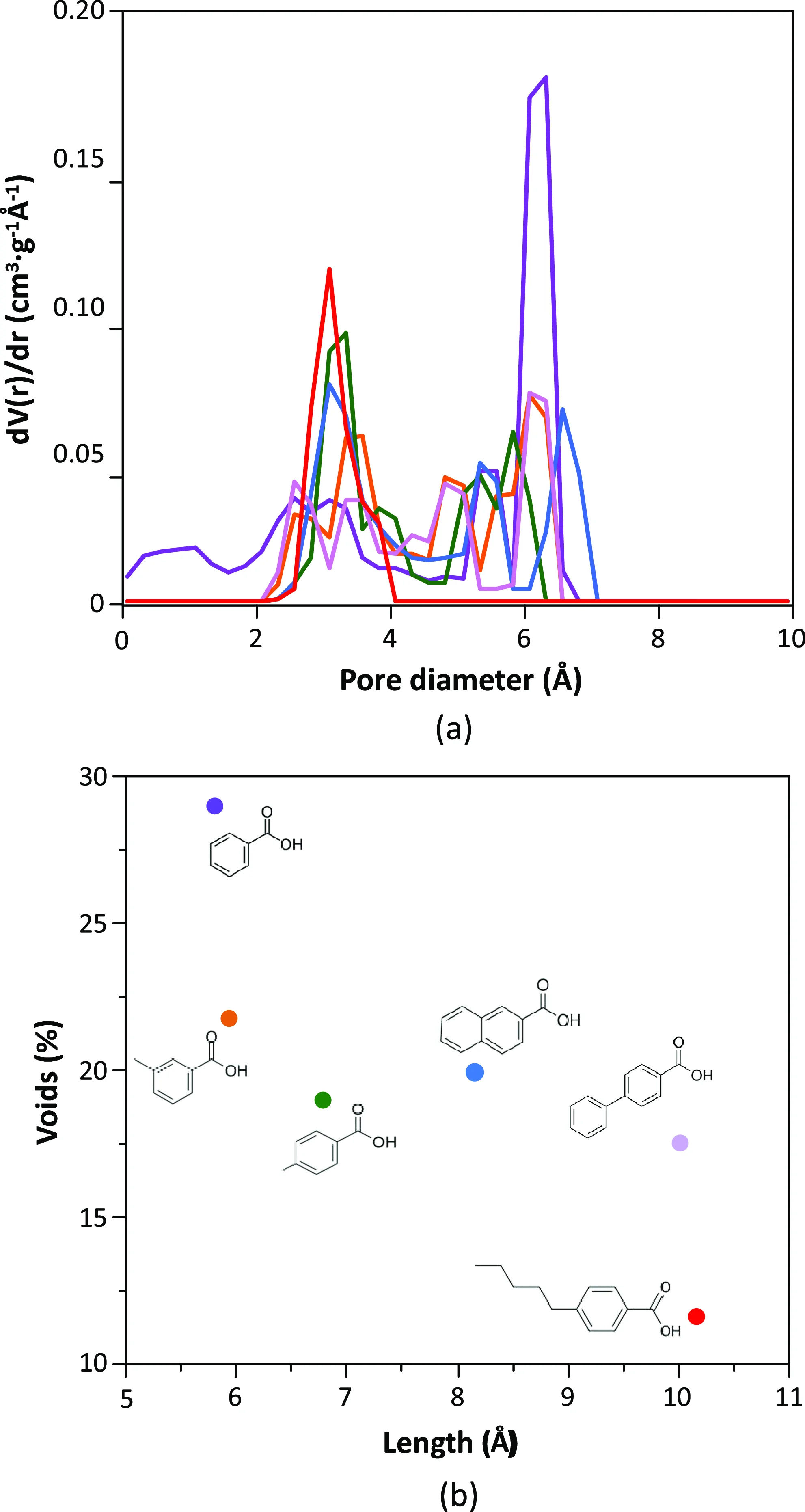
(a) Pore size distribution for compounds **Cu**_**7**_ **Bz** (purple), **Cu**_**7**_ **3MBz** (orange), **Cu**_**7**_ **4MBz** (green), **Cu**_**7**_ **2Naph** (blue), **Cu**_**7**_ **4Bi** (pink) and **Cu**_**7**_ **4PBz** (red). (b) Correlation between the length of the monocarboxylate counterion and the void percentage.

However, as previously mentioned, these supramolecular compounds exhibit a high degree of structural flexibility due to the relatively weak nature of the interactions between the heptameric entities. This phenomenon was verified by resolving the crystal structures of the single crystals obtained after filtration, which retain the features of the overall supramolecular architectures but exhibit a decrease in both crystallization water molecules and unit cell volume (Table S2, Figures S9–S10). For example, in the case of benzoate-containing compounds (**Cu**_**7**_ **Bz** and **Cu**_**7**_ **Bz_D**), the number of crystallization water molecules per formula unit decreases from 17 to 13, the unit cell volume reduces by 5% with a reduction of almost 1000 Å^3^ (17812 and 16863 Å^3^) and the accessible pore fraction shrinks from the initial 7652 to 6670 Å^3^, (29 to 22%) (Figure 7). This process can be observed for the other compounds, although to a lower extent. In all structures, due to the limited free space, supramolecular interactions intensify as the clusters move closer together following slight positional changes, as can be seen in the H-bonds and π–π stacking between the clusters and the anion in each case, as illustrated in Tables S5 and S7 and Figure S5.

**Figure 7:**
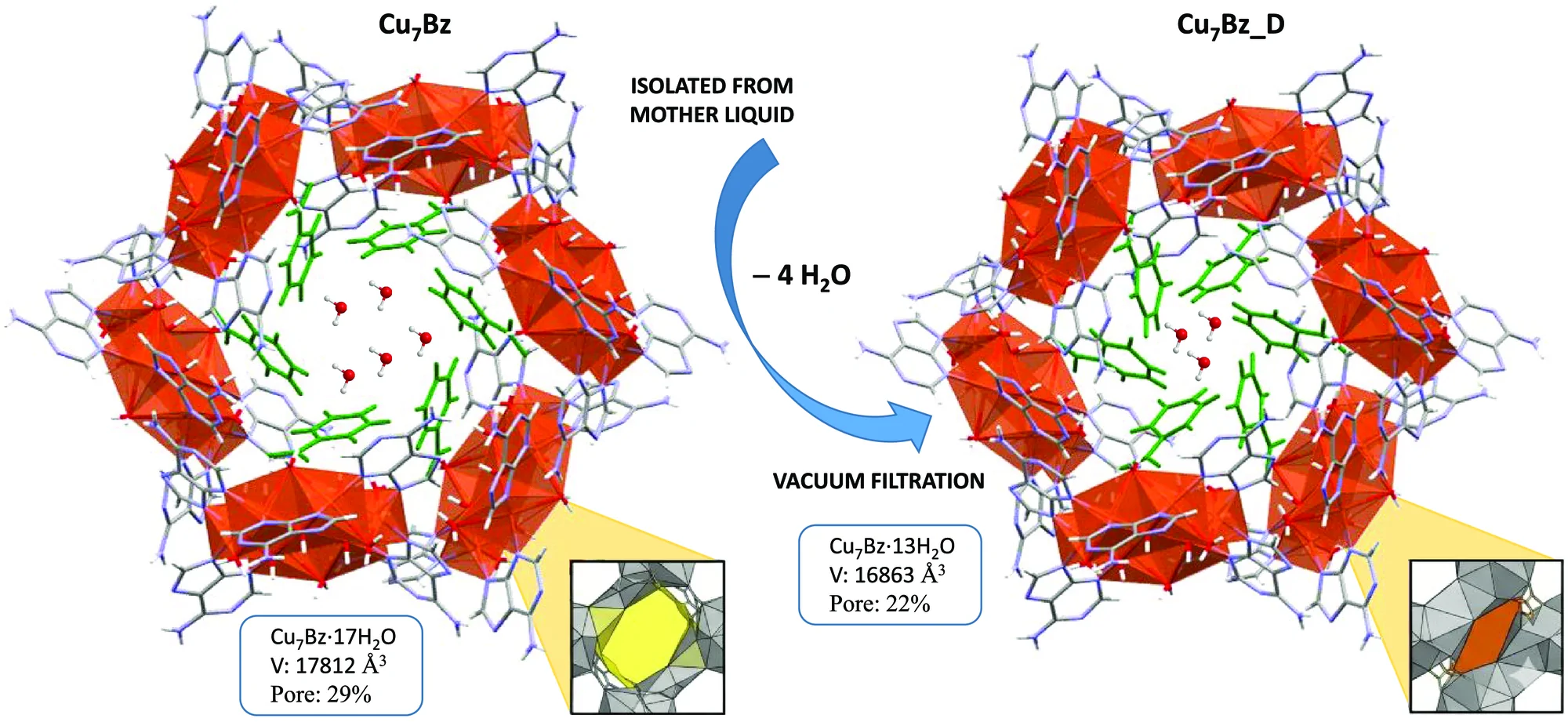
Description of the flexible structure of this isostructural compounds with the pore shrinkage when the sample is filtered and measured by SC-XRD.

### 2.2 Water Sorption Properties and Structural Flexibility

Crystallographic analysis of the series of compounds studied herein confirms that the nature of the monocarboxylate anion employed during synthesis is the primary factor determining the percentage of free void space and the subsequent adsorption capacity of the resulting supramolecular architectures. Water vapor adsorption/desorption isotherms for previously outgassed samples of the compounds (Figure 8 and S11) reveal a systematic correlation between the maximum water uptake and the steric hindrance introduced by the chemical nature of the organic anion.

**Figure 8:**
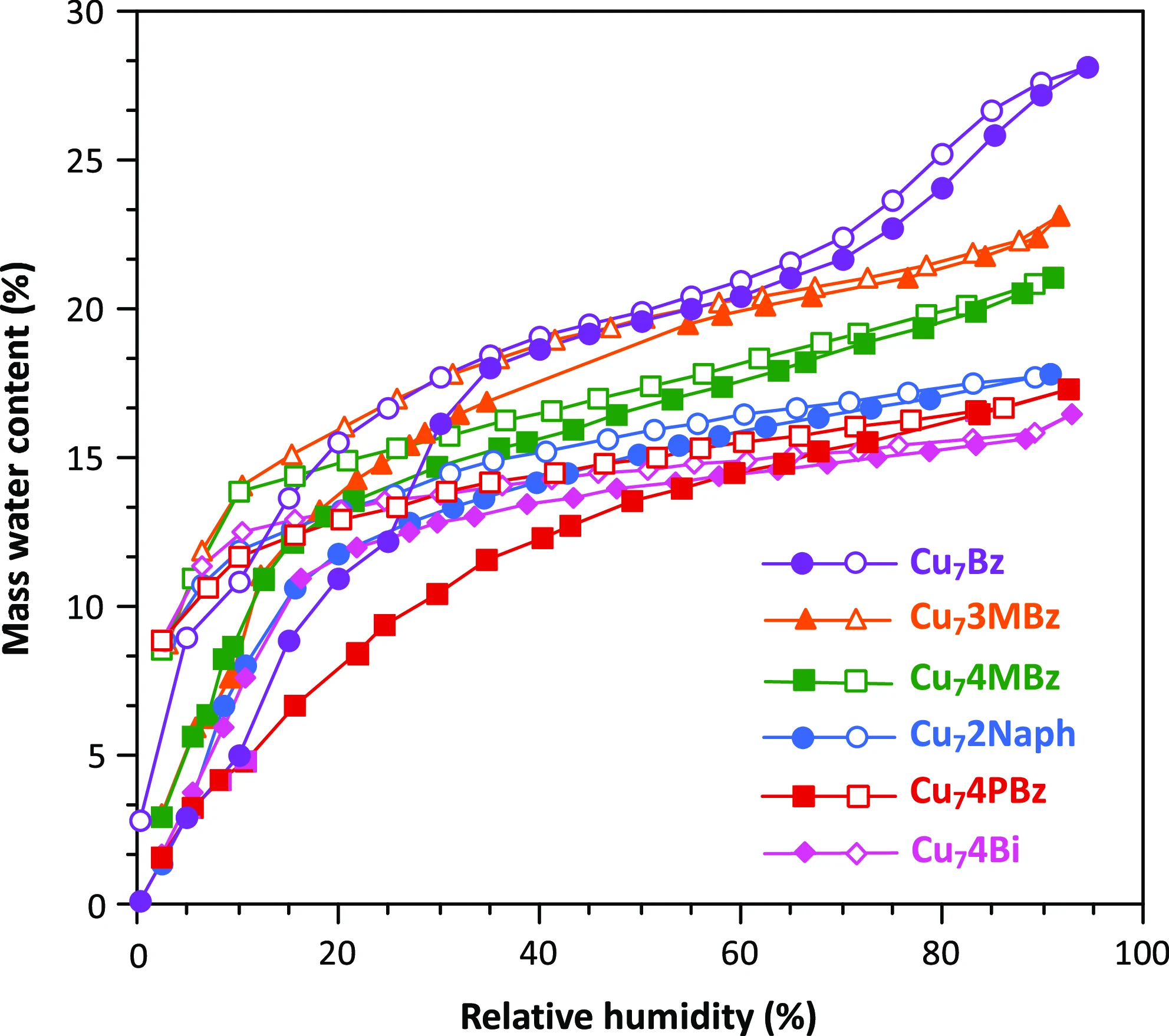
First water cycling of adsorption/desorption isotherms at 20 °C for an activated sample of **Cu**_**7**_ **Bz** (purple circle), **Cu**_**7**_ **3MBz** (orange triangle), **Cu**_**7**_ **4MBz** (green square), **Cu**_**7**_ **2Naph** (blue circle), **Cu**_**7**_ **4PBz** (red square) and **Cu**_**7**_ **4Bi** (pink diamond).

It is important to emphasize that in this isostructural series, the variations in water uptake cannot be attributed solely to the crystallographic aperture dimensions. By systematically varying the monocarboxylate counterion, both the physical pore volume (steric accessibility) and the local pore chemistry (hydrophilicity/hydrophobicity balance) are modified simultaneously. Therefore, the observed Type II and III isotherms^38^ which are characteristic of materials with weak initial adsorbent–adsorbate interaction, reflect the synergistic effect of this complex pore microenvironment rather than isolated geometric constraints. In these systems, the interaction between the water molecules and the pore walls is comparatively low relative to the cohesive forces of the water molecules themselves (H_2_O···H_2_O interactions). This behavior is particularly evident in the low relative humidity (RH) regime (0–20%), where a modest initial adsorption occurs at the highest-energy sites. This is followed by a transitional region, from 20 to 60% (RH), which finalizes with an exponential increase in uptake between 60 and 90% RH for **Cu**_**7**_ **Bz**. This final stage is indicative of capillary condensation and the formation of molecular multilayers, a phenomenon typical of mesoporous or macroporous materials where the pores are large enough to allow for bulk-like liquid behavior of the adsorbate.

It was found that all of the compounds exhibited residual water retention, with levels ranging from 3–8%, at zero humidity values. This amount of unreleased water is close to that expected for the coordination water molecules (∼6%). Furthermore, an analysis of the data reveals that certain compounds, such as **Cu**_**7**_ **Bz** and **Cu**_**7**_ **4PBz**, exhibit a water adsorption hysteresis loop at lower values of relative humidity. The hysteresis likely arises from a combination of factors: the physical retention of water within the narrowest parts of the pores, cooperative water–water interactions that stabilize the liquid phase during desorption, and a degree of structural rearrangement. This behavior has been observed in previous compounds within our group, which have been demonstrated to exhibit more pronounced hysteresis curves than those reported in this paper.^31^

This difference can be attributed to the breathing behavior of the supramolecular compounds. The flexibility is evident in the maximum adsorption data for each compound. Assuming that the coordinating water molecules are lost with each activation, the maximum theoretical value for the **Cu**_**7**_ **Bz** compound, the most porous one, should be 19%. However, it adsorbs 28%, which is equivalent to around 25 water molecules, 8 more than the most hydrated phase observed via SC-XRD. This phenomenon is not exclusive to the **Cu**_**7**_ **Bz**; it is also observed in the other derivatives, which exhibit higher adsorption than would be predicted from SC-XRD data, accommodating an average of eight additional water molecules observed in each case, (Table S9). Nevertheless, in the current Cu_7_–SMOF series, the low percentage of void space and the specific packing of the anions appear to exert a stabilizing effect that limits the magnitude of this structural breathing. Consequently, a comparison of the PXRD patterns of the samples in different stages (dry *vs.* humid conditions) does not show the dramatic structural shifts observed in more open architectures (Figures S18 and S19). The framework under consideration is sufficiently flexible to accommodate the observed hysteresis, yet rigid enough to preserve its long-range order without substantial macroscopic deformation.

As the organic fragment of the anion becomes more voluminous, the available internal space for guest molecules is progressively sacrificed to accommodate the anionic backbone within the crystal lattice, for example, being constrained to 18% for the bulky biphenyl-4-carboxylate. Materials containing smaller and less hydrophobic anions, such as benzoate and methylbenzoate derivatives (**Cu**_**7**_ **Bz**, **Cu**_**7**_ **3MBz**, and **Cu**_**7**_ **4MBz**), exhibit higher water uptake over the entire relative humidity range, 28, 23, and 21% respectively. These anions impose limited steric hindrance and allow greater accessibility of water molecules to the internal adsorption sites. While **Cu**_**7**_ **3MBz** and **Cu**_**7**_ **4MBz** share the same chemical formula, the shift of the methyl group from the *meta-* to the *para*- position results in a reduction in water uptake of 2%, and a decrease in void volume from 21 to 19%. The structural implication is that the *para*-substitution likely forces a denser packing of the aromatic rings, whereas the *meta*-substitution allows for a slightly more staggered arrangement, preserving a larger percentage of free void space.

The analysis of the pore size distribution (PSD), Table S8 and Figure S6, provides the physical justification for these observations. In **Cu**_**7**_ **Bz**, the relatively wide minimum aperture of 4.00 Å facilitates the entry of water molecules, leading to this high water uptake. However, in the substituted isomers, *d*_min_ drops significantly to 2.43 and 1.84 Å for **Cu**_**7**_ **3MBz** and **Cu**_**7**_ **4MBz**, respectively. This geometric difference introduces a subtle bottleneck effect similar to that described in our previous studies.^31^ Given that the kinetic diameter of water is ∼2.65 Å,^39^ these narrow windows technically preclude guest entry in a rigid state. Thus, the observed adsorption in **Cu**_**7**_ **4MBz** and **Cu**_**7**_ **4PBz** (*d*_min_ 1.89 Å) confirms that these apertures must undergo a transient expansion, while the *d*_max_ values, 6.78 and 6.53 Å for **Cu**_**7**_ **2Naph** and **Cu**_**7**_ **Bz**, respectively, define the internal ellipsoidal cavities where the bulk of the water is stored. The restricted *d*_max_ of 5.34 Å for **Cu**_**7**_ **4PBz** further explains why it exhibits the most limited capacity despite its flexible nature.

Furthermore, the hydrophobicity introduced by anionic substitutions, such as naphthalene-2-carboxylate, biphenyl-4-carboxylate, and 4-pentylbenzoate, plays a decisive role in the sorption features, resulting in significantly lower water adsorption capacities. For instance, the **Cu**_**7**_ **4PBz** (17%) and **Cu**_**7**_ **4Bi** (16%) show the lowest adsorption capacities. This is not merely a consequence of the reduced physical volume but also a result of the chemical environment of the pores. The increased aromatic extension or the presence of long alkyl chains have been demonstrated to enhance the hydrophobic character of the framework. This may partially block or narrow the diffusion pathways, thereby reducing the affinity for water molecules by creating a barrier for the initial nucleation of water clusters. This explains why the water uptake in these materials remains lower at low humidity values compared to the pristine **Cu**_**7**_ **Bz**. It is important to note that the hydrophobic character of substituents like the pentyl chain in **Cu**_**7**_ **4PBz** is most evident in the low-RH regime (0–40%), where it exhibits the lowest initial uptake of the series due to a higher energy barrier for water cluster nucleation, whereas its final capacity at high RH is governed by the structural flexibility and total expandable volume.

In contrast to the abrupt steps observed in rigid-pore MOFs, these SMOFs exhibit a gradual smooth transition, suggesting that water adsorption is accompanied by minor internal adjustments to the orientation of the organic chains of the anions.

Additionally, differences in the initial number of crystallization water molecules suggest variations in the intrinsic hydrophilicity of the frameworks. Materials with higher initial water content tend to show enhanced water uptake, likely due to the presence of more favorable hydrogen-bonding environments that facilitate water adsorption at increasing relative humidity (Figure S9).

In summary, the sorption capacity of this SMOF family can be defined as a delicate balance between the physical dimensions of the anion, its inherent flexibility, geometry, and the chemical nature (hydrophobicity) of its substituents. This underscores the critical function of peripheral organic components in modulating the sorption characteristics of metal–organic materials, illustrating how minimal molecular mobility can compensate for constrained internal volume.

### 2.3 Quantitative Analysis of Drug Loading-Delivery via Magnetic Sustentation

Following the confirmation of the isostructural SMOF family’s aptitude for water vapor adsorption, coupled with their exceptional chemical stability and insolubility in aqueous media, we evaluated their performance as nanocarriers for pharmaceutically active molecules (Figure 9). We selected three drugs with distinct therapeutic profiles: the antitumoral 5-fluorouracil (5-FU), the antibiotic cycloserine (CYCLO), and the anticonvulsant gabapentin (GABA).

**Figure 9:**
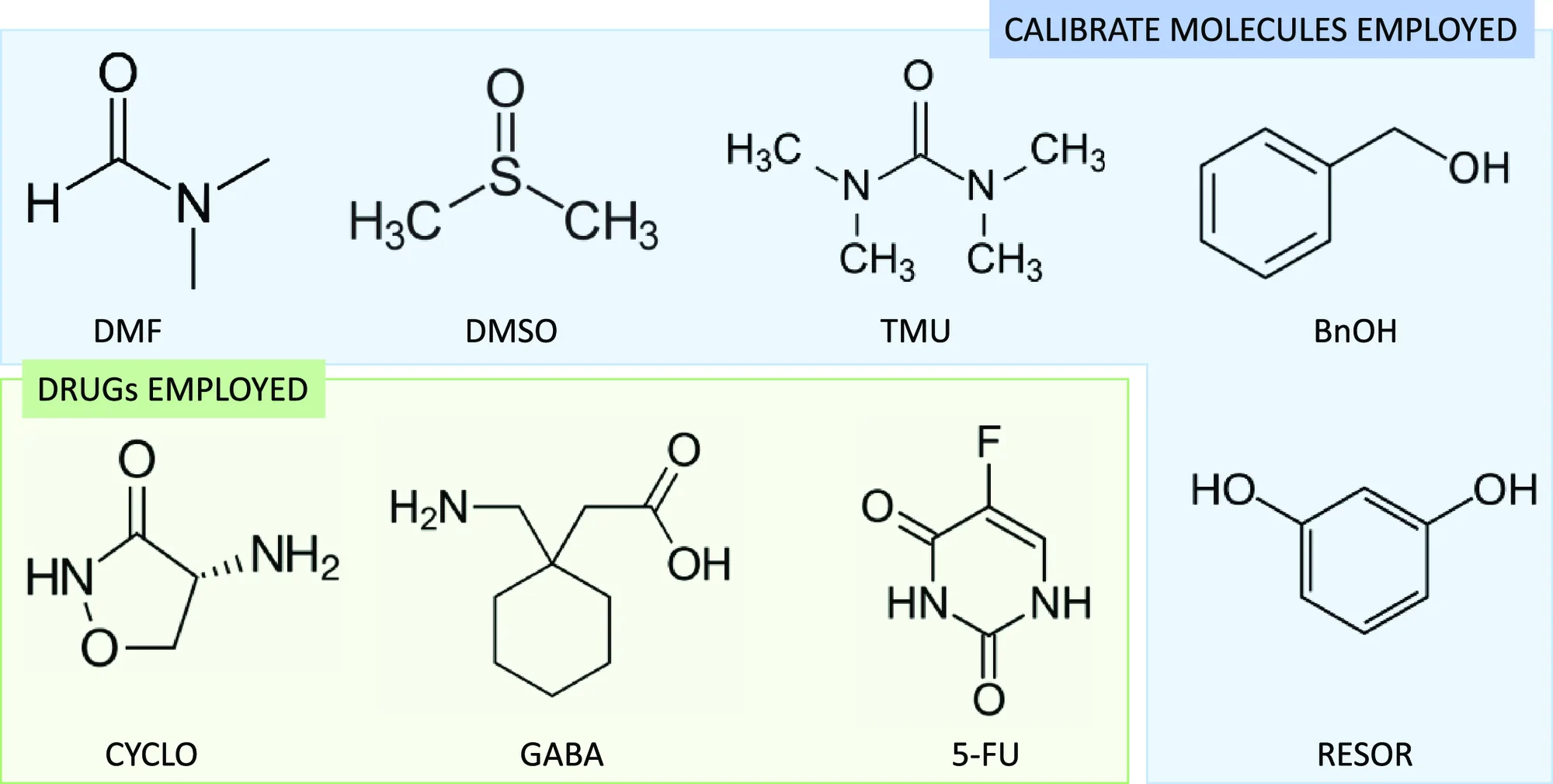
Molecules employed in the drug loading procedure.

Quantifying drug uptake in porous solids remains an analytical challenge for therapeutic agents exhibiting poor aqueous solubility. This limitation frequently precludes the use of conventional spectroscopic or chromatographic techniques, such as ^1^H-NMR or HPLC, for direct concentration measurements in the supernatant, often necessitating complex digestion or extraction protocols that can compromise sample integrity. There is an urgent need for nondestructive, physical techniques capable of quantifying guest molecules within micro-quantities of porous matrices. To address this, we employed a magnetic sustentation procedure recently developed by our group.^40,41^ It is noteworthy that all supramolecular compounds containing the heptameric [Cu_7_(μ-adeninato)_6_(μ_3_-OH)_6_(μ-OH_2_)_6_]^2+^ entity manifest ferrimagnetic behavior at low temperatures; however, this technique leverages the paramagnetic response of the SMOF at room temperature to quantify the mass of guest molecules incorporated within the pores.

The technique setup consists of a vertical magnetic gradient generated by a permanent magnet. The fundamental principle of the technique relies on the measurement of the critical magnetic field (*H*_crit_) which is the specific field strength required to magnetically trap the particles against the poles of the magnet. During measurement, the particles are suspended in a tube with aqueous media within the device. The stability of the trapped particles is governed by the equilibrium of three vertical forces: the magnetic force (*F*_*m*_), the gravitational force (*F*_*g*_), and the buoyancy (*F*_*b*_) (eq 1). The equilibrium condition at the point of release is defined as

1 $$F_{m}=F_{g}-F_{b}$$

By precisely varying the critical lateral displacement parameter (*D*) between the sample and the magnet, the magnetic gradient is adjusted until the magnetic force can no longer counteract the apparent weight of the particles. The precise distance at which the particles fall from the magnetic trap defines the critical distance (*D*_crit_), which is directly proportional to the critical magnetic field (*H*_crit_). Since the incorporation of non-magnetic drugs alters both the effective density and the magnetic response per unit of mass of the composite, *H*_crit_ becomes a highly sensitive indicator of the loading percentage.

It should be noted that not all of these compounds were evaluated in the course of these experiments; priority was given to those with the highest porosity within the structure and the highest percentage of empty space in order to obtain better results. Consequently, **Cu**_**7**_ **4PBz**, which exhibits an empty space percentage of merely 12%, less than half of the **Cu**_**7**_ **Bz** compound (29%), was excluded from this study.

To bypass the solubility issues of the target drugs and prior to their measurements, the magnetic sustentation device requires a prior calibration procedure using a series of highly soluble molecules: RESOR, TMU, DMF, DMSO, and BnOH. To achieve this, we measure the *D*_crit_ at which the **adsorbate@material** particles detached from the pole of the magnet for known mass percentages of these standards, previously determined by HPLC and a robust linear correlation was obtained (eq 2):

2 $$\%\;mass=A^{\prime }\cdot D_{crit}+B^{\prime }$$

where *A′* and *B′* are constants specific to the framework’s magnetic response. To this end, 50 mg of each compound and 2 mL from a stock aqueous solution (25 mg/mL) from each calibration adsorptive molecule were placed in a vial and kept in a thermostatic bath with gentle shaking for 24 h at 30 °C (Figure S12). Subsequently, the SMOF particles were isolated by centrifugation and transferred to the magnetic sustentation device to quantify the amount of the incorporated calibration molecule.

Once the magnetic response was calibrated, the uptake of the poorly soluble drugs (5-FU, CYCLO, and GABA) for which conventional techniques like HPLC are highly inconvenient without aggressive extraction or digestion protocols, was determined indirectly. By simply measuring the *D*_crit_ of the loaded materials in the sustentation device, the mass of the captured guest molecules was precisely extrapolated from the calibration line. For this process, we follow the above described procedure adding 3 mL of deionized water to a vial in a thermostatic bath with agitation at 30 °C for 24 h to complete the adsorption process.

The adsorbed mass vs distance obtained by the measurements of each SMOFs exhibited a robust linear correlation (Figure 10), enabling the precise quantification of drug uptake for each compound. Herein we present the correlation data and line of the **Cu**_**7**_ **Bz** compound, the ones for the rest SMOFs are listed in the supporting information, (Figure S13, Table 3).

**Figure 10:**
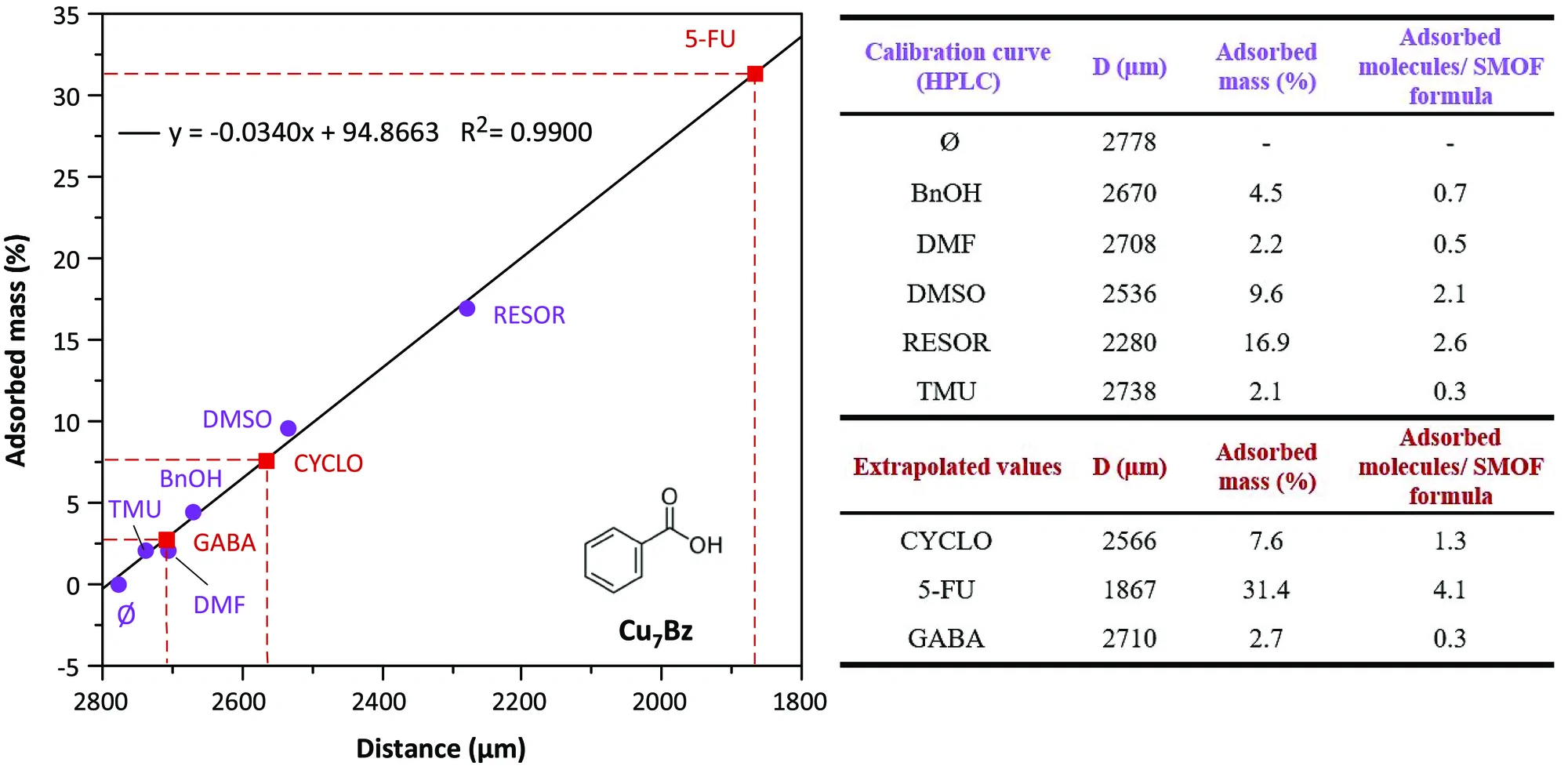
Linear dependence of adsorbed mass (%) vs critical distance (μm). Calibration curves for **Cu**_**7**_ **Bz** obtained by HPLC for the calibration molecules (purple circle), altogether with the extrapolated values for the studied drug molecules (red square). Ø represents the pristine sample of each compound.

**Table 3:** Mass Percentage (%) and Number of Molecules per SMOF Formula of CYCLO, 5-FU, and GABA Adsorbed by **Cu**_**7**_ **Bz**, **Cu**_**7**_ **3MBz**, **Cu**_**7**_ **4MBz**, **Cu**_**7**_ **2Naph**, and **Cu**_**7**_ **4Bi** Compounds

	CYCLO		5-FU		GABA	
	mass (%)	N° molec.	mass (%)	N° molec.	mass (%)	N° molec.
**Cu**_**7**_ **Bz**	7.6 ± 0.2	1.3	31.4 ± 0.3	4.1	2.7 ± 0.2	0.3
**Cu**_**7**_ **3MBz**	4.5 ± 0.1	0.8	24.0 ± 0.3	3.2	1.7 ± 0.1	0.2
**Cu**_**7**_ **4MBz**	3.5 ± 0.2	0.6	5.4 ± 0.2	0.7	1.4 ± 0.1	0.1
**Cu**_**7**_ **2Naph**	2.9 ± 0.2	0.5	5.1 ± 0.1	0.7	1.3 ± 0.1	0.1
**Cu**_**7**_ **4Bi**	3.0 ± 0.2	0.5	5.4 ± 0.2	0.8	1.6 ± 0.1	0.2

The extrapolation of the critical distance in the calibration line provides the loading percentage for these drug molecules which ranges between 1 and 31%. In fact, when analyzing the size, volume and cross-section of all the employed adsorptive molecules (Figures S15 and S16, Tables S10 and S11) which usually are a key factor on the adsorption capacity of the rigid porous materials (most MOFs), no real correlation has been found. However, an analysis of the number of captured drug molecules per formula unit of the SMOF revealed diverse loading capacities. Notably, the highest uptake values were consistently achieved for 5-fluorouracil (5-FU), reaching a maximum in the **Cu**_**7**_ **Bz** framework, which possesses the largest void volume. It is important to note that the high loading value obtained for 5-FU (31.4%) involves an extrapolation beyond the upper limit of our current calibration curve. Therefore, while the linear correlation is robust (*R* ^2^ = 0.9900), this specific value should be interpreted as an estimate with an inherently higher degree of uncertainty compared to the interpolated data.

The discrepancy in the drug loading values of the 5-FU molecule in this isostructural series can be related in a balance, as in the case of the study on water adsorption, to the anion present in each compound, the available pore volume and the specific host-guest interactions. As demonstrated in previous studies, the 5-FU molecule has the capacity to interact with the adeninato ligand within the cluster.^26^ The 5-fluorouracil possesses both hydrogen-bond donors, (N–H) and acceptors (C=O), that can establish complementary interactions with the Hoogsteen and Watson-Crick sides of the adeninato and the oxygen atoms of the carboxylate group of the monocarboxylate counterions. In the case of **Cu**_**7**_ **Bz**, the absence of bulky substituents provides more accessible sites for drug interaction and consequently, adsorption. Instead, as the aromaticity of the counterions increases (naphthalene-2-carboxylate and biphenyl-4-carboxylate in **Cu**_**7**_ **2Naph** and **Cu**_**7**_ **4Bi**, respectively), the enhanced hydrophobic character of the pore environment and the increased steric hindrance not only reduce the effective volume but also modulate the strength of these non-covalent interactions, leading to the observed low values in loading capacity.

After studying the drug uptake for **Cu**_**7**_ **Bz** derivatives, as in previous studies, it was decided to conduct a study of desorption kinetics of the **5-FU@compounds** at 35 °C using the same magnetic sustentation technique (Figures 11 and S14). As 5-fluorouracil was the drug that has been captured in the greater quantity and due to its interest, only 5-FU has been selected for this purpose. To perform this assay, 50 mg of drug loaded samples of compounds were placed in 5 mL of desionized water and the critical distance was measured hourly to monitor the evolution of the drug release. The aqueous media was replaced after each measurement. A first-order kinetic model was applied specifically to the first 6 h of the release profile. This interval was selected as it represents the primary active desorption phase driven by the maximum concentration gradient, thus providing a reliable and consistent baseline to compare the initial sustained release behavior across all samples before equilibrium is approached. The desorption curves indicate that half of the initially loaded drug (*t*_1/2_) is released in the first 3–5 h for 5-FU in all the compounds (Figure 11b).

**Figure 11:**
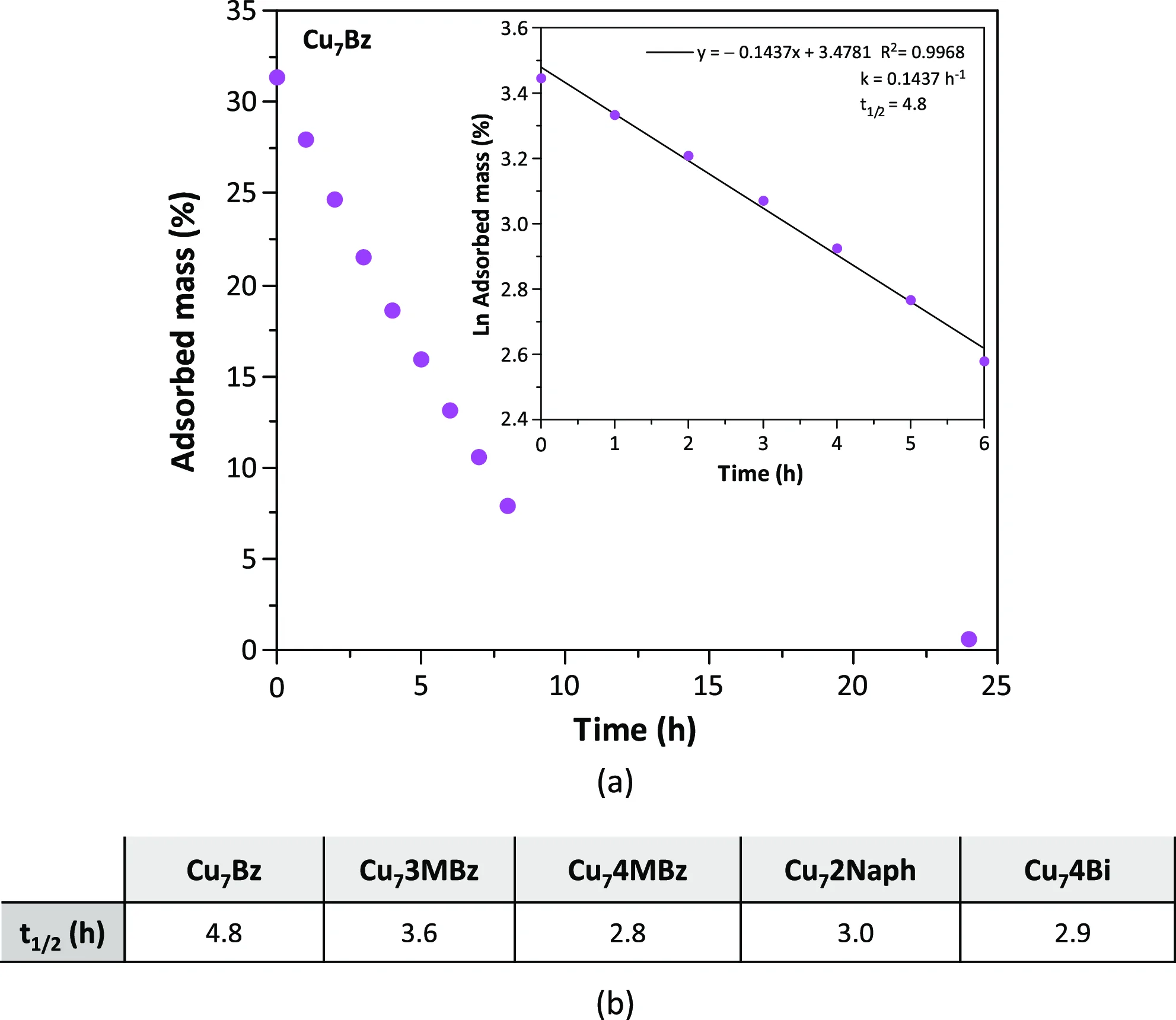
Desorption isotherm in water solution of the 5-FU in (a) **Cu**_**7**_ **Bz** compound. Inset: fitting to a first order kinetic considering the first 6 h of the desorption process. (b) Values in hours of the *t*_1/2_ for all the compounds.

The sustained release values shown in Figures 11 and S14 demonstrate that the drug delivery process is is a complex interplay of several supramolecular factors, significantly influenced but not only dictacted by a “bottleneck” effect created by the functionalized carboxylate groups. In all of the substituted compounds, the average drug release time is significantly shorter, around 3 h, than that of the original benzoate compound, with a value of approximately 5 h. While steric hindrance at the pore window plays a role, relying strictly on the *d*_min/max_ bottleneck effect cannot fully rationalize the experimental trends, a fact clearly illustrated when comparing the positional isomers **Cu**_**7**_ **3MBz** and **Cu**_**7**_ **4MBz**. For instance, comparing the positional isomers, **Cu**_**7**_ **4MBz** possesses the narrowest static aperture (1.84-6.00 Å) but exhibits a shorter release half-time than Cu_7_3MBz, with a value of 2.43–6.48 Å. This apparent contradiction highlights the importance of structural flexibility, specific host-guest interactions, and the initial loading levels. In systems with lower loading capacities (e.g., **Cu**_**7**_ **4MBz**, 5.4%), guest molecules might be predominantly distributed near the pore surface or undergo faster outward diffusion facilitated by transient, guest-induced framework expansion. Conversely, the exceptional loading capacity of **Cu**_**7**_ **Bz** (31.4%) suggests a deeper pore penetration and a more complex network of intermolecular interactions, leading to a more complex diffusion pathway. Consequently, the combination of a higher internal packing density and strong π–π and hydrogen-bonding interactions within the structurally flexible voids of **Cu**_**7**_ **Bz** results in a more prolonged and sustained release profile.

### 2.4 In Vitro Cytotoxicity Assay of 5-FU@Cu_7_Bz on *HCT116* Cells

The antineoplastic agent 5-FU drug is widely employed in the treatment of several types of cancer and has been previously studied by our group regarding its encapsulation in various MOF and SMOF carriers.^42,43^ Cytotoxicity studies were conducted using the **Cu**_**7**_ **Bz** compound, which exhibits the highest drug adsorption capacity. We evaluated the empty framework, the free drug, and the **5-FU@Cu**_**7**_ **Bz** composite, which was thoroughly purified after loading to prevent false positive results caused by traces of non-encapsulated 5-FU in the medium. The aim of these in vitro studies on human colorectal carcinoma cells (*HCT116*) was to understand the biological activity of the SMOF carrier and to identify any potential synergistic effects between the drug and the porous matrix during the release process. The cells were incubated at equivalent active concentration for 72 h: **Cu**_**7**_ **Bz**, 2 μg/100 μL; **5-FU@Cu**_**7**_ **Bz**, 2.84 μg/100 μL; and **5-FU**, 0.84 μg/100 μL, to ensure an objective comparative study.

As shown in Figure 12, the pristine **Cu**_**7**_ **Bz** compound alone induces a slight lag in cell growth comparing with basal condition, a phenomenon previously observed in other SMOFs developed by our research group.^31^ Conversely, the administration of free 5-FU drastically inhibits cell proliferation from the onset, maintaining high toxicity throughout the monitoring period. The drug-loaded material, **5-FU@Cu**_**7**_ **Bz**, exhibits an intermediate behavior, demonstrating greater cytotoxicity performance than the empty **Cu**_**7**_ **Bz** but with a delayed onset compared to the free drug. This delayed effect perfectly correlates with the sustained release profile observed in the desorption curves. Consequently, we can conclude that the **5-FU@Cu**_**7**_ **Bz** system successfully delivers a potent cytotoxic effect while releasing the active molecule progressively. This controlled release is a promising feature for the design of advanced nanocarriers, as delayed release profiles are highly desirable to potentially help manage the narrow therapeutic window typical of highly toxic chemotherapeutics.

**Figure 12:**
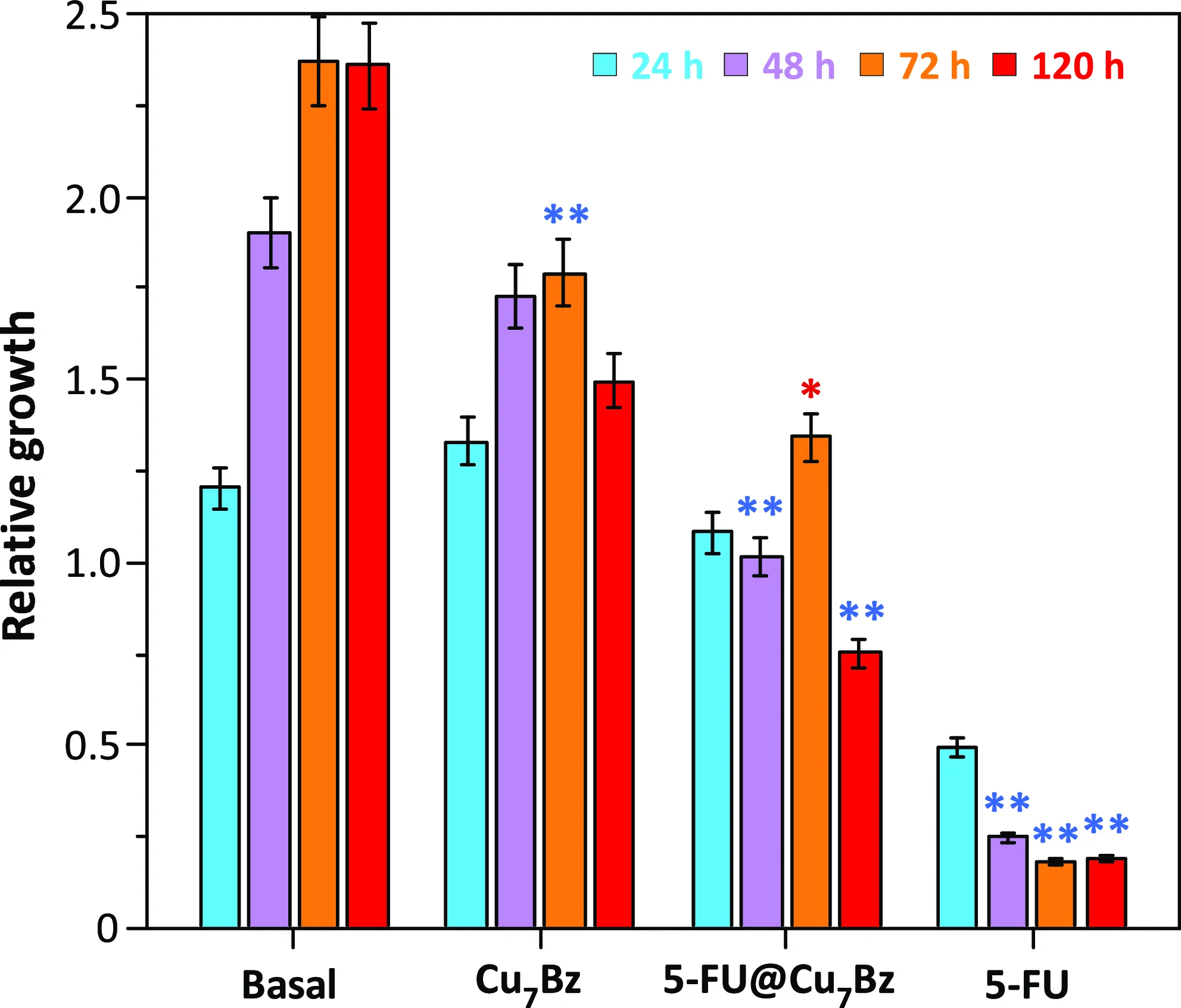
Relative growth of the *HCT116* cells measured by crystal violet at different time points, Figure S17. Cells were grown at basal conditions or after the different compounds were added. Data represent the mean and standard error of at least 4 independent experiments. *p < 0.05 (red); **p < 0.01 (blue) calculated by two-tail, paired Student’s t test against the basal condition.

Considering the aforementioned observations in conjunction with the data from previous studies involving compounds such as **Cu**_**6**_ **Cr** (containing sulfate anion)^27^ and **Cu**_**7**_ **Naph** (featuring larger dicarboxylate anion),^31^ we are able to establish clear structure–activity relationships. In all cases, free 5-FU drug (8.3 x 7.1 x 3.5 Å^3^) eradicates the cell population almost immediately, within the first 24 h. However, when 5-FU is loaded into the materials, the potent cytotoxic effect is delayed until 72–120 h.

The development of **Cu**_**7**_ **Bz** represents a significant structural divergence from the previously reported dicarboxylate-linked SMOF series. The use of monocarboxylates prevents the formation of the highly porous “supramolecular box” motif. Instead, the R3̅ structures adopt a more compact architecture with narrower pores (4.0 × 6.5 Å^2^) compared to the larger voids of the previously reported isoreticular **Cu**_**7**_ **Naph** (9.5 × 11.2 Å^2^). This reduced free volume creates a formidable diffusion barrier, which is essential for a truly controlled drug release.

In the more open framework of the previously reported dicarboxylic **Cu**_**7**_ **Naph**, 5-FU diffuses in and out relatively easily due to the structural flexibility and larger pore dimensions, resulting in an immediate and highly potent cytotoxic effect. This phenomenon elucidates the high potency of the cytotoxic effect of the loaded sample (**5-FU@Cu**_**7**_ **Naph**) from the very beginning. In contrast, in the narrower pores of **Cu**_**7**_ **Bz** (4.0 × 6.5 Å^2^), the drug is “trapped” more effectively. Evidence from analogous systems suggests that this spatial confinement enhances the π–π stacking interactions between the ligand (benzoate) and the drug molecules. While this reduces the total fraction of drug released rapidly, it ensures a constant, sustained level of toxicity without the risks of a sudden “*burst-release*”. Achieving this sustained antiproliferative behavior, rather than an acute toxic shock, is one of the primary and most challenging objectives in modern targeted drug delivery.

## 3 Conclusions

In summary, we have demonstrated an isostructural approach for engineering the internal pore environment of a robust family of copper-adenine supramolecular metal-organic frameworks (SMOFs). By systematically varying monocarboxylate counterions, we achieved fine control over pore dimensions and chemical affinities without altering the underlying global topology, establishing counterion-directed isostructural engineering as a general strategy for the design of functional porous solids. Specifically, our findings highlight that the accessible void space can be finely tuned from 12 to 29% depending on the steric hindrance of the selected anion. Furthermore, the hydrophobicity/hydrophilicity balance introduced by these counterions critically dictates the materials’ water sorption thermodynamics; more hydrophobic and bulky anions (such as naphthalene or biphenyl derivatives) restrict diffusion pathways and lower water uptake, while smaller, less hydrophobic anions (like benzoate) maximize pore accessibility and adsorption capacity.

This structural control was validated functionally using complementary guest molecules to probe highly tunable water vapour sorption behavior, and the quantification of poorly soluble drug loading using a nondestructive magnetic sustentation technique. Regarding drug adsorption, the available pore volume and specific host-guest interactions allowed the optimized **Cu**_**7**_ **Bz** material to achieve an notable 31.4% loading capacity for 5-fluorouracil. Moreover, the compact pore architecture and the structural bottlenecks created by the monocarboxylate anions severely restrict the outward diffusion of the drug, enabling a highly controlled and sustained release capacity compared to more open isoreticular structures.

Furthermore, in vitro assays confirmed that the optimized **Cu**_**7**_ **Bz** framework significantly mitigates the “*burst-release”* effect in *HCT116* cells, ensuring a sustained and potent antiproliferative response that is progressively delivered over 72–120 h. This combined structural and analytical study provides a versatile supramolecular platform for precise pore engineering, but also establishes alternative analytical and synthetic strategies offering reliable tools for the characterization and development of high-performance multifunctional materials across the chemical and biomedical sciences.

## 4 Experimental Section

### 4.1 Chemicals

The following reagents were used for the preparation of the compounds: copper­(II) nitrate trihydrate (Sigma-Aldrich, 98%), adenine (Sigma-Aldrich, > 99%), benzoic acid (TCI, >99%), 3-methylbenzoic acid (Sigma-Aldrich, 99%), 4-methylbenzoic acid (Sigma-Aldrich, 98%), 4-pentylbenzoic acid (Activate Scientific, 95%), naphthalene-2-carboxylic acid (Sigma-Aldrich, 98%), biphenyl-4-carboxylic acid (Sigma-Aldrich, 95%), methanol (MeOH, Scharlau, >99%). Molecules used as calibration standards were *N*,*N*-Dimethylformamide (DMF, Labkem, 99.9%), benzyl alcohol (BnOH, Sigma-Aldrich, ≥99%), dimethyl sulfoxide (DMSO, Labkem, HPLC), 1,3-dihydroxybenzene (resorcinol) (RESOR, Fluorochem, >99%) and tetramethylurea (TMU, Sigma-Aldrich, 99%). The drugs employed were 5-fluorouracil, (5-FU, TCI, >99%), gabapentin (GABA, TCI, >98%) and (R)-4-Aminoisoxazolidin-3-one (d-cycloserine) (CYCLO, BLDpharm, 98%). All the chemicals were used as commercially obtained.

### 4.2 Details of the Synthesis of All Compounds

#### 4.2.1 Synthesis of [Cu_7_(μ-adeninato)_6_(μ_3_-OH)_6_(μ-OH_2_)_6_]­(benzoate)_2_·∼17H_2_O (Cu_7_Bz) and [Cu_7_(μ-adeninato)_6_(μ_3_-OH)_6_(μ-OH_2_)_6_]­(benzoate)_2_·∼13H_2_O (**Cu**_**7**_ **Bz_D**)

The synthesis of the compound **Cu**_**7**_ **Bz** was carried out using a similar procedure previously reported but with slight variations.^32^ A mixture of 20 mL of methanol-water (1:1) containing 0.08 g of adenine (0.59 mmol), heated to 70 °C, was added to a solution of 0.13 g (0.54 mmol) of Cu­(NO_3_)_2_·3H_2_O in 10 mL of distilled water. The resulting mixture (pH ∼ 4) was stirred while NaOH was added dropwise until pH ∼ 9.3. The obtained mixture was added to 0.12 g of benzoic acid (0.98 mmol) dissolved in 20 mL of warm water basified with NaOH (pH ∼ 9.2). The final purple solution was covered with parafilm and allowed to stand at room temperature. After 3–4 days, blue cubic crystals of compound appeared. To accurately determine the pristine structure (**Cu**_**7**_ **Bz**), a suitable single crystal was extracted directly from the mother liquor and immediately measured via SC-XRD. The rest of the bulk product was isolated via vacuum filtration. As discussed below, this isolation process induces a partial dehydration of the framework with the code **Cu**_**7**_ **Bz_D**. Yield of the filtered crystals (**Cu**_**7**_ **Bz_D**): ca. 80% (based on copper). IR features (cm^–1^): 3465w, 3360vs, 3180s, 1640vs, 1610s, 1545s, 1500w, 1462s, 1395s, 1380vs, 1275s, 1200s, 1145s, 1025m, 935m, 790m, 725m, 665m, 550m, 460m.

#### 4.2.2 Synthesis of [Cu_7_(μ-adeninato)_6_(μ_3_-OH)_6_(μ-OH_2_)_6_]­(3-methylbenzoate)_2_·∼12H_2_O (Cu_7_3MBz) and [Cu_7_(μ-adeninato)_6_(μ_3_-OH)_6_(μ-OH_2_)_6_]­(3-methylbenzoate)_2_·∼11H_2_O (**Cu**_**7**_ **3MBz_D**)

Compound **Cu**_**7**_ **3MBz** was synthesized by the above-described method but using 0.14 g of 3-methylbenzoic acid (1.02 mmol). After 4–5 days, blue cubic crystals of compound **Cu**_**7**_ **3MBz** appeared. Following the same procedure as for the previous compound, a new partially dehydrated compound was obtained **Cu**_**7**_ **3MBz_D** with a yield: ca. 80% (based on copper). IR features (cm^–1^): 3460w, 3330s, 3170vs, 1640vs, 1600vs, 1540s, 1500w, 1460s, 1380vs, 1340vs, 1300s, 1270s, 1190vs, 1150s, 1030m, 980m, 930m, 790m, 740s, 650m, 560m, 450s.

#### 4.2.3 Synthesis of [Cu_7_(μ-adeninato)_6_(μ_3_-OH)_6_(μ-OH_2_)_6_]­(4-methylbenzoate)_2_·∼11H_2_O (Cu_7_4MBz) and [Cu_7_(μ-adeninato)_6_(μ_3_-OH)_6_(μ-OH_2_)_6_]­(4-methylbenzoate)_2_·∼10H_2_O (**Cu**_**7**_ **4MBz_D**)

The above-described procedure for compounds **Cu**_**7**_ **Bz** and **Cu**_**7**_ **3MBz** was used for the preparation of compound **Cu**_**7**_ **4MBz**, but employing 0.13 g of the 4-methylbenzoic acid (0.95 mmol). After 5-6 days, blue cubic single-crystals of **Cu**_**7**_ **4MBz** were obtained. The rest of the bulk product, **Cu**_**7**_ **4MBz_D** was isolated via vacuum filtration with a yield: ca. 80% (based on copper). Main IR features (cm^–1^): 3460w, 3360vs, 3180vs, 1640vs, 1600vs, 1540s, 1510w, 1460s, 1380vs, 1340vs, 1310s, 1280m, 1190vs, 1150s, 1010m, 970m, 930m, 770s, 740s, 650m, 550m, 450s.

#### 4.2.4 Synthesis of [Cu_7_(μ-adeninato)_6_(μ_3_-OH)_6_(μ-OH_2_)_6_]­(naphthalene-2-carboxylate)_2_·∼12H_2_O (Cu_7_2Naph) and [Cu_7_(μ-adeninato)_6_(μ_3_-OH)_6_(μ-OH_2_)_6_]­(naphthalene-2-carboxylate)_2_·∼11H_2_O (**Cu**_**7**_ **2Naph_D**)

Compound **Cu**_**7**_ **2Naph** was synthesized by the above-described method but using 0.16 g of naphthalene-2-carboxylic acid (0.93 mmol). After 4–5 days, blue cubic crystals of compound **Cu**_**7**_ **2Naph** appeared and measured by SC-XRD. The rest of the bulk product, **Cu**_**7**_ **2Naph_D** was isolated via vacuum filtration with a yield: ca. 75% (based on copper). IR features (cm^–1^): 3460w, 3320vs, 3180vs, 1630vs, 1600vs, 1540s, 1500m, 1460s, 1380s, 1340s, 1300m, 1280m, 1190vs, 1150s, 1020m, 970m, 940m, 790s, 740m, 650s, 550m, 450s.

#### 4.2.5 Synthesis of [Cu_7_(μ-adeninato)_6_(μ_3_-OH)_6_(μ-OH_2_)_6_]­(biphenyl-4-carboxylate)_2_·∼12H_2_O (Cu_7_4Bi) and [Cu_7_(μ-adeninato)_6_(μ_3_-OH)_6_(μ-OH_2_)_6_]­(biphenyl-4-carboxylate)_2_·∼9H_2_O (**Cu**_**7**_ **4Bi_D**)

Compound **Cu**_**7**_ **4Bi** was synthesized by the above-described method but using 0.11 g of biphenyl-4-carboxylic acid (0.55 mmol). After 4–5 days, blue cubic crystals of compound **Cu**_**7**_ **4Bi** appeared. The rest of the product, **Cu**_**7**_ **4Bi_D** was isolated via vacuum filtration with a yield: ca. 55% (based on copper). IR features (cm^–1^): 3460w, 3320vs, 3180vs, 1640vs, 1600s, 1540m, 1500w, 1470s, 1380vs, 1340s, 1300m, 1280m, 1190s, 1150m, 1020m, 980m, 930m, 790m, 750s, 660s, 550m, 450vs.

#### 4.2.6 Synthesis of [Cu_7_(μ-adeninato)_6_(μ_3_-OH)_6_(μ-OH_2_)_6_]­(4-pentylbenzoate)_2_·∼8H_2_O (**Cu**_**7**_ **4PBz**)

Compound **Cu**_**7**_ **4PBz** was synthesized by the above-described method but using 0.17 g of 4-pentylbenzoic acid (0.88 mmol). After 4–5 days, blue cubic crystals of compound **Cu**_**7**_ **4PBz** appeared. The rest of the product was isolated via vacuum filtration with a yield: ca. 70% (based on copper). IR features (cm^–1^): 3460w, 3330vs, 3140vs, 2930s, 1640vs, 1600vs, 1540s, 1500w, 1460s, 1380vs, 1350vs, 1300s, 1280s, 1190s, 1150s, 1020m, 970m, 930w, 780m, 740m, 650s, 550s, 450vs. Unfortunately, the crystals in the filtered sample are not of sufficient quality to achieve structural resolution by X-ray diffraction.

### 4.3 Characterization and Physical Methods

To check the purity of all the polycrystalline samples, routine PXRD measurements were carried out using a Philips X’Pert diffractometer (equipped with λCu_Kα_ = 1.5418 Å) over the range of 5° < 2θ to 70° with a step size of 0.02°, a variable automatic divergence slit, and an acquisition time of 2.5 s per step at 293 K (Figures S18 and S19). X-ray powder diffraction measurements were performed over filtered samples and over samples introduced in a Lindemann capillary containing the synthesis mother liquor. A Rigaku Smartlab automatic diffractometer with a capillary fixation head was employed to measure samples introduced in a Lindemann capillary containing the synthesis mother liquor. The diffraction data were collected in continuous rotation in the range 3° < 2θ < 65°. FTIR spectra of the samples (KBr pellets) were recorded at a resolution of 4 cm^–1^ in the 4000–400 cm^–1^ region using an FT/IR-8X Jasco spectrometer (Figure S20).

SC-XRD data for structure determination were collected with an Agilent Technologies Supernova diffractometer (λ_Mo_ _Kα_ = 0.71073 Å and λ_Cu Kα_ = 1.54184 Å). Data reduction was carried out with the CrysAlisPro program.^44^ The crystal structures were solved by direct methods using SIR92 ^45^ and refined by full-matrix least-squares on *F* ^2^, including all reflections with SHELXS,^46^ within the WINGX crystallographic software package.^47,48^ Several crystal structures exhibited disorder in adeninato ligands and the aromatic ring of the monocarboxylic ligands, Figure S1, also in some of the anions present in the voids, Figure S2. The disorder was modelled by distributing the disordered atoms over two positions and fixing the sum of their occupation factors to 1.0. Some of the highly disordered solvent molecules precluded their modeling, and consequently, the electron density was subtracted from the reflection data using the SQUEEZE method^49^ as implemented in PLATON.^50^ It is important to note that the accessible void percentages and free volumes reported throughout this work were calculated based on the host framework prior to the explicit assignment of these highly disordered solvent molecules, in order to accurately represent the total available space for host-guest interactions. The specific SQUEEZE electron counts, associated void volumes, and the corresponding assignment of disordered water molecules (assuming 10 electrons per H_2_O molecule) incorporated into the final chemical formulas are quantitatively detailed for each structure in the Supporting Information: Full crystallographic details are shown in Tables S1 and S2, Table S3 show all the data of SQUEEZE process.

Water vapor adsorption isotherms were obtained using a volumetric adsorption analyzer (3Flex, Micromeritics). Samples were outgassed under vacuum at 30 °C for 6 h prior to analysis. Measurements were performed at 293 K under vacuum using pure water vapor. Equilibrium was defined as a pressure change below 0.01% over ten times the equilibration interval (40–60 s).

Magnetic sustentation adsorption quantification was performed using a custom-built setup consisting of three magnetic stainless-steel pieces assembled into a U-shaped frame, with two circular permanent magnets magnetically attached to internal stainless-steel spacers at the upper ends.^40,41^ The frame was positioned within a methacrylate support base incorporating a linear actuator mounted between the magnets. A methacrylate plate fixed to the actuator shaft held a test tube containing the SMOF particles dispersed in liquid, allowing precise lateral displacement (0.01 mm resolution). The actuator was used to determine the critical distance (*D*) at which the particles detached from the magnet pole for unloaded and analyte-loaded samples, which was correlated with the percentage of adsorbed mass. All measurements were done in deionized water at room temperature. Each measurement was repeated 5 times to provide the corresponding associated error. The data to obtain the calibration curve were determined by high-performance liquid chromatography (HPLC) system model Shimadzu Nexera equipped with an SPD-20/40 V ultraviolet-visible (UV–vis) detector. The separation was performed using a Shim-pack VP-ODS column (4.6 mm × 250 mm, 5 μm particle size) maintained at 40 °C. The UV–vis detector wavelength and mobile phase composition were optimized for each analyte employed in the calibration curves. Resorcinol and benzyl alcohol were quantified at 254 nm, whereas DMSO, DMF, and TMU were detected at 220 nm. The mobile phase consisted of isocratic mixtures of water (phase A) and methanol (phase B), with volume ratios of 50:50 for resorcinol and TMU, 70:30 for benzyl alcohol, and 90:10 for DMSO and DMF. The flow rate was kept at 1 mL/min, with an injection volume of 60 μL. The calibration was carried out using all of these analytes solutions in the concentration range of 10–70 ppm.

### 4.4 Cytotoxicity Studies

Human colorectal cancer line *HCT116* (ATCC–CCL247) was employed. Cells were seeded at 3000 cells per well in 96 well plates and incubated at 37 °C and 5% CO_2_ in Dulbecco’s modified Eagle’s medium (DMEM) supplemented with 10% FBS and antibiotics. After overnight incubation the compounds were added to the cells and cells were collected for crystal violet staining at 24, 48, 72, and 120 h incubation time points. Subsequently, incubated cells were fixed with paraformaldehyde and stained with 0.1% crystal violet, Figure S17. Staining was washed 3 times; 10% acetic acid was added, and plates were incubated shaking for 20 min. Absorbance was measured at 590 nm and relative proliferation was calculated.

## Supplementary Material



## Notes

The data supporting this article have been included as part of the Supporting Information (crystallographic and structural data, porosity of the compounds, water adsorption isotherms, drugs loaded procedure and data, adsorptive molecules size and shape analysis, cytotoxicity, X-ray powder diffraction data, FTIR spectroscopic data, and experimental measurements of drug-loaded SMOFs) (PDF)
