# Supplementary material for: Pore Engineering with Benzoate Derivatives in Supramolecular Copper–Adenine Systems for Water and Drug Sorption
Source: Inorg Chem. 2026 Sep 23;65(39):23311–26. doi: 10.1021/acs.inorgchem.6c04013 (PMC13644310; doi:10.1021/acs.inorgchem.6c04013)
Supplement: Supplementary file 1 [file ic6c04013_si_001.pdf]

Supporting Information for

Pore Engineering with Benzoate Derivatives in Supramolecular Copper-Adenine Systems for Water and Drug Sorption.

Sandra Mena-Gutiérrez,[a] *Jon Pascual-Colino,*[a,b]* *Ainara Castellanos-Rubio,*[c,d,e] *Jon B. Etxebarria*[a] *and Antonio Luque*[a,f]

Corresponding author e-mail: jon.pascual@ehu.eus

[a] Departamento de Química Orgánica e Inorgánica, Facultad de Ciencia y Tecnología, Universidad del País Vasco/Euskal Herriko Unibertsitatea, EHU, E-48940 Leioa, Spain.

[b] Università degli studi di Milano, Dipartimento di Chimica, UNIMI, Via Golgi 19, Milano, 21033, Italy.

[c] IKERBASQUE, Basque Foundation for Science; E-48011, Bilbao, Spain.

[d] Biobizkaia Research Institute, E-480903 Barakaldo, Bizkaia, Spain.

[e] Departamento de Genética, Antropología Física y Fisiología Animal, EHU, E-48940 Leioa, Bizkaia, Spain.

[f] BCMaterials, Basque Center for Materials, Applications and Nanostructures, EHU Science Park, E-48940 Leioa, Spain

INDEX

S1. CRYSTAL STRUCTURE OF BENZOATE DERIVATE COMPOUNDS 3

S2. POROSITY OF THE COMPOUNDS 13

S3. WATER ADSORPTION PROCESSES 19

S4. DRUGS LOADED PROCEDURE AND DATA 21

S6. CYTOTOXICITY 28

S7. POWDER X-RAY DIFFRACTION 29

S8. INFRARED SPECTRA (FTIR) OF THE BENZOATE DERIVATE COMPOUNDS .. 31

S9. EXPERIMENTAL MEASUREMENTS OF DRUG LOADED SMOFs 32

S1. CRYSTAL STRUCTURE OF BENZOATE DERIVATE COMPOUNDS

SC-XRD data for all structures determination were collected with Agilent Technologies Supernova diffractometers with sources of $\lambda_{MoK\alpha}$= 0.71073 and $\lambda_{CuK\alpha}$= 1.54184 Å. Data reduction was performed with *CrysAlisPro* program (CrysAlisPRO, Oxford Diffraction; Agilent Technologies UK Ltd: Yarnton, England). The crystal structures were solved by direct methods using SIR92 (Altomare, A.; Cascarano, G.; Giacovazzo, C.; Guagliardi, A.; Burla, M. C.; Polidori, G.; Camalli, M. *J. Appl. Crystallogr.,* **1994**, *27*, 435–436.) and refined by full-matrix least-squares on F^2 including all reflections with SHELXS, (Sheldrick, G. M. *Acta Crystallogr. A Found Adv.*, **2008**, *64*, 112–122) within the WINGX crystallographic software package. (Farrugia, L. J. *J. Appl. Crystallogr.*, **2012**, *45*, 849–854). All the structures exhibited disorder in adeninato ligands and several ones also on the aromatic ring of the carboxylic ligands. All type of disorders were modelled by distributing the disordered atoms over two positions and constraining the sum of their occupation factors to one. Some of the highly disordered solvent molecules precluded their modelling, and consequently, the electron density was subtracted from the reflection data by the SQUEEZE method (Spek, A. L. *Acta Crystallogr. Sect. C*, **2015**, *C71*, 9–18) as implemented in PLATON. (Spek, A. L. *J. Appl. Crystallogr.*, **2003**, *36*, 7–13).

All non-hydrogen atoms were refined anisotropically, except those corresponding to disordered atoms. The hydrogen atoms belonging to organic entities have been geometrically fixed and refined according to a riding model with an isotropic thermal parameter linked to the atom to which they are attached (120%). In most cases, the hydrogen atoms of the ligands and anions have been located in the difference Fourier map, while for the coordination water molecules cases the routine CALC-OH (Nardelli, M. *Appl. Crystallogr.,* **1999***,* 32, 563–571) implemented in WINGX interface has been employed. The refinement of water hydrogen atoms has been performed using an isotropic thermal parameter of 150% regarding their parent atom. It has not been possible to position geometrically the hydrogen atoms of the crystallization water molecules due to the disorder that many of them present. During the structural solution of the compounds, it was common to observe the presence of a static disorder in the adenine molecules with an unusually high thermal motion of some atoms and nearby peaks in the Fourier map differences with high electron density were also observed. This disorder corresponds to the existence of two coplanar positions of the nucleobase with inverted orientation with respect to the coordination mode (μ-κ*N*3:κ*N*9/μ-κ*N*9:κ*N*3), as has been schemed in Figure S1. The disorder was modelled including the peaks observed as atoms split in two positions (A and B), to which common free occupancy factors were assigned for each of the subgroups with the condition that the two occupancy factors add up to 100%.

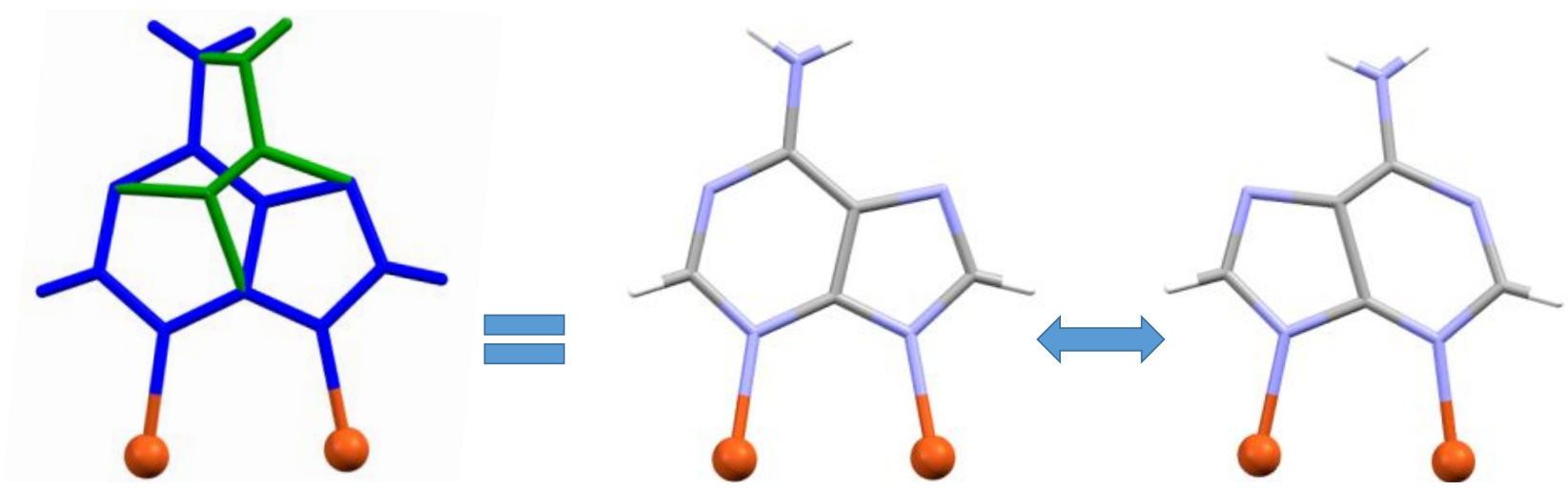

Figure S1. Disorder of the adeninato ligand with two coplanar orientations colored in blue and green.

In the cases of the **$Cu_7$3MBz**, **$Cu_7$4Bi** and **$Cu_7$2Naph** compounds and their derivatives, a non-coplanar disorder has been modeled for the aromatic ring of the benzoate derivate, Figure S2. In the case of the naphthalene-2-carboxylate and biphenyl-4-carboxylate anions, the disorder is place in the further aromatic ring from the carboxylic group. The one that is not as well attached to the cluster by π–π stacking as the prior one and is placed in the hexagonal voids of each structure.

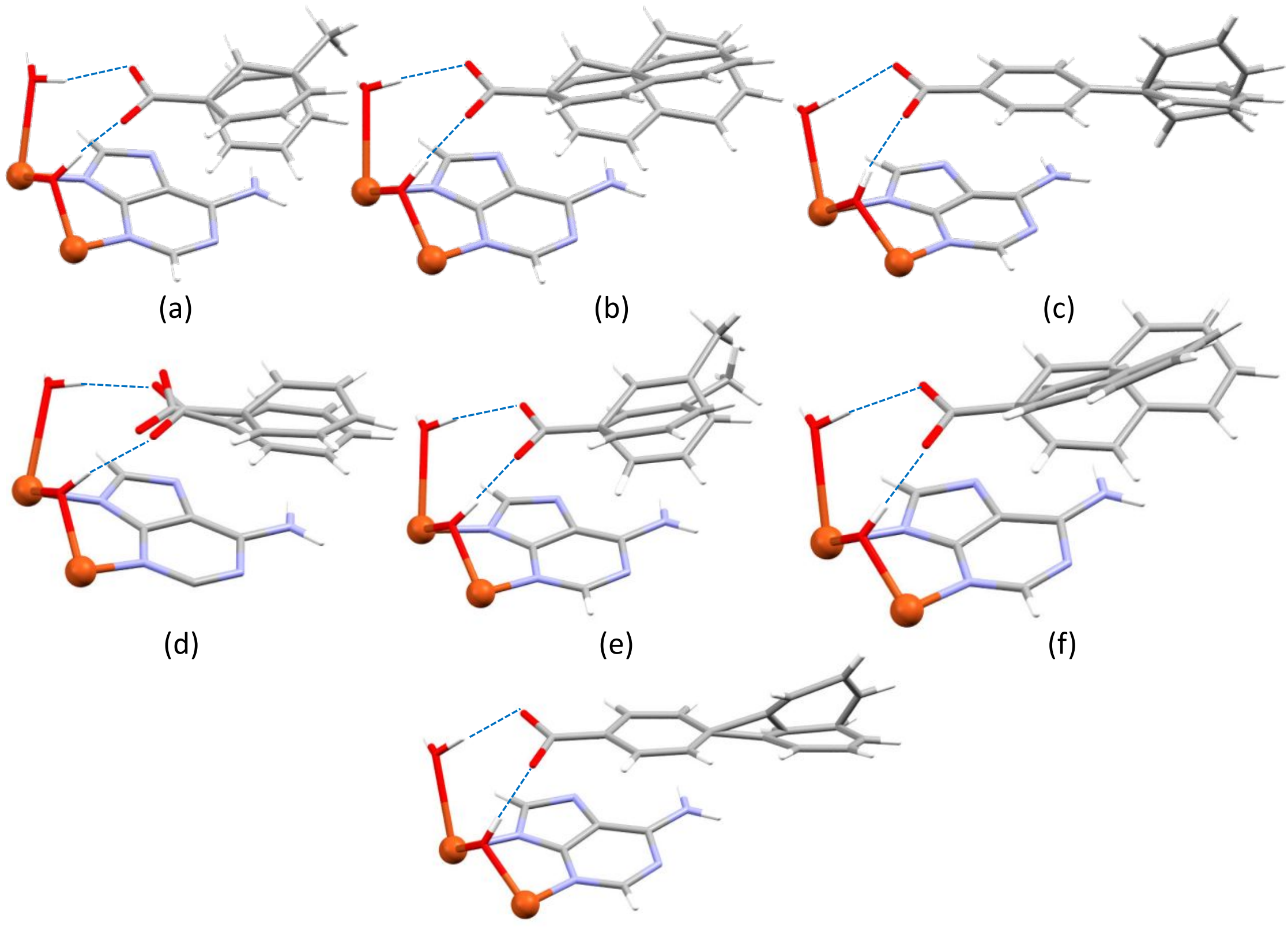

Figure S2. Disorder of the aromatic ring of the benzoate derivate anions in (a) **$Cu_7$3MBz**, (b) **$Cu_7$2Naph** and (c) **$Cu_7$4Bi**, (d) **Cu_7Bz_D**, (e) **$Cu_7$3MBz_D**, (f) **$Cu_7$2Naph_D** and (g) **$Cu_7$4Bi_D** compounds respectively.

The resulting supramolecular structures exhibit elevated levels of porosity. In this study, we present three novel structures exhibiting a substantial change on the free porous space, with values ranging from 29% to 12%. The water content of these structures has been determined as follows. Apart from the located water molecules during the structure resolution, the electronic density subtracted by the SQUEEZE procedure was assumed to correspond to disordered water molecules that has been impossible to localize, assigning the value of ten electrons for each water molecule.

The porosity percentage was determined by initially resolving the crystal structure of the central cluster and the carboxylic anion, leaving the voids free, and then assigning the high electron density regions as water molecules. The representation of the crystal structures has been made by MERCURY (MacRae, C. F.; Sovago, I.; Cottrell, S. J.; et al. *J. Appl. Crystallogr.,* **2020***, 53*, 226–235.). Full crystallographic details are provided in Table S1 for samples measured in their mother liquid and Table S2 for filtered and dry samples.

Table S1. Crystallographic data and structure refinement details of all compounds.[a,b,c]

	**$Cu_7$3MBz**	**$Cu_7$4MBz**	**$Cu_7$2Naph**	**$Cu_7$4Bi**	**$Cu_7$4PBz**
Empirical formula	$C_{46}H_{80}Cu_7N_{30}O_{28}$	$C_{46}H_{78}Cu_7N_{30}O_{27}$	$C_{52}H_{80}Cu_7N_{30}O_{28}$	$C_{56}H_{84}Cu_7N_{30}O_{28}$	$C_{54}H_{88}Cu_7N_{30}O_{24}$
Formula weight	1946.18	1928.16	2018.24	2070.31	1986.32
Crystal system	Trigonal	Trigonal	Trigonal	Trigonal	Trigonal
Space group	$R\overline{3}$ (148)	$R\overline{3}$ (148)	$R\overline{3}$ (148)	$R\overline{3}$ (148)	$R\overline{3}$ (148)
a	30.9041(7)	30.7143(6)	31.4361(7)	31.5881(7)	31.0890(5)
b	30.9041(7)	30.7143(6)	31.4361(7)	31.5881(7)	31.0890(6)
c	20.8746(4)	20.8768(2)	20.8606(3)	20.7720(4)	20.7700(4)
α	90	90	90	90	90
β	90	90	90	90	90
γ	120	120	120	120	120
V (Å^3)	17265.6(10)	17055.9(7)	17853.1(8)	17949.6(9)	17385.2(7)
Z	9	9	9	9	9
T (K)	170.0(1)	170.0(1)	170.0(1)	170.0(1)	150.0(1)
λ (Å)	0.71073	1.54184	0.71073	0.71073	1.54184
Size (mm)	0.06/0.06/0.02	0.06/0.06/0.03	0.05/0.04/0.03	0.08/0.06/0.04	0.05/0.04/0.04
Shape	Cubic	Cubic	Cubic	Cubic	Cube
Color	Blue	Blue	Blue	Blue	Blue
Max. and medium Δ/σ	0.000 / 0.000	0.000 / 0.000	0.000 / 0.000	0.000 / 0.000	0.000 / 0.000
θ interval	2.093 – 26.084	2.099 – 26.860	2.092 – 26.000	2.103 – 24.400	3.911 – 62.500
hkl interval	–43≤h≤42<br>–42≤k≤41<br>–28≤l≤27	–39≤h≤42<br>–41≤k≤42<br>–25≤l≤27	–38≤h≤38<br>–38≤k≤37<br>–25≤l≤25	–36≤h≤36<br>–36≤k≤36<br>–24≤l≤24	–35≤h≤30<br>–22≤k≤35<br>–23≤l≤21
ρ_c (g·cm^{-3})	1.685	1.690	1.689	1.724	1.707
μ (cm^{-1})	1.998	2.021	1.936	1.928	2.883
F(000)	8937	8847	9261	9513	9153
S[a]	1.081	1.064	1.052	1.096	1.200
R_{int}	0.0784	0.0568	0.0413	0.0511	0.0248
Parameters	432	478	448	499	469
Weight scheme[c]	Shelx	Shelx	Shelx	Shelx	Shelx
final R indices					
[I > 2σ(I)] $R_1{}^b/wR_2{}^c$	0.1256/0.3225	0.0873/0.2168	0.0903/0.2708	0.0982/0.2435	0.0729/0.2214
all data $R_1{}^b/wR_2{}^c$	0.2022/0.3627	0.1360/0.2412	0.1225/0.2962	0.1144/0.2521	0.0776/0.2266

[a]$S=[\sum w(F_0^2-F_c^2)^2/(N_{obs}-N_{param})]^{1/2}$. [b]$R_1=\sum||F_0|-|F_c||/\sum|F_0|$. [c]$wR_2 = [\sum w(F_0^2-F_c^2)^2/\sum wF_0^2]^{1/2}$; $w = 1/[\sigma^2(F_0^2) + (aP)^2 + b]$ where $P = (\max(F_0^2,0) + 2F_c^2)/3$; **$Cu_7$3Met** (a = 0.1481, b = 317.3225); **$Cu_7$4Met** (a = 0.1108, b = 116.9524), **$Cu_7$2Naph** (a = 0.1685, b = 110.593), **$Cu_7$4Bi** (a = 0.0785, b = 558.5591) and **$Cu_7$4PBz** (a = 0.1212, b = 137.8504).

Table S2. Crystallographic data and structure refinement details of all filtered compounds.[a,b,c]

	Cu_7Bz_D	Cu_73MBz_D	Cu_74MBz_D	Cu_72Naph_D	Cu_74Bi_D
Empirical formula	$C_{44}H_{78}Cu_7N_{30}O_{29}$	$C_{46}H_{78}Cu_7N_{30}O_{27}$	$C_{46}H_{76}Cu_7N_{30}O_{26}$	$C_{52}H_{78}Cu_7N_{30}O_{27}$	$C_{56}H_{78}Cu_7N_{30}O_{25}$
Formula weight	1936.14	1928.16	1910.14	2000.22	2016.26
Crystal system	Trigonal	Trigonal	Trigonal	Trigonal	Trigonal
Space group	$R\bar{3}$ (148)	$R\bar{3}$ (148)	$R\bar{3}$ (148)	$R\bar{3}$ (148)	$R\bar{3}$ (148)
a	30.4500(3)	30.7245(10)	30.4929(7)	30.9597(10)	31.2649(5)
b	30.4500(3)	30.7245(10)	30.4929(7)	30.9597(10)	31.2649(5)
c	21.0004 (2)	20.8630(5)	20.8902(4)	20.8368(5)	20.7654(3)
α	90	90	90	90	90
β	90	90	90	90	90
γ	120	120	120	120	120
V (Å^3)	16862.9(4)	17056.0(1)	16821.7(8)	17296.4(1)	17578.6(6)
Z	9	9	9	9	9
T (K)	150.0(1)	170.0(1)	170.0(1)	150.0(1)	150.0(1)
λ (Å)	1.54184	0.71073	0.71073	1.54184	1.54184
Size (mm)	0.07/0.06/0.06	0.10/0.08/0.06	0.10/0.09/0.07	0.03/0.02/0.02	0.07/0.06/0.04
Shape	Cubic	Cubic	Cubic	Cubic	Cube
Color	Blue	Blue	Blue	Blue	Blue
Max. and medium Δ/σ	0.000 / 0.000	0.000 / 0.001	0.000 / 0.000	0.000 / 0.000	0.000 / 0.000
θ interval	2.687 – 62.500	2.096 – 24.500	2.099 – 24.200	2.679 – 62.500	4.5430 – 62.500
hkl interval	$-34\leq h\leq 35$ $-35\leq k\leq 22$ $-24\leq l\leq 24$	$-35\leq h\leq 35$ $-35\leq k\leq 35$ $-24\leq l\leq 24$	$-35\leq h\leq 34$ $-35\leq k\leq 35$ $-24\leq l\leq 24$	$-35\leq h\leq 35$ $-34\leq k\leq 32$ $-23\leq l\leq 23$	$-35\leq h\leq 35$ $-35\leq k\leq 32$ $-18\leq l\leq 23$
ρ_c (g·cm^{-3})	1.716	1.689	1.697	1.728	1.714
μ (cm^{-1})	3.005	2.021	2.047	2.935	2.876
F(000)	8883	8847	8757	9171	9243
S^a	1.061	1.039	1.036	1.016	1.059
R_{int}	0.0226	0.0803	0.0363	0.0552	0.0395
Parameters	457	421	480	460	488
Weight scheme^c	Shelx	Shelx	Shelx	Shelx	Shelx
final R indices					
[I > 2σ(I)] R_1^b/wR_2^c	0.0684/0.2132	0.0919/0.2499	0.0648/0.1722	0.0878/0.2470	0.0870/0.2534
all data R_1^b/wR_2^c	0.0743/0.2184	0.1278/0.2749	0.0872/0.1867	0.1299/0.2812	0.1028/0.2676

$^a S=[\sum w(F_0^2-F_c^2)^2/(N_{obs}-N_{param})]^{1/2}$. $^b R_1=\sum\|F_0|-|F_c\|/\sum|F_0|$. $^c wR_2 = [\sum w(F_0^2-F_c^2)^2/\sum wF_0^2]^{1/2}$; $w = 1/[\sigma^2(F_0^2) + (aP)^2 + b]$ where $P = (\max(F_0^2,0) + 2F_c^2)/3$; **$Cu_7Bz\_D$** (a = 0.1362, b = 108.2938); **Cu_73Met_D** (a = 0.1415, b = 215.9794); **Cu_74Met_D** (a = 0.1019, b = 144.1479), **Cu_72Naph_D** (a = 0.1639, b = 103.9145) and **Cu_74Bi_D** (a = 0.1483, b = 187.2668).

Table S3. Details of the SQUEEZE procedure and assignment of disordered solvent molecules. Voids with values below 40 A^3 and 10 electrons have not taken into account due to this tiny space.

Compound	Void Vol. (A^3)	e^- count	Disordered H_2O molec (e^-/10)	Final reported H_2O in formula
**Cu_7Bz**	1954	575	57.5 per cell/ 6.4 per formula	17
**Cu_7Bz_D**	1381	300	30 per cell/ 3.3 per formula	13
**Cu_73MBz**	2125	576	57.6 per cell/ 6.4 per formula	12
**Cu_73MBz_D**	1182	348	34.8 per cell/ 3.9 per formula	11
**Cu_74MBz**	1223	99	9.9 per cell/ 1.1 per formula	11
**Cu_74MBz_D**	1190	76	7.6 per cell/ 0.8 per formula	10
**Cu_72Naph**	966	261	26.1 per cell/ 2.9 per formula	12
**Cu_72Naph_D**	521	147	14.7 per cell/ 1.6 per formula	11
**Cu_74Bi**	1470	411	41.1 per cell/ 4.6 per formula	12
**Cu_74Bi_D**	526	84	8.4 per cell/ 0.9 per formula	9
**Cu_74PBz**	NO	NO	NO	8

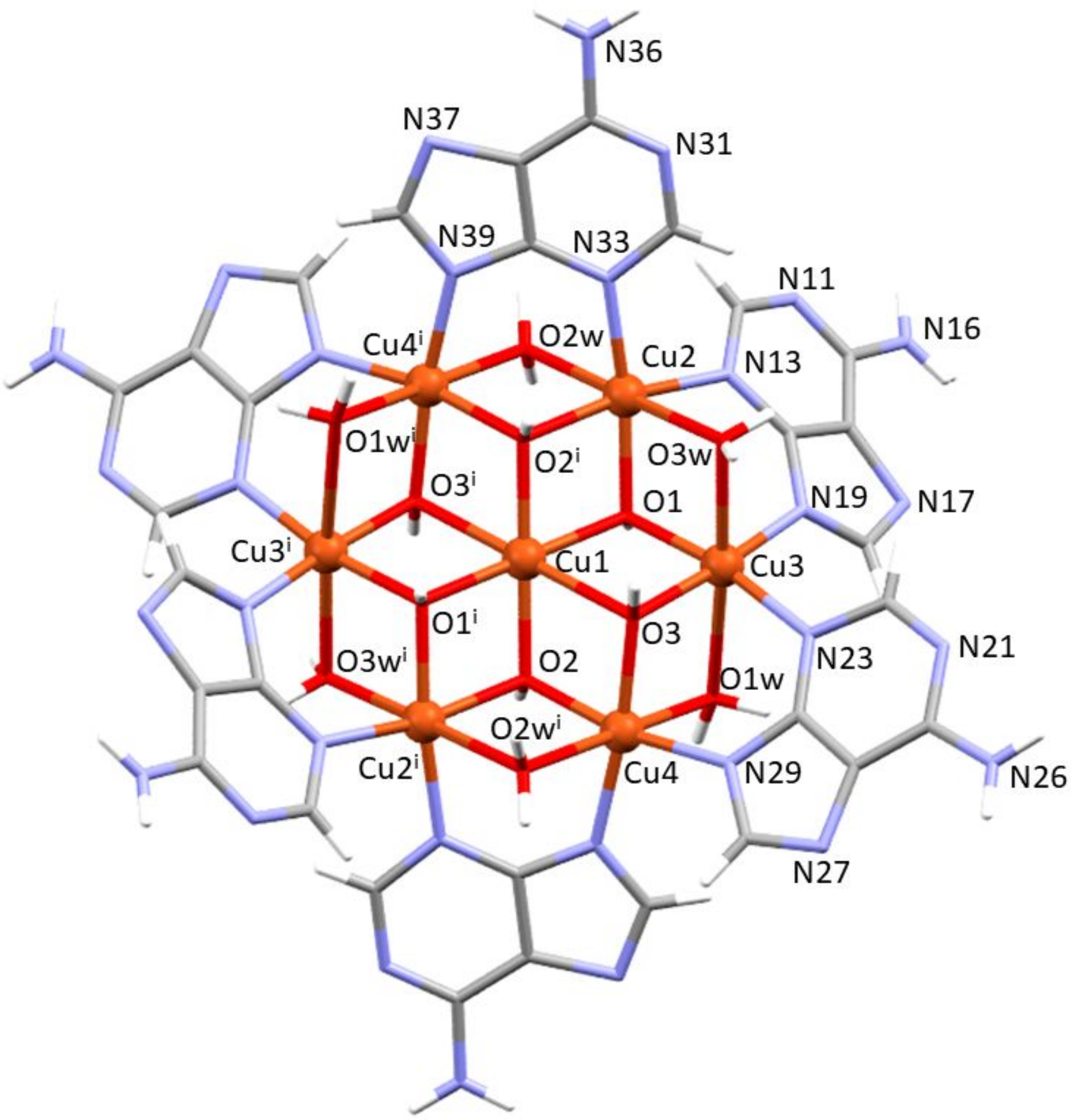

(a)

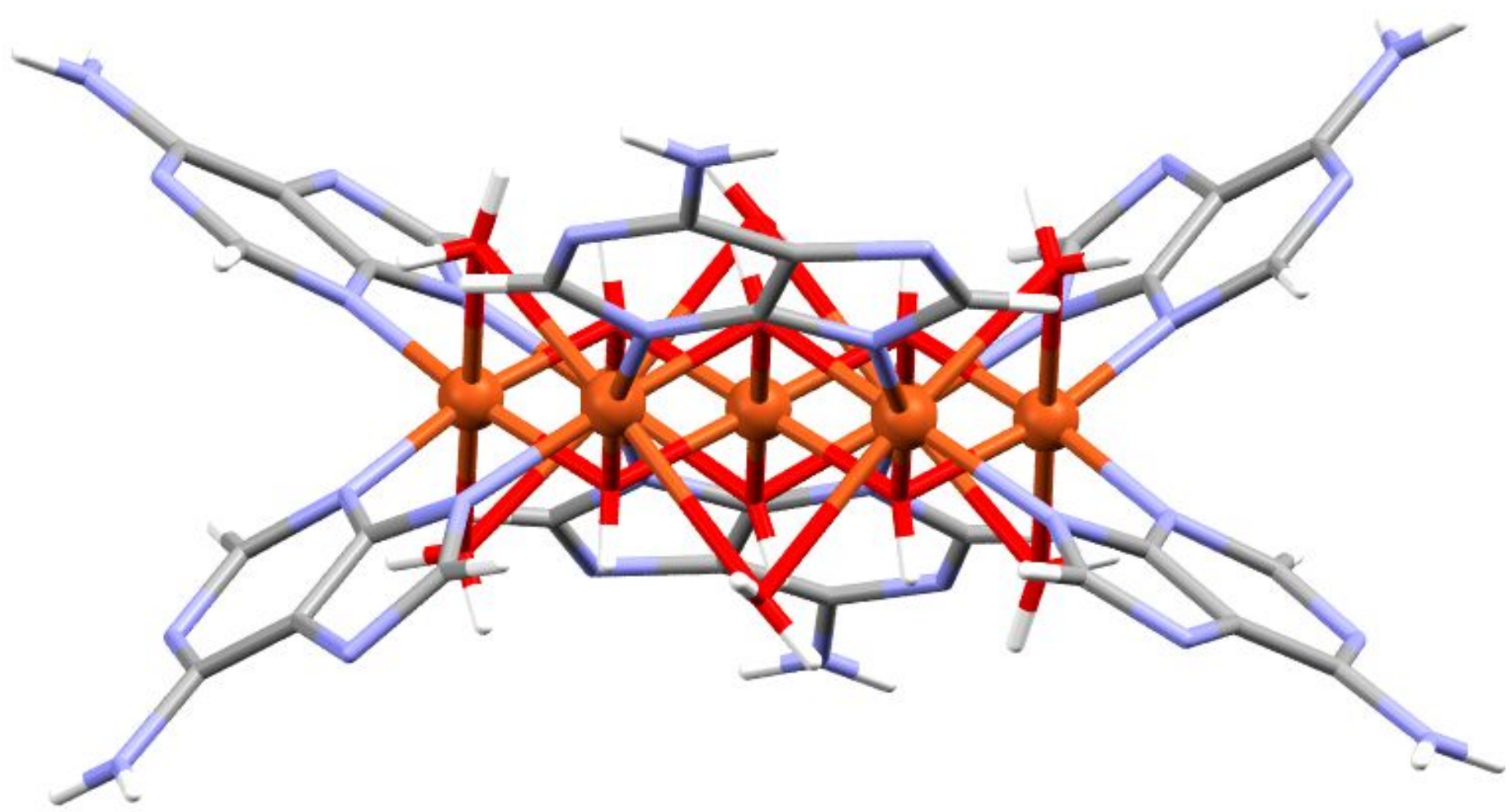

(b)

Figure S3. Heptameric copper cluster front view showing the numbering scheme use in the article. For the sake of clarity, only the adeninato ligands of the asymmetric unity are labeled.

Table S4. Structural parameters (Å, deg) of hydrogen-bonding interactions in **Cu_7Bz** derivatives measured in their mother liquid.[a]

**Hydrogen-bonding interactions**				
Compound	D–H···A[b]	H···A	D···A	D–H···A
**$Cu_7$3MBz**	O1–H1···O4W	1.82	2.67(1)	170
	O3–H3···O48^{i}	1.63	2.61(2)	166
	O1w–H12w···O49	1.85	2.76(2)	173
	O2w–H22w···O49^{i}	1.94	2.79(1)	171
	O3w–H31w···O6W	2.08	2.92(2)	172
**$Cu_7$4MBz**	O2–H2···O4W	1.85	2.69(7)	170
	O3–H3···O48	1.85	2.70(7)	173
	O1w–H12w···O49^{ii}	1.92	2.78(8)	172
	O2w–H21w···O49^{iii}	1.95	2.79(1)	170
	O2w–H22w···O48	2.21	3.07(1)	167
	O3w–H32w···O8W^{iv}	2.04	2.90(3)	178
	N16–H16A···O7W	2.01	2.79(2)	148
**$Cu_7$2Naph**	O1–H1···O7W^{v}	2.29	3.13(2)	163
	O2–H2···O8W	1.79	2.65(8)	172
	O3–H3···O49	1.72	2.71(1)	169
	O1w–H11w···O48	1.92	2.77(1)	173
	O2w–H21w···O7W^{v}	1.90	2.68(2)	161
	O2w–H22w···O48^{vi}	1.92	2.80(1)	173
	O3w–H31w···O6W^{vii}	2.08	2.95(2)	169
**$Cu_7$4Bi**	O1–H1···O48	1.83	2.69(1)	169
	O2–H2···O5W	2.17	3.04(3)	172
	O3–H3···O6W	1.80	2.65(1)	167
	O1w–H11w···O4W	2.07	2.92(2)	163
	O1w–H12w···O6W	2.17	3.02(1)	155
	O2w–H21w···O49	1.90	2.75(1)	174
	O3w–H31w···O49^{ix}	1.98	2.82(2)	171
	O3w–H32w···O5W	1.80	2.67(4)	168
**$Cu_7$4PBz**	O1–H1···O7W	1.80	2.66(7)	172
	O2–H2···O48	1.81	2.66(7)	175
	O1w–H11w···O49^{x}	1.99	2.84 (1)	175
	O1w–H12w···O48	2.21	3.05(1)	166
	O2w–H21w···O4w	1.99	2.81(2)	162
	O2w–H22w···O7w	2.05	2.90(6)	169
	O3w –H31w···O49	1.92	2.76(7)	173

[a]Symmetry codes: (i) 2/3 – x + y, 1/3 – x – y, 1/3 + z; (ii) 1 – x, – y, 2 – z; (iii) 1/3 + x – y, – 2/3 + x, 7/3 – z; (iv) 1 – x + y, – x, z; (v) 1 – x, – y, – z; (vi) 1/3 – y, – 1/3 + x – y, – 1/3 + z; (vii) 2/3 – y, – 2/3 + x – y, 1/3 + z; (ix) 2/3 – x + y, 1/3 – x, 1/3 + z and (x) 1/3 + x – y,– 2/3 + x, 1/3 – z. [b]D: donor; A: acceptor.

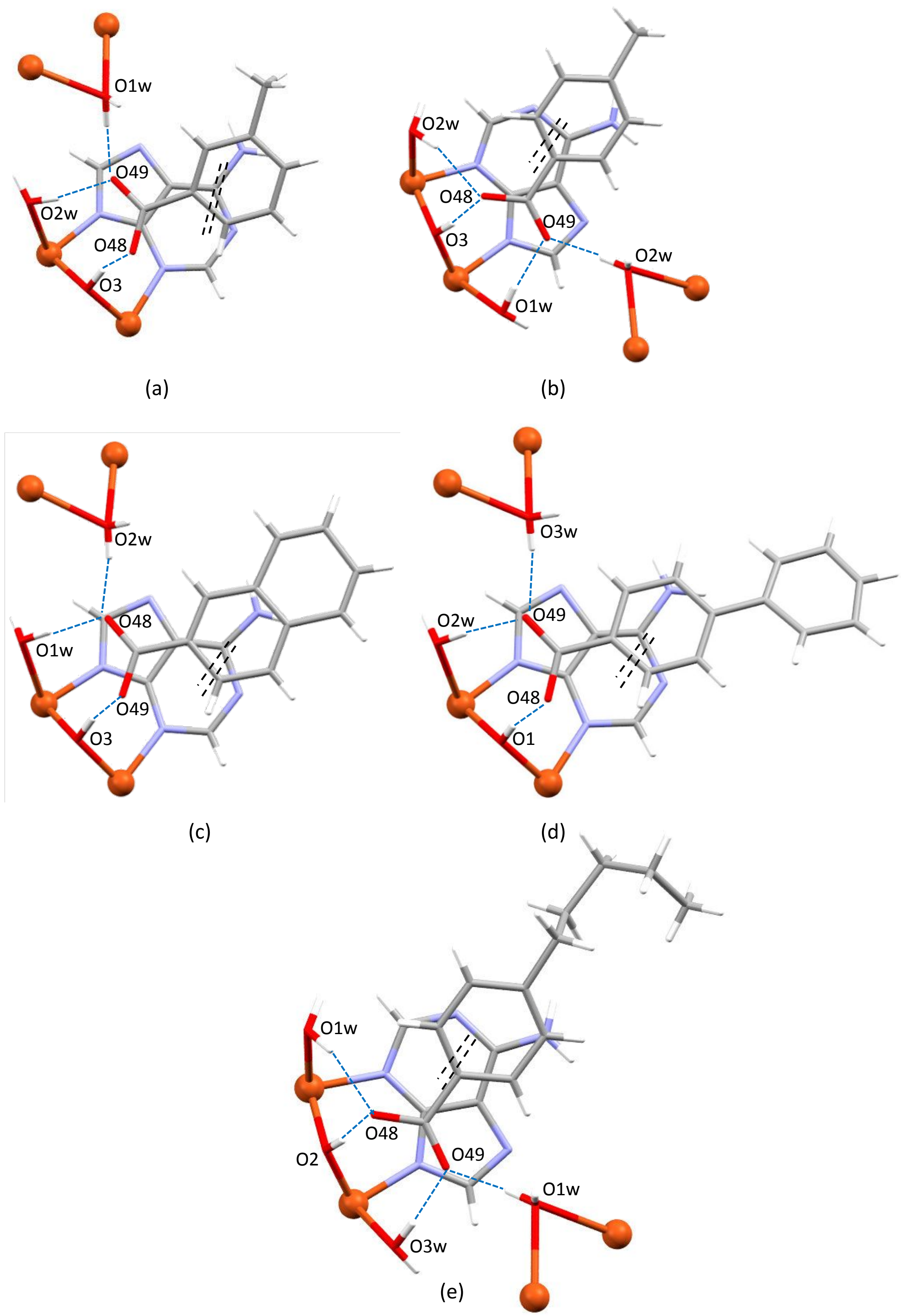

Figure S4. Details of the supramolecular interactions between the organic anions and the cationic complexes in compounds: (a) **$Cu_7$3MBz**, (b) **$Cu_7$4MBz**, (c) **$Cu_7$2Naph**, (d) **$Cu_7$4Bi** and (e) **$Cu_7$4PBz**.

Table S5. Structural parameters (Å, deg) of hydrogen-bonding interactions in **Cu_7Bz** derivates measured after filtered and removed from their mother liquid.[a]

**Hydrogen-bonding interactions**				
Compound	D–H···A[b]	H···A	D···A	D–H···A
**Cu_7Bz_D**	O1–H1···O8W	2.27	3.12(2)	170
	O2–H2···O48A	1.90	2.75(1)	172
	O3–H3···O4w	1.88	2.73(7)	170
	O1w–H11w···O49A	1.84	2.68(1)	172
	O2w–H21w···O7w	2.08	2.93(1)	172
	O2w–H22w···O8w	1.99	2.83(2)	173
	O3w–H31w···O48A	2.23	3.05(1)	163
	O3w–H32w···O49A^{i}	2.05	2.90(2)	172
	N16–H16A···O6W	2.12	2.92(1)	152
**$Cu_7$3MBz_D**	O1–H1···O48	1.76	2.61(2)	167
	O3–H3···O4W	1.81	2.66(1)	171
	O1w–H11w···O6w	2.13	2.75(1)	167
	O2w–H21w···O49	1.91	2.79(1)	169
	O3w–H31w···O49^{ii}	1.90	2.75(2)	173
	O3w–H32w···O48^{iii}	2.35	3.17(2)	161
	N16–H16A···O7B^{iv}	2.19	2.97(4)	148
**$Cu_7$4MBz_D**	O2–H2···O48	1.83	2.69(7)	173
	O3–H3···O4W	1.85	2.71(7)	174
	O1w–H11w···O49	1.93	2.78(8)	173
	O2w–H21w···O4w	2.06	2.91(9)	175
	O2w–H22w···O5w	2.03	2.87(2)	170
	O3w–H31w···O49^{v}	1.95	2.80(9)	169
	O3w–H32w···O48	2.10	2.97(9)	167
	N16–H16A···O7W^{vi}	2.19	3.06(2)	172
**$Cu_7$2Naph_D**	O1–H1···O48	1.83	2.68(1)	171
	O2–H2···O4W	1.78	2.64(1)	176
	O3–H3···O8W	2.25	3.10(2)	168
	O1w–H12w···O6W	2.17	3.02(2)	169
	O2w–H21w···O49^{vii}	1.95	2.80(2)	165
	O2w–H22w···O8w	1.80	2.64(2)	164
	O3w–H32w···O49	1.89	2.74(1)	172
	N36–H36B···O7W^{i}	2.07	2.87(3)	151
**$Cu_7$4Bi_D**	O2–H2···O4W^{iii}	1.82	2.66(8)	171
	O3–H3···O49	1.84	2.69(8)	173
	O1w–H12w···O48	1.88	2.73(1)	170
	O2w–H12w···O48^{viii}	2.01	2.86(1)	175
	O2w–H11w···O49^{iii}	2.32	3.15(1)	160
	O3w–H31w···O5w	2.10	2.90(2)	156
	O3w –H32w···O4w	2.08	2.95(9)	160
	N16A–H16A···O7W^{ix}	2.07	2.93(3)	164

[a]Symmetry codes: (i) 2/3 + y, 1/3 – x, 1/3 – z; (ii) 1/3 – y, – 1/3 + x – y, – 1/3 + z; (iii) 1 − x, − y, 1 − z; (iv) 1/3 + y, 2/3 – x + y, 5/3 – z; (v) 2/3 + y, 1/3 – x + y, 1/3 – z; (vi) 1/3 + y, 2/3 – x + y, 2/3 – z; (vii) 2/3 + y, 1/3 – x + y, 4/3 – z; (viii) 2/3 – x + y, 1/3 – x, 1/3 + z; and (ix) 1/3 + x − y,– 2/3 + x, – 2/3 – z. [b]D: donor; A: acceptor.

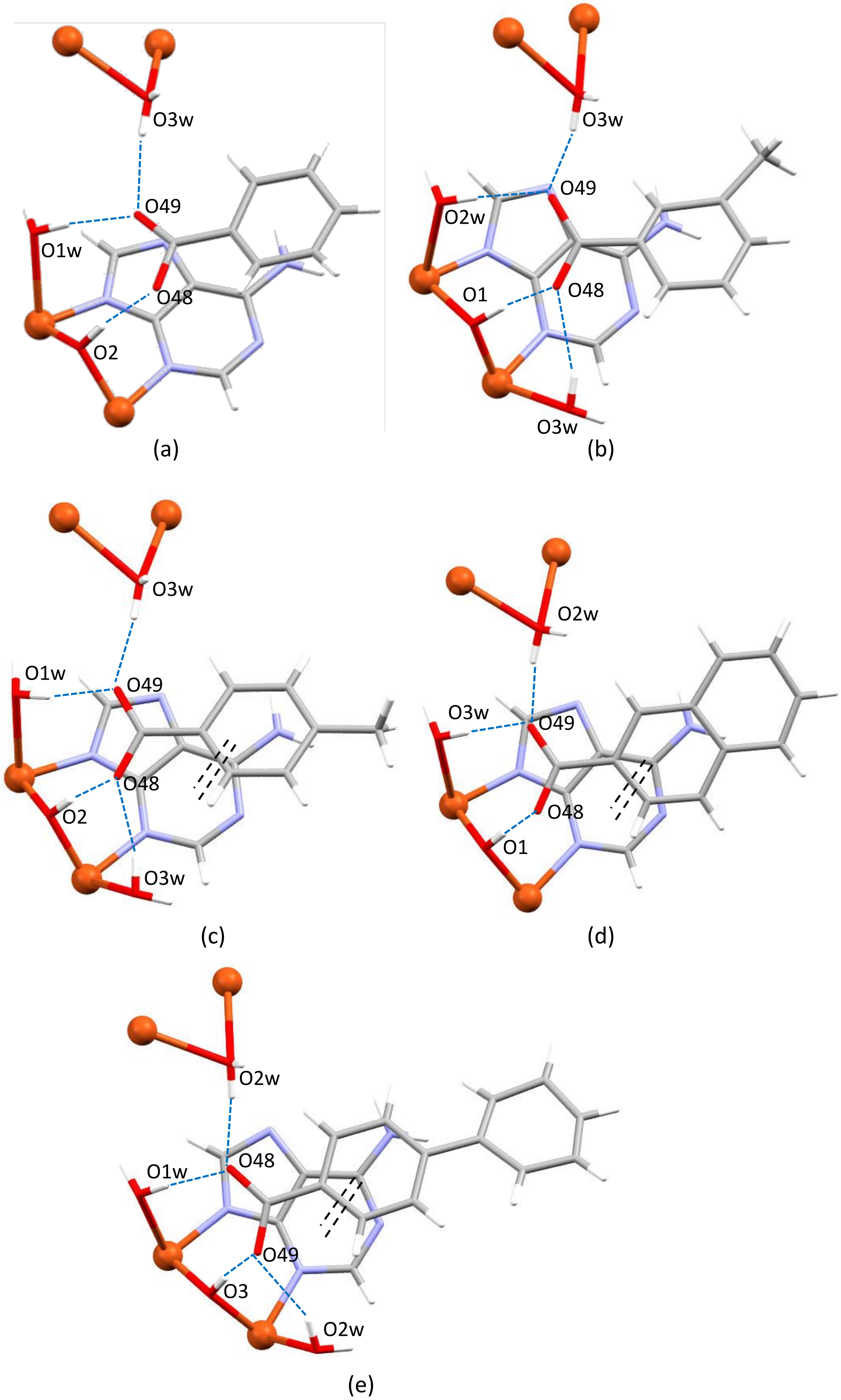

Figure S5. Details of the supramolecular interactions between the organic anions and the cationic complexes in compounds: (a) **Cu_7Bz_D** (b) **$Cu_7$3MBz_D**, (c) **$Cu_7$4MBz_D**, (d) **$Cu_7$2Naph_D** and (e) **$Cu_7$4Bi_D**.

Table S6. Structural parameters (Å) of π–π stacking interactions between the cluster adeninato ligand and the benzoate derivate anion in all the compounds measured in their mother liquid ordered by π–π stacking interaction strength.

**π–π interactions**						
**Compound**	ring–ring[a]	packing[b]	angle[c]	DC	DZ	DXY
**$Cu_7$3MBz**	3pa···4hm[i]	A···D	0	5.09	3.61	3.21
**Cu_7Bz**	1pa···4hm	A···D	2.7	4.64	3.66	2.81
**$Cu_7$4Bi**	1pa···4hm	A···D	7.0	4.58	3.67	2.63
**$Cu_7$2Naph**	3pa···4hm	A···D	12.0	4.77	3.71	2.62
**$Cu_7$4MBz**	3pa···4hm	A···D	5.7	5.06	3.76	3.23
**$Cu_7$4PBz**	2pa···4hm	A···D	10.2	4.97	3.66	3.62

[a]Symmetry codes: (i) 2/3 − x + y, 1/3 − x, 1/3 + z; [b]D: derivate; A: adeninato. [c]Angle: dihedral angle between the planes (deg), DC: distance between the centroids of the rings (Å), DZ: interplanar distance (Å), DXY: lateral displacement (Å), pa: adenine pentagonal ring, ha adenine hexagonal ring, hm monocarboxylic hexagonal ring.

Table S7. Structural parameters (Å) of π–π stacking interactions between the cluster adeninato ligand and the benzoate derivate anion in all the compounds measured after filtration ordered by π–π stacking interaction strength.

**π–π interactions**						
**Compound**	ring–ring[a]	packing[b]	angle[c]	DC	DZ	DXY
**$Cu_7$4MBz_D**	2pa···4hm	A···D	6.0	5.09	3.74	3.29
**$Cu_7$4Bi_D**	3pa···4hm[i]	A···D	11.2	4.65	3.55	2.87
**$Cu_7$2Naph_D**	1pa···4hm	A···D	12.6	4.87	3.82	2.77

[a]Symmetry codes: (i) 1 − x, − y, − z; [b]D: derivate; A: adeninato. [c]Angle: dihedral angle between the planes (deg), DC: distance between the centroids of the rings (Å), DZ: interplanar distance (Å), DXY: lateral displacement (Å), pa: adenine pentagonal ring, ha adenine hexagonal ring, hm monocarboxylic hexagonal ring.

S2. POROSITY OF THE COMPOUNDS

The data of the crystal structure (atoms distance/angles and hydrogen bond distances) of the **Cu_7Bz** compound is described in a previous paper published by our group. (Pascual-Colino, J.; Beobide, G.; Castillo, O.; Lodewyckx, P.; Luque, A.; Pérez-Yáñez, S.; Román P.; Velasco, L. F. *J. Inorg. Biochem.* **2020**, *202*, 110865).

To assess the suitability of **Cu_7Bz** and their variants potential voids for the selected drugs and water molecules, we have analyzed their pore features (Table S8). Their geometric pore size distribution (PSD) was computed by means of a Monte Carlo procedure implemented within a code developed by L. Sarkisov, in which the Lennard-Jones (LJ) universal force field parameters. It is used to describe the SMOF atoms while the accessible pore volume is assessed by a gradually increasing probe size (Sarkisov, L.; Bueno, R.; Sutharson, M. et al. *Chem. Mater*., **2020**, *32*, 9849–9867).

The results are in Figure S6. Porosity and surface area values were estimated via Monte Carlo simulations, as standard BET gas adsorption measurements could not be performed; the degassing process required to evacuate water molecules, even when applying extremely mild outgassing conditions (using very low temperatures and carefully controlled vacuum), induces the collapse and amorphization of the framework's surface, preventing gas accessibility. For all this calculations original CIFs without the crystallization water molecules and disorder have been used.

Table S8. Porosity data for the reported compounds.

Compound	d_{Pore} (mode, Å)		Void volume (Å^3)	Void (%)	Surface area (m^2/g)	Pore volume (cm^3/g)
	min	max				
**Cu_7Bz**	4.00	6.53	5098	29	205	0.200
**$Cu_7$3MBz**	2.43	6.48	3602	21	85	0.139
**$Cu_7$4MBz**	1.84	6.00	3234	19	62	0.125
**$Cu_7$2Naph**	2.68	6.78	3633	20	77	0.135
**$Cu_7$4Bi**	3.18	6.37	3194	18	56	0.115
**$Cu_7$4PBz**	1.89	5.34	2054	12	>20	0.075

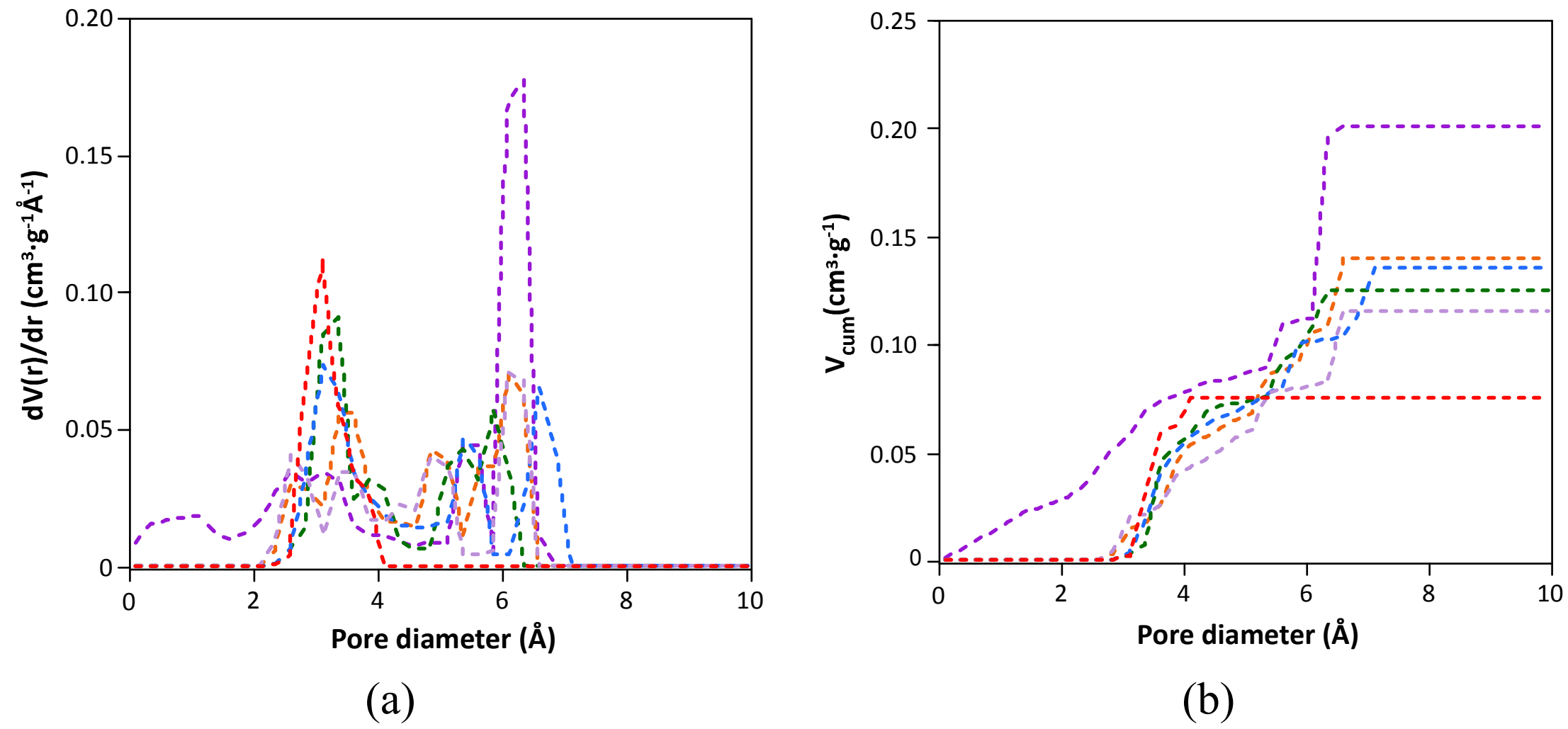

Figure S6. (a) Derivative and (b) cumulative representation of the geometric pore volume of **$Cu_7$3MBz** (orange), **$Cu_7$4MBz** (green), **$Cu_7$2Naph** (blue), **$Cu_7$4Bi** (pink) and **$Cu_7$4PBz** (red).

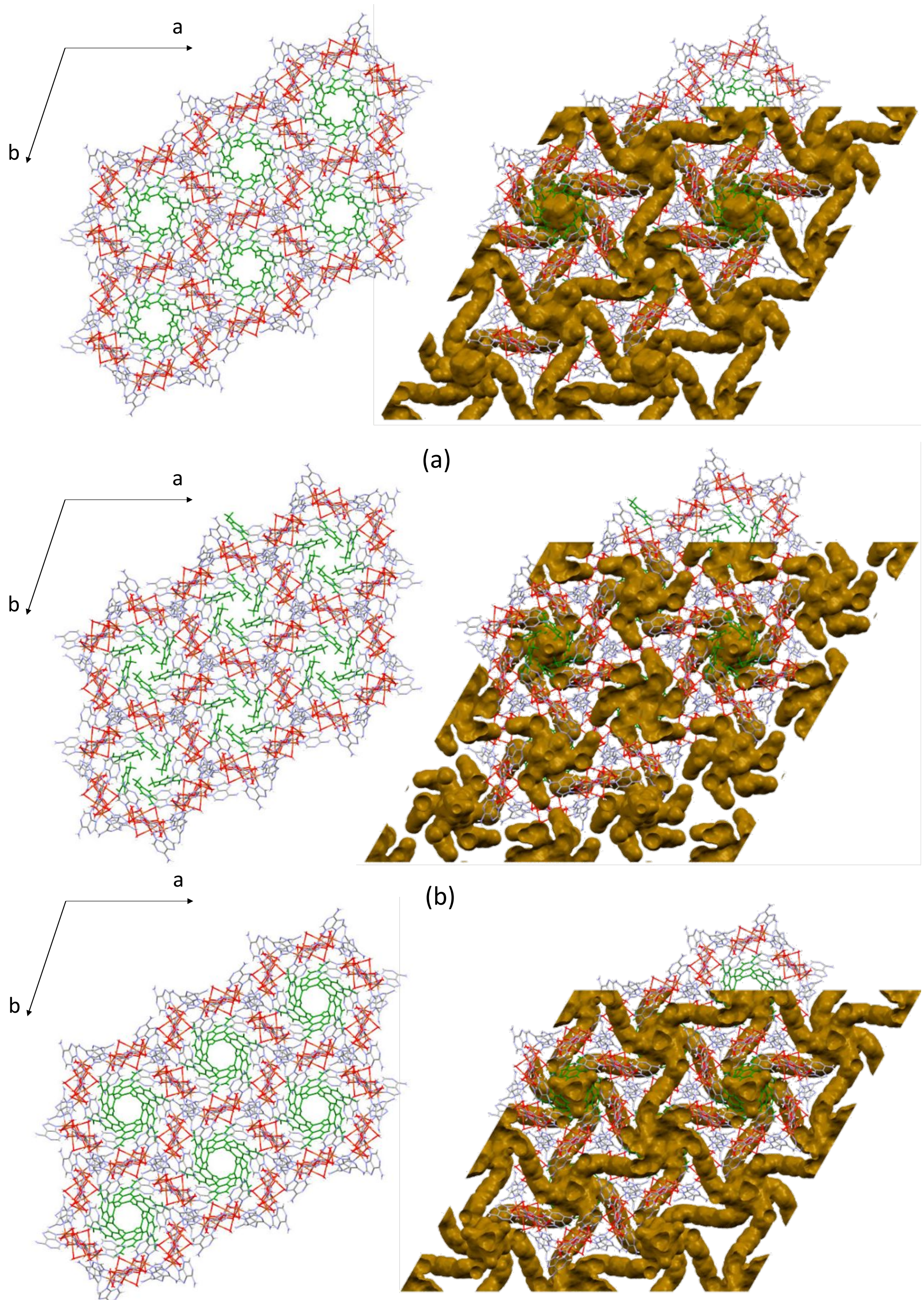

Figure S7. Channels for compounds (a) **$Cu_7$3MBz**, (b) **$Cu_7$4MBz** and (c) **$Cu_7$2Naph** with the respective anions in green along the *c* crystallographic axis. Right side representation of the channels contact surface in the porous structure in brown.

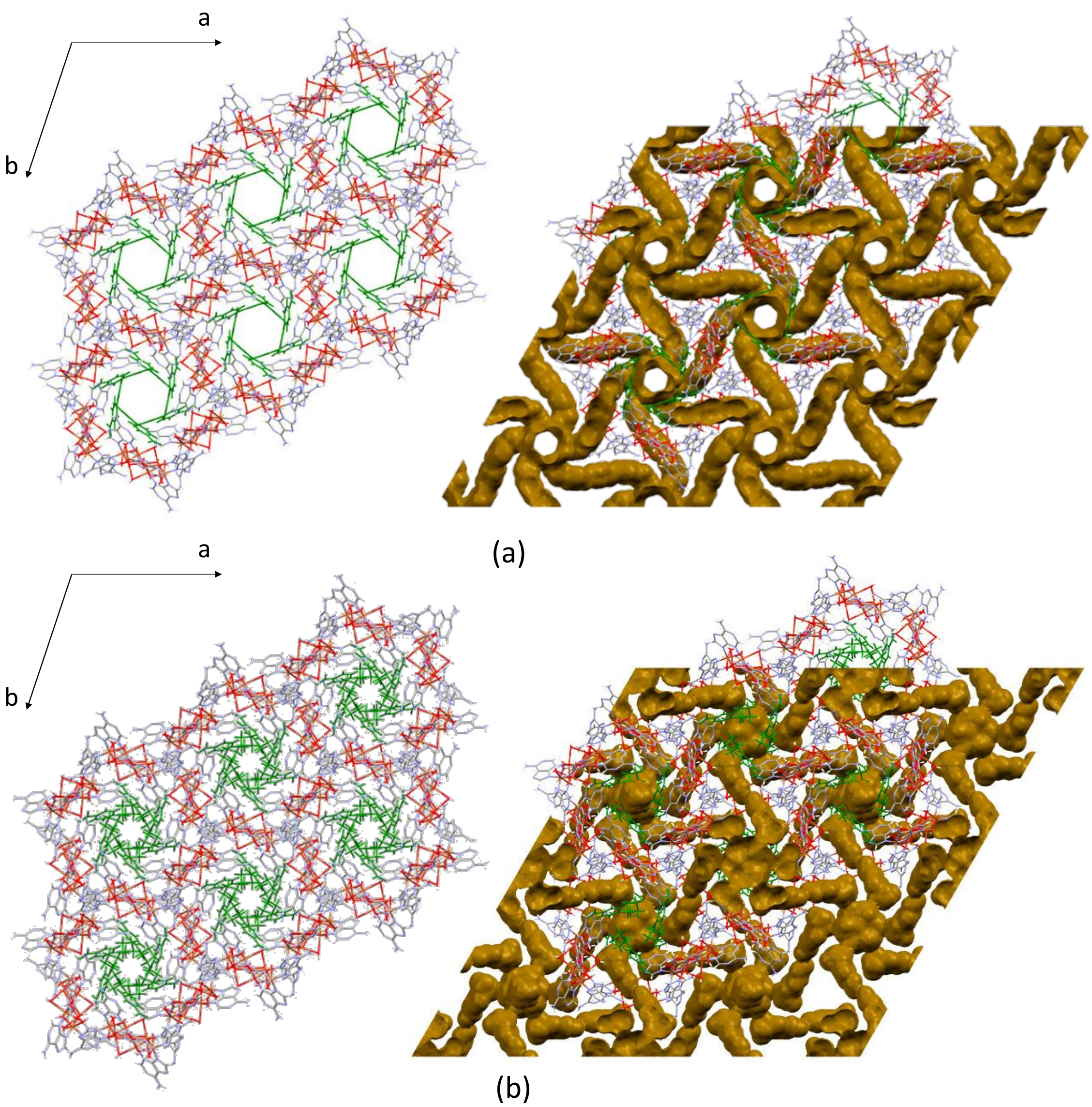

Figure S8. Channels for compounds (a) **$Cu_7$4Bi** and (b) **$Cu_7$4PBz** with the respective anions in green along the *c* crystallographic axis. Right side representation of the channels contact surface in the porous structure in brown.

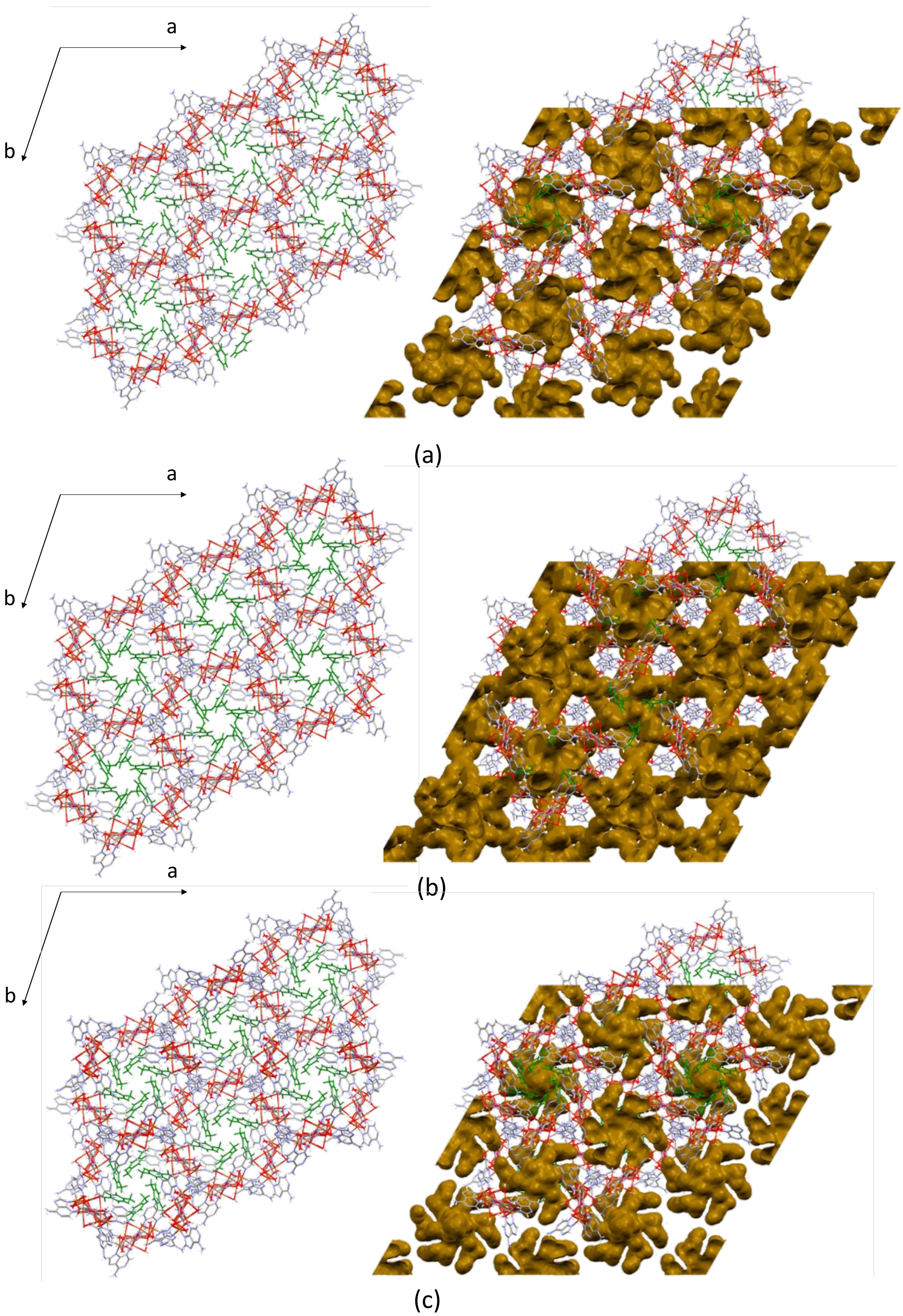

Figure S9. Channels for compounds (a) **Cu_7Bz_D**, (b) **$Cu_7$3MBz_D** and (c) **$Cu_7$4MBz_D** with the respective anions in green along the *c* crystallographic axis. Right side representation of the channels contact surface in the porous structure in brown.

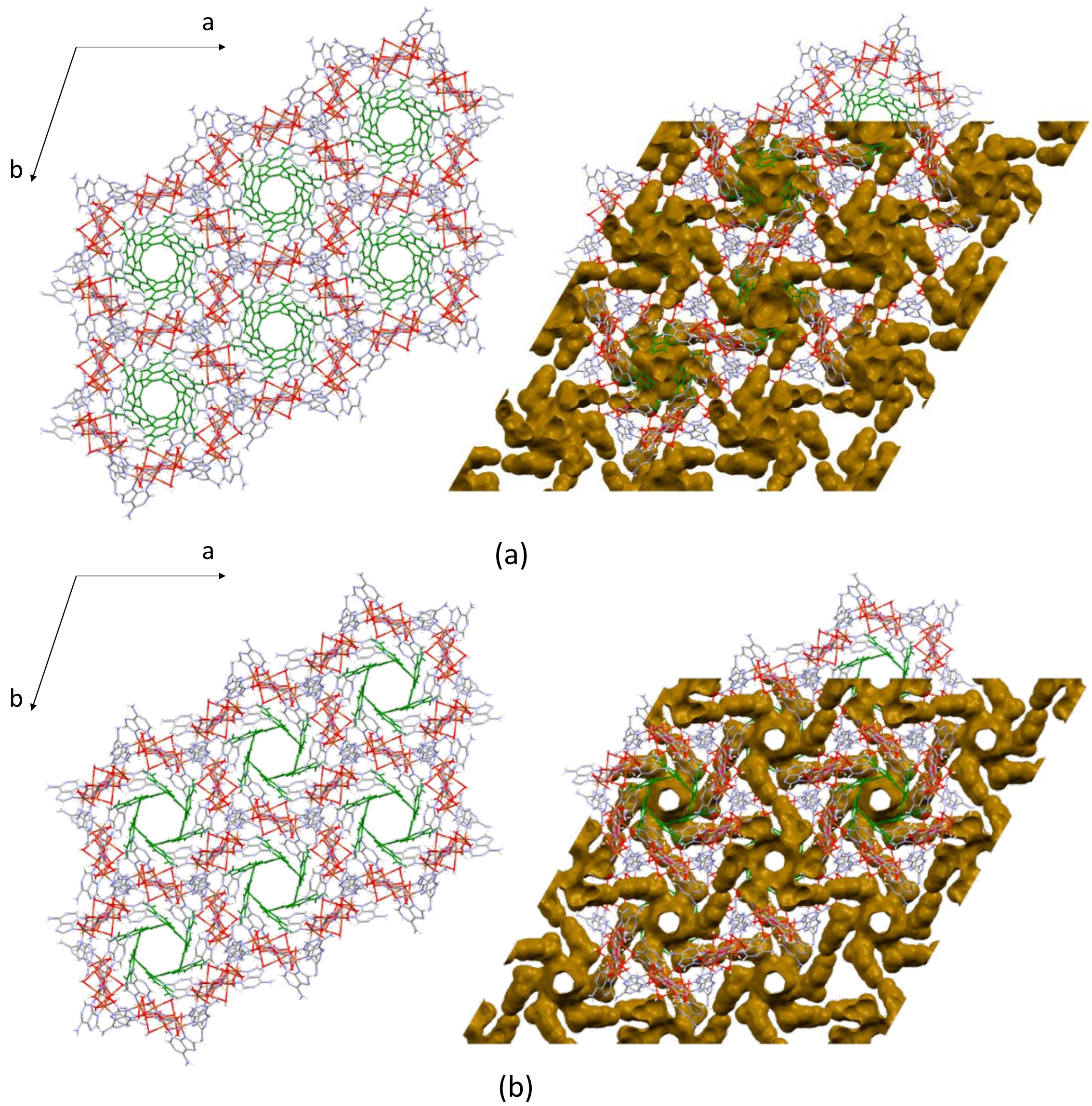

Figure S10. Channels for compounds (a) **Cu$_7$2Naph_D** and (b) **Cu$_7$4Bi_D** with the respective anions in green along the *c* crystallographic axis. Right side representation of the channels contact surface in the porous structure in brown.

S3. WATER ADSORPTION PROCESSES

Water vapor adsorption isotherms were obtained using a volumetric adsorption analyzer (3Flex, Micromeritics). Samples were outgassed under vacuum at 30 °C for 6 h prior to analysis. Measurements were performed at 293 K under vacuum using pure H_2O vapor. Equilibrium was defined as a pressure change below 0.01% over ten times the equilibration interval (40–60 s).

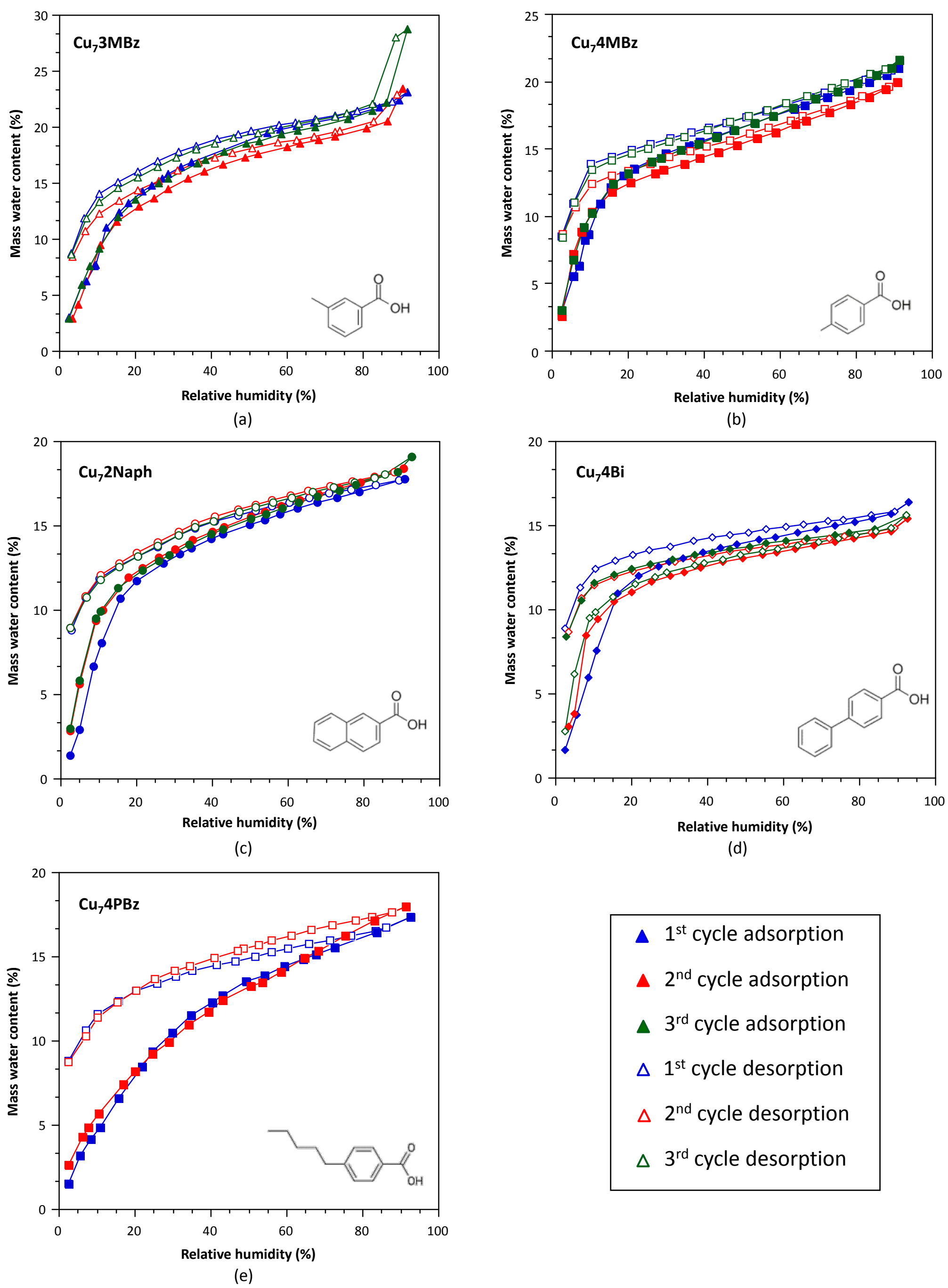

Figure S11. Consecutive adsorption/desorption cycling (1st cycle: blue symbols; 2nd cycle: red symbols and 3rd cycle: green symbols) at 20 °C for (a) **$Cu_7$3MBz**, (b) **$Cu_7$4MBz**, (c) **$Cu_7$2Naph**, (d) **$Cu_7$4Bi** and (e) **$Cu_7$4PBz**.

Table S9. Compilation of the water content and comparison with the adsorbed and water molecules determined by SC-XRD determination for all the compounds.

	molecular weight without H_2O_{cryst}	*molecular weight without H_2O_{cryst} and H_2O_{coord}*	*H_2O_{Crista}*	*%H_2O without H_2O_{coord}*	*%Ads H_2O exp.*	*Equivalent H_2O adsorbed*	*Difference with H_2O_{cryst} (+)*
**Cu_7Bz**	1701.9	1593.8	17	19.2	28.1	25	8
**Cu_7Bz_D**	1701.9	1593.8	13	14.7	28.1	25	12
**$Cu_7$3MBz**	1729.9	1621.9	12	13.3	23.1	21	9
**$Cu_7$3MBz_D**	1729.9	1621.9	11	12.2	23.1	21	10
**$Cu_7$4MBz**	1729.9	1621.9	11	12.2	21.1	19	8
**$Cu_7$4MBz_D**	1729.9	1621.9	10	11.1	21.1	19	9
**$Cu_7$2Naph**	1804.0	1695.9	12	12.7	17.8	17	5
**$Cu_7$2Naph_D**	1804.0	1695.9	11	11.7	17.8	17	6
**$Cu_7$4Bi**	1856.1	1748.0	12	12.4	16.4	17	5
**$Cu_7$4Bi_D**	1856.1	1748.0	9	9.3	16.4	17	8
**$Cu_7$4PBz**	1844.2	1736.1	8	8.3	17.3	16	8

S4. DRUGS LOADED PROCEDURE AND DATA

The procedure used to capture the different analytes in the isoreticular compounds is detailed below. In order to execute this procedure, stock solutions with a concentration of 25 mg/mL of the analytes employed (DMF, DMSO, RESOR, TMU, BnOH) were meticulously prepared. To this purpose, 6.250 g of analyte was precisely weighed and dissolved in water using a 250 mL volumetric flask. From these stock solutions, 5 mL aliquots were extracted and deposited in a vial. From this, 2 mL were transferred using a micropipette to a new vial, where 50 mg of **Cu_7Bz** derivate SMOF was added. The mixture was then placed in a thermostatic bath with agitation at 30 °C for 24 h. The residual 3 mL were employed to verify the initial concentration of the solution by HPLC, Figure S12.

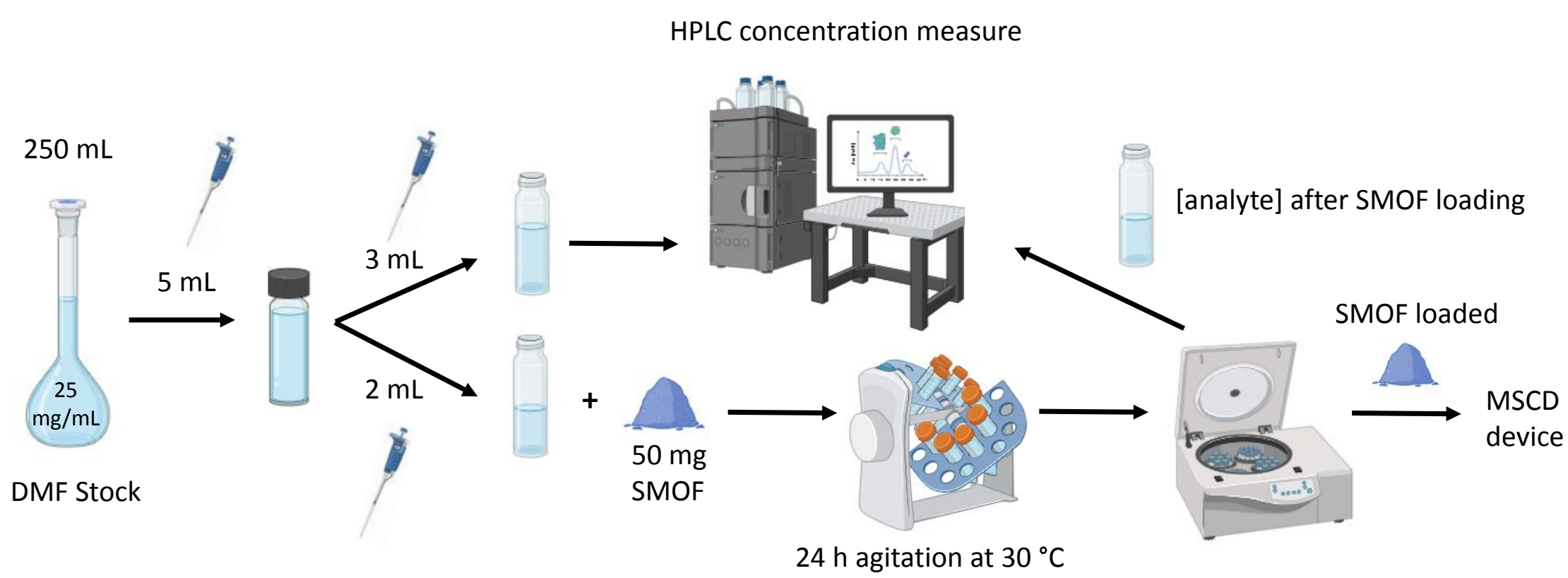

Figure S12. Schematic steps of the SMOFs drugs loading for DMF analyte and concentration measuring procedure for **Cu_7Bz**, **$Cu_7$3MBz**, **$Cu_7$4MBz**, **$Cu_7$2Naph** and **$Cu_7$4Bi**.

After 24 h of contact between the SMOF and the corresponding analyte, the vial is centrifuge to separate the SMOF particles from the final solution. It is needed to say that both, the initial stock solution (the remaining 3 mL) and the final solution, concentration are measured by HPLC. Meanwhile, the SMOF particles that have been recovered after the incubation process with the analytes are used to quantify the drug loaded by the magnetic suspension equipment determining their critical distance.

The reproducibility of the loading of the three drugs (5-fluorouracil, gabapentin and cycloserine) has been checked by repeating the sorption experiment three times, the associated standard deviation comes from the differences of the sorption values of this three experiments (Figure S13).

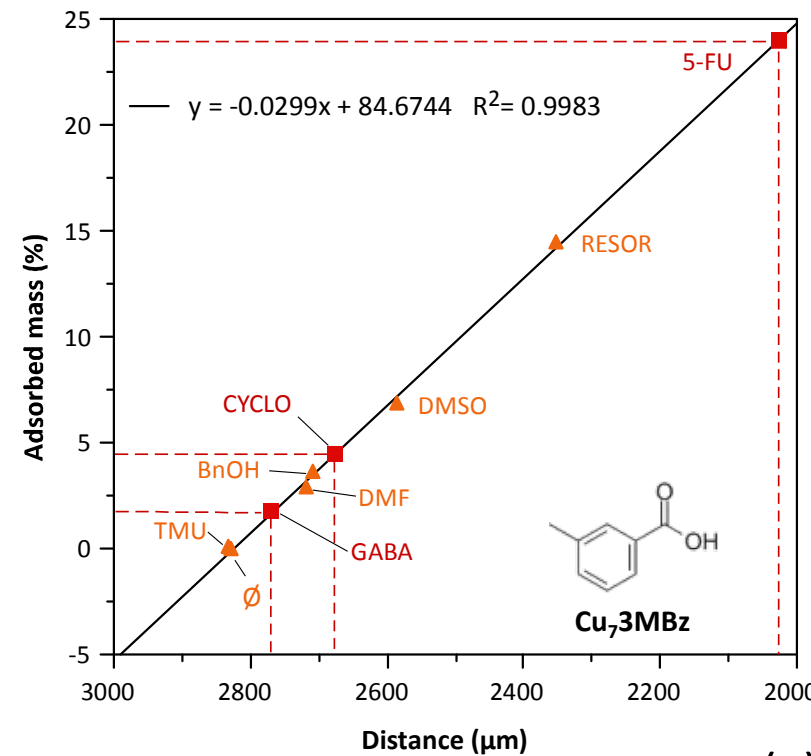

(a)

Calibration curve (HPLC)	D (µm)	Adsorbed mass (%)	Adsorbed molecules/ SMOF formula
Ø	2828	-	-
BnOH	2710	3.7	0.6
DMF	2720	2.9	0.7
DMSO	2586	6.9	1.5
RESOR	2352	14.4	2.3
TMU	2832	0.1	0.0
**Extrapolated values**	**D (µm)**	**Adsorbed mass (%)**	**Adsorbed molecules/ SMOF formula**
CYCLO	2678	4.5	0.8
5-FU	2027	24.0	3.2
GABA	2770	1.7	0.2

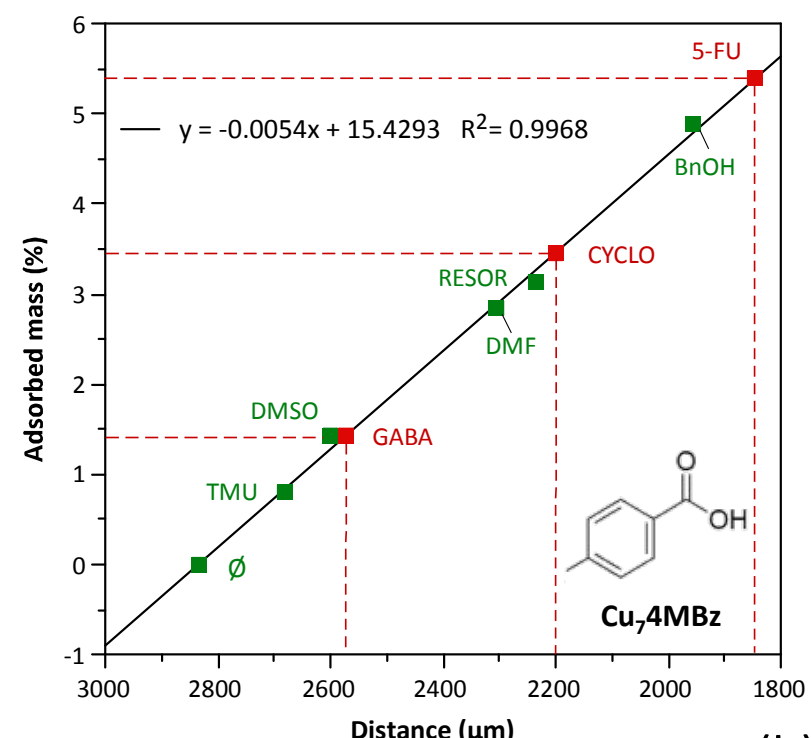

(b)

Calibration curve (HPLC)	D (µm)	Adsorbed mass (%)	Adsorbed molecules/ SMOF formula
Ø	2832	-	-
BnOH	1954	4.9	0.8
DMF	2306	2.8	0.7
DMSO	2600	1.4	0.3
RESOR	2234	3.1	0.5
TMU	2682	0.8	0.1
**Extrapolated values**	**D (µm)**	**Adsorbed mass (%)**	**Adsorbed molecules/ SMOF formula**
CYCLO	2202	3.5	0.6
5-FU	1845	5.4	0.7
GABA	2572	1.4	0.1

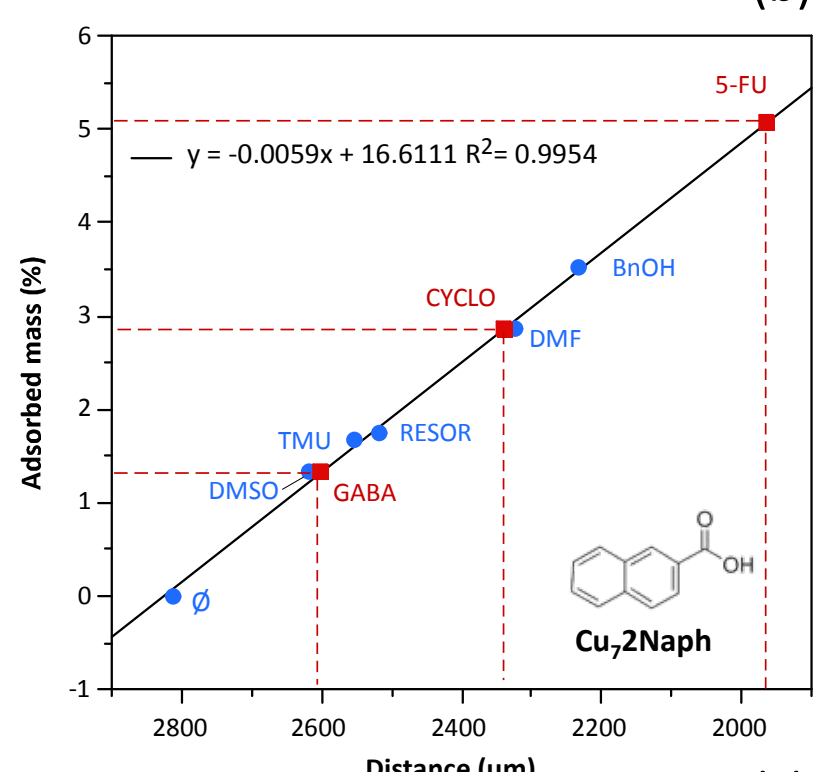

(c)

Calibration curve (HPLC)	D (µm)	Adsorbed mass (%)	Adsorbed molecules/ SMOF formula
Ø	2814	-	-
BnOH	2232	3.5	0.6
DMF	2324	2.9	0.7
DMSO	2618	1.3	0.3
RESOR	2518	1.8	0.3
TMU	2555	1.7	0.3
**Extrapolated values**	**D (µm)**	**Adsorbed mass (%)**	**Adsorbed molecules/ SMOF formula**
CYCLO	2342	2.9	0.5
5-FU	1966	5.1	0.7
GABA	2604	1.3	0.1

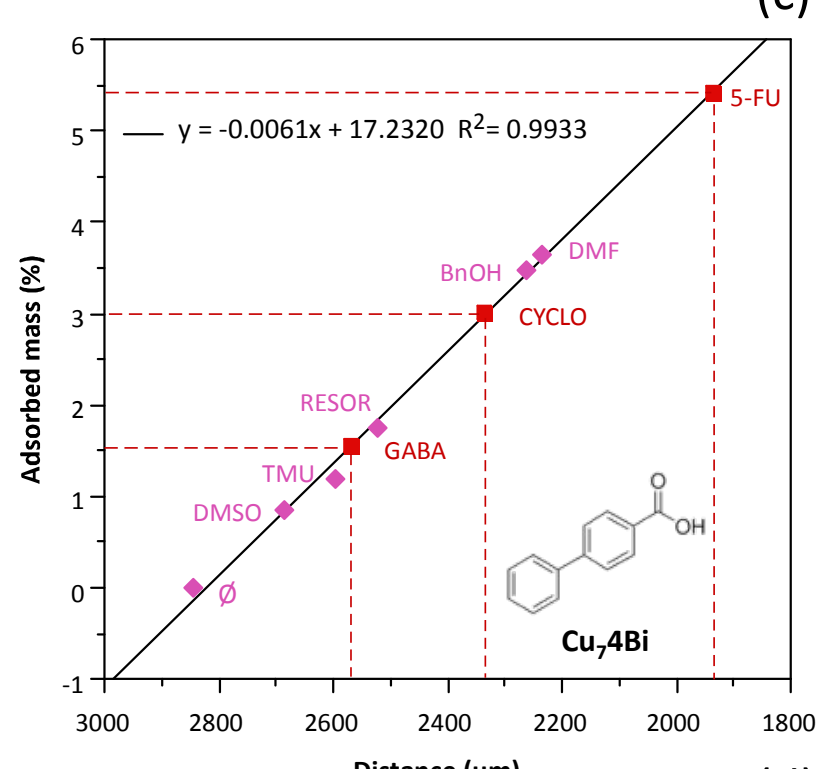

(d)

Calibration curve (HPLC)	D (µm)	Adsorbed mass (%)	Adsorbed molecules/ SMOF formula
Ø	2844	-	-
BnOH	2263	3.5	0.6
DMF	2234	3.6	0.9
DMSO	2687	0.9	0.2
RESOR	2524	1.8	0.3
TMU	2595	1.2	0.2
**Extrapolated values**	**D (µm)**	**Adsorbed mass (%)**	**Adsorbed molecules/ SMOF formula**
CYCLO	2332	3.0	0.5
5-FU	1937	5.4	0.8
GABA	2570	1.6	0.2

Figure S13. Linear dependence of adsorbed mass (%) *vs* critical distance (µm). Calibration curves for **$Cu_7$3MBz** (b) **$Cu_7$4MBz** (c) **$Cu_7$2Naph** and (d) **$Cu_7$4Bi** obtained by HPLC for the calibrate molecules (purple circle), altogether with the extrapolated values for the studied drug molecules (red square). Ø represents the pristine sample of each compound.

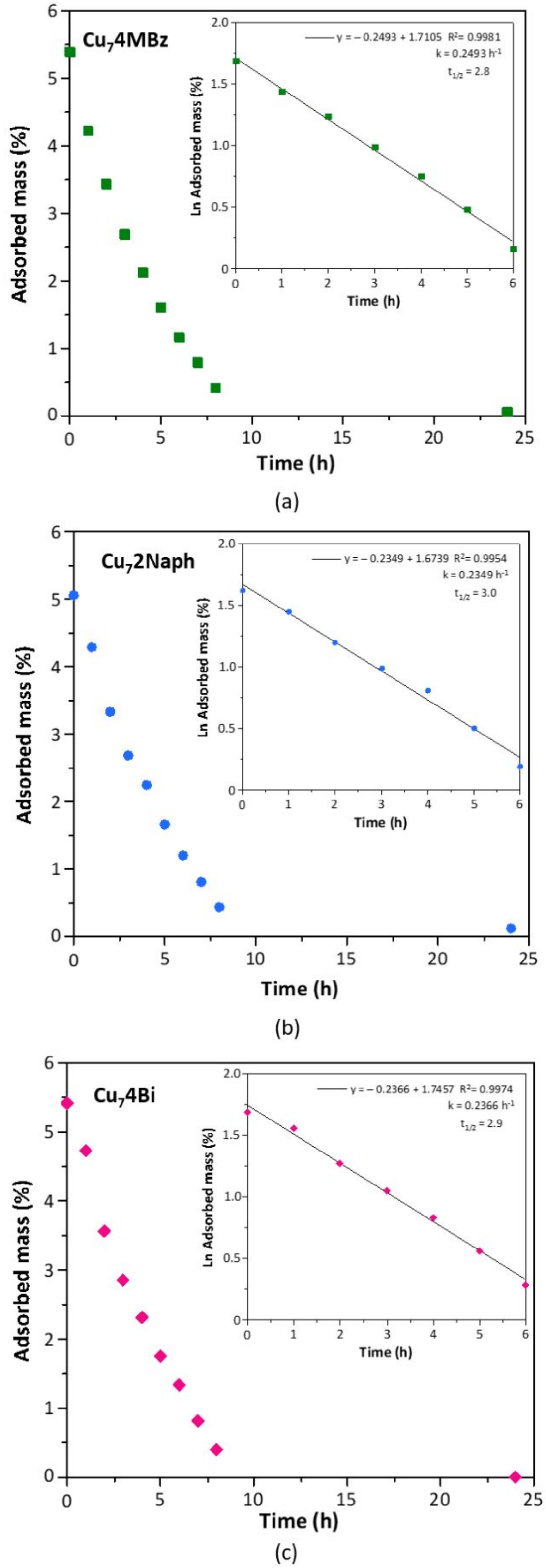

Figure S14. Desorption isotherms in water solution of the 5-FU in (a) **$Cu_7$4MBz** (b) **$Cu_7$2Naph** and (c) **$Cu_7$4Bi** compounds. Inset: fitting to a first order kinetic model considering the first six hours of the desorption process.

S5. ADSORPTIVE VOLUMEN AND SHAPE

The following Tables S10 and S11 and Figure S14 and S15 shows the size and shape data of the analytes used for calibration and of the drug molecules studied in this work. To take into account with the results below described, the dimensions of the void (Å) of each structure are: **Cu_7Bz**: 4.00−6.53, **$Cu_7$3MBz**: 2.41−6.43, **$Cu_7$4MBz**: 1.84−6.00, **$Cu_7$2Naph**: 2.68−6.78, **$Cu_7$4Bi**: 3.28−6.19 and **$Cu_7$4PBz**: 1.89−5.34.

Table S10. Physicochemical parameters of the analytes used in calibration ordered by increasing hydrophobicity.

Calibration analytes	DMSO	DMF	TMU	Resorcinol	Benzyl alcohol
**Structure**	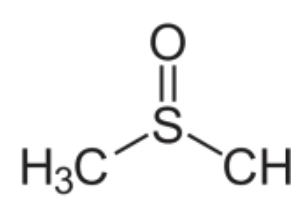	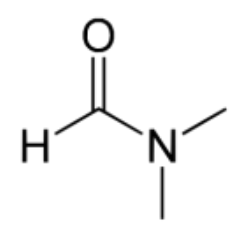	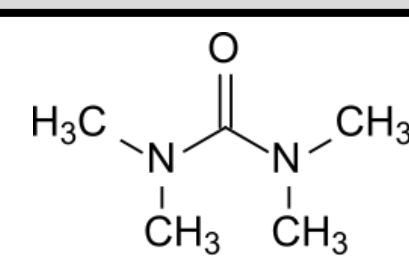	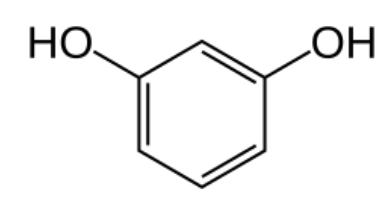	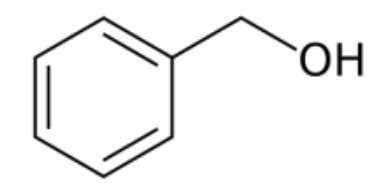
**P_M (g/mol)**	78.13	73.11	116.16	110.11	108.14
**Partition coefficient (Log P)**	-1.35	-0.85	0.19	0.80	1.10
**Polar surface (Å^2)**	17.07	20.31	23.55	40.46	20.23
**Van der Waals Volume (Å^3)**	72.67	76.80	123.57	99.01	107.43
**Size (Å)**	6.20–7.12	6.26–7.12	6.98–8.84	7.27–7.92	6.72–9.20
**Captured mass (%)**					
**Cu_7Bz**	9.6	2.2	2.1	16.9	4.5
**$Cu_7$3MBz**	6.9	2.9	0.1	14.4	3.7
**$Cu_7$4MBz**	1.4	2.8	0.8	3.1	4.9
**$Cu_7$2Naph**	1.3	2.9	1.7	1.8	3.5
**$Cu_7$4Bi**	0.9	3.6	1.2	1.8	3.5
**$Cu_7$4PBz**	-	-	-	-	-

Table S11. Physicochemical parameters of the drugs employed ordered by increasing hydrophobicity.

**Calibration analytes**	**Cycloserine**	**Gabapentin**	**5-Fluorouracil**
**Structure**	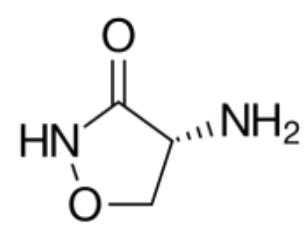	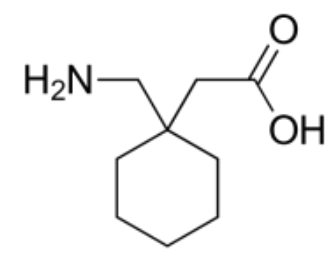	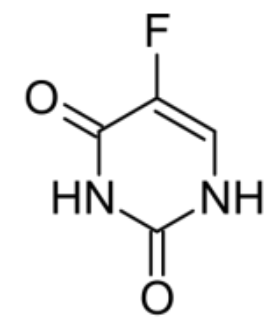
**P_M (g/mol)**	102.09	171.24	130.08
**Partition coefficient (Log P)**	-2.89	-1.25	-0.85
**Polar surface (Å^2)**	64.35	63.32	58.53
**Van der Waals Volume (Å^3)**	85.12	176.16	94.03
**Size (Å)**	6.92 – 7.42	8.28 – 9.98	7.02 – 7.98
**Captured mass (%)**			
**Cu_7Bz**	7.6	2.8	31.4
**Cu_73MBz**	4.5	1.7	24.0
**Cu_74MBz**	3.5	1.4	5.4
**Cu_72Naph**	2.9	1.3	5.1
**Cu_74Bi**	3.0	1.6	5.4
**Cu_74PBz**	-	-	-

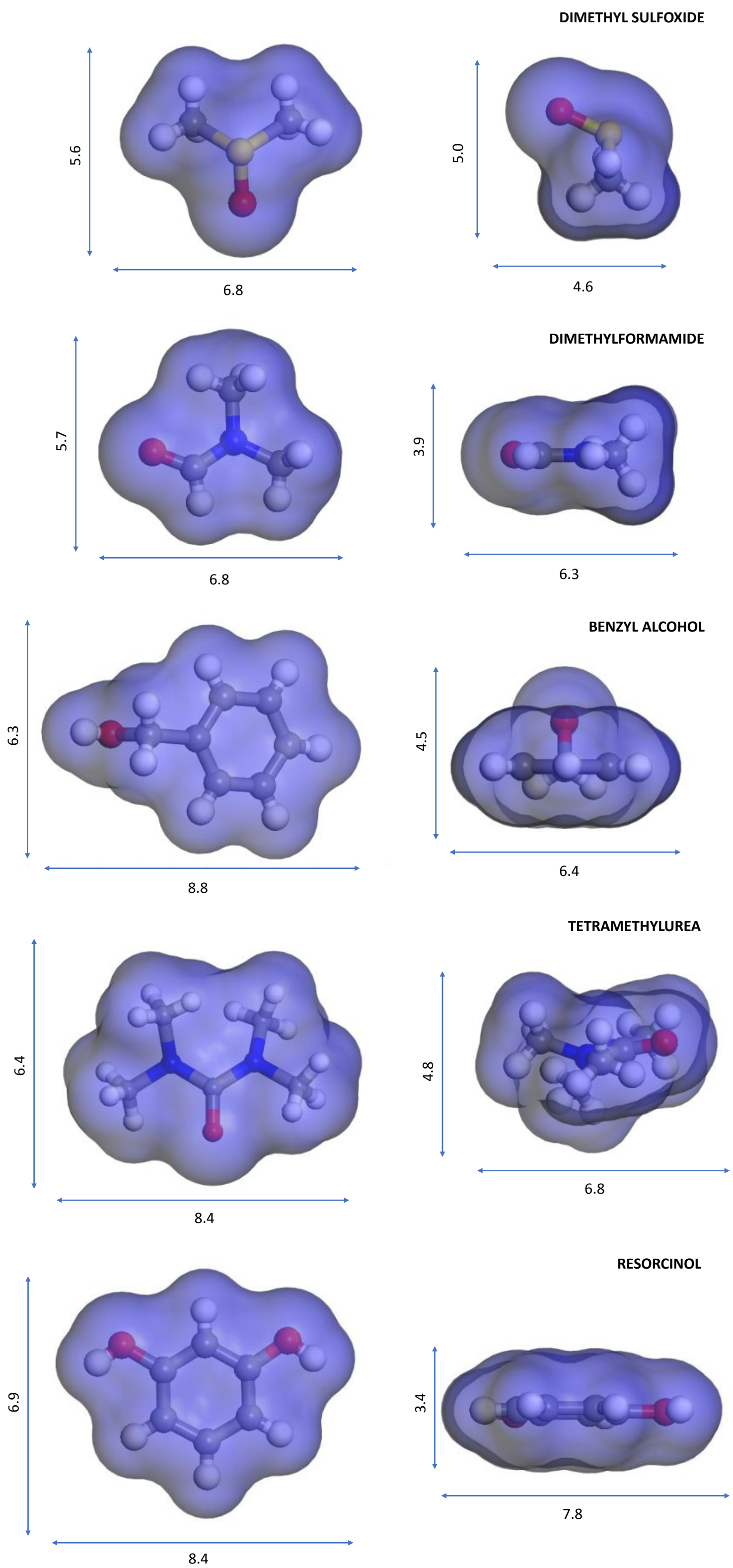

Figure S15. Shape and dimensions (Å) of the studied molecules used in the calibration. The same scale has been used for both images to facilitate a direct visual comparison between them. A 1.0 Å probe has been employed to define the Connolly surface of the adsorptive molecules (Materials Studio, *BIOVIA Materials Studio*: 2017 R2, 17.2.0.1626, package).

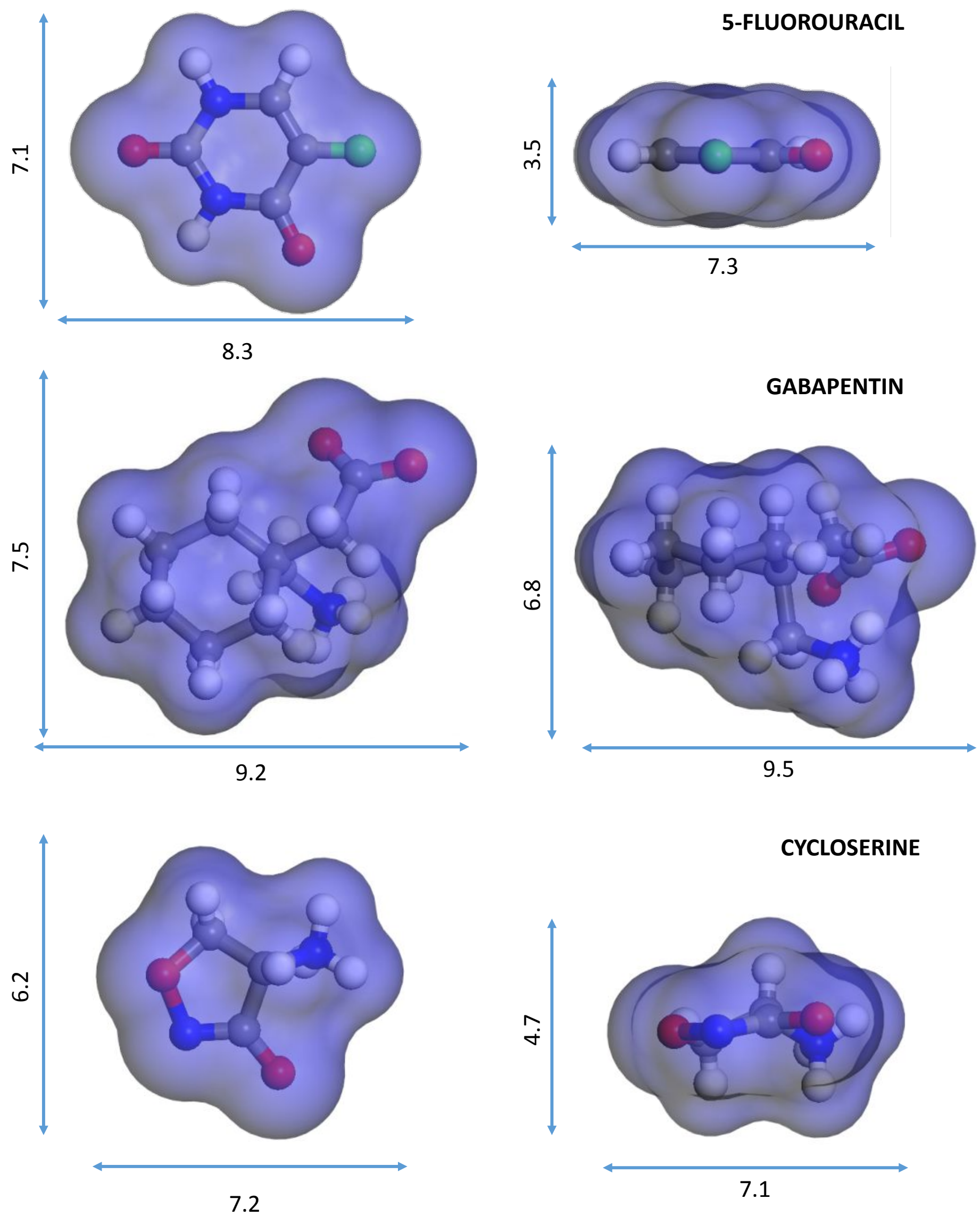

Figure S16. Shape and dimensions (Å) of the studied adsorptive drugs of this work. The same scale has been used for both images to facilitate a direct visual comparison between them. A 1.0 Å probe has been employed to define the Connolly surface of the adsorptive molecules (Materials Studio, *BIOVIA Materials Studio*: 2017 R2, 17.2.0.1626, package).

S6. CYTOTOXICITY

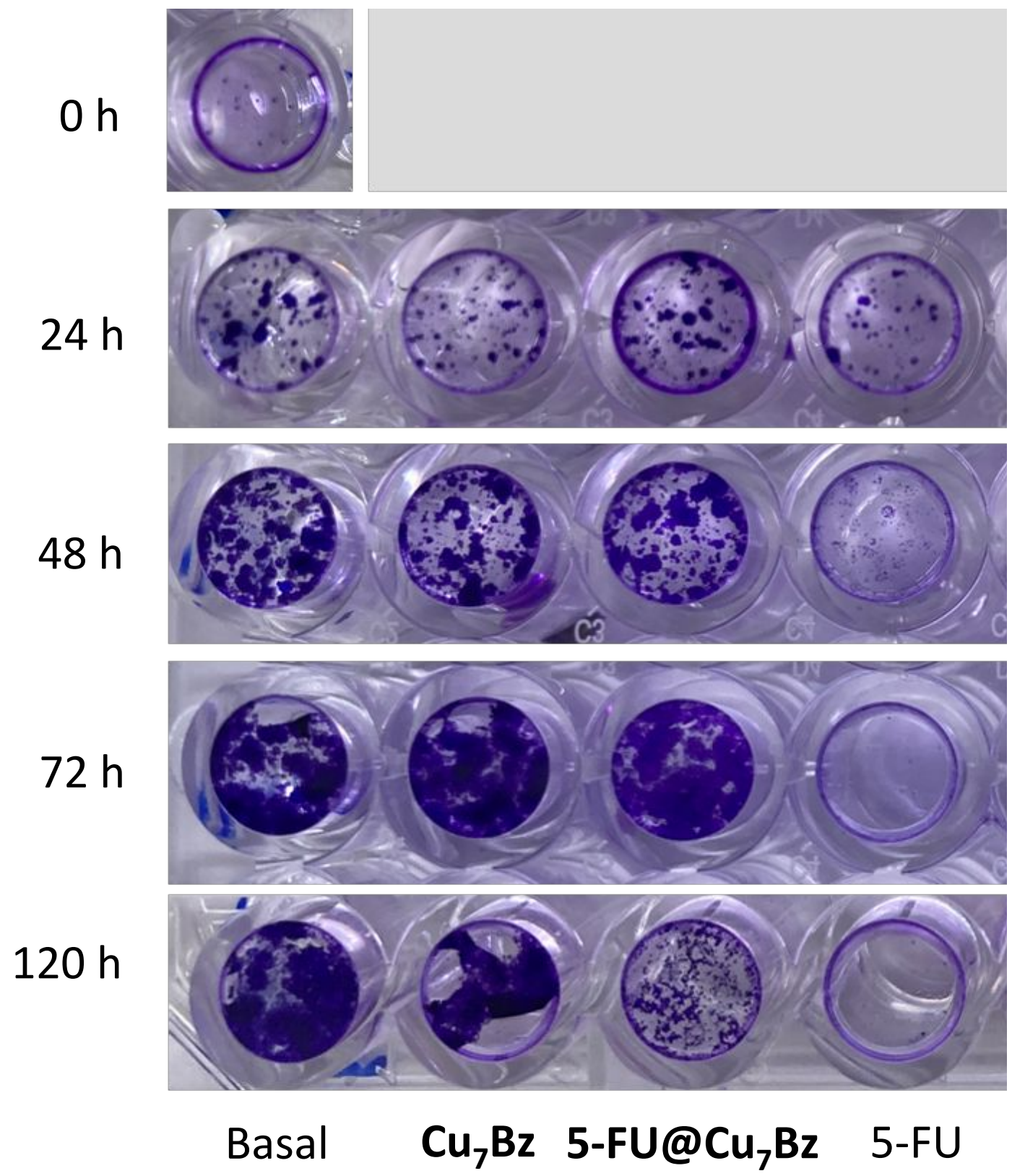

Figure S17. Crystal violet staining of *HCT116* cells grown for 24 h, 48 h, 72 h and 120 h in basal conditions and in the presence of the different compounds. Image is representative of the four independent assays.

S7. POWDER X-RAY DIFFRACTION

Powder X-ray diffraction patterns were collected for all **Cu_7Bz** derivate samples (**$Cu_7$3MBz**, **$Cu_7$4MBz**, **$Cu_7$2Naph**, **$Cu_7$4Bi** and **$Cu_7$4PBz**) to verify the purity of all synthesis.

The Figures S18 and S19 below shows that the theoretical PXRD obtained from the SC structure is identical to the ones obtained from the experimental measurements (the one obtained after introducing the crystals in the Lindemann with their mother liquid and the regular one after vacuum filtering). In the case of the Benzoic acid, 3-methylbenzoic acid, 4-methylbenzoic acid, 2-naphthalecarboxylic acid and 4-biphenylcarboxylic acid we have been able to measure a filtered sample so we can compare the filtered sample with the simulated pattern of the single-crystal structure solved for this dried sample with the codes **Cu_7Bz_D**, **$Cu_7$3MBz_D**, **$Cu_7$4MBz_D**, **$Cu_7$2Naph_D** and **$Cu_7$4Bi_D**.

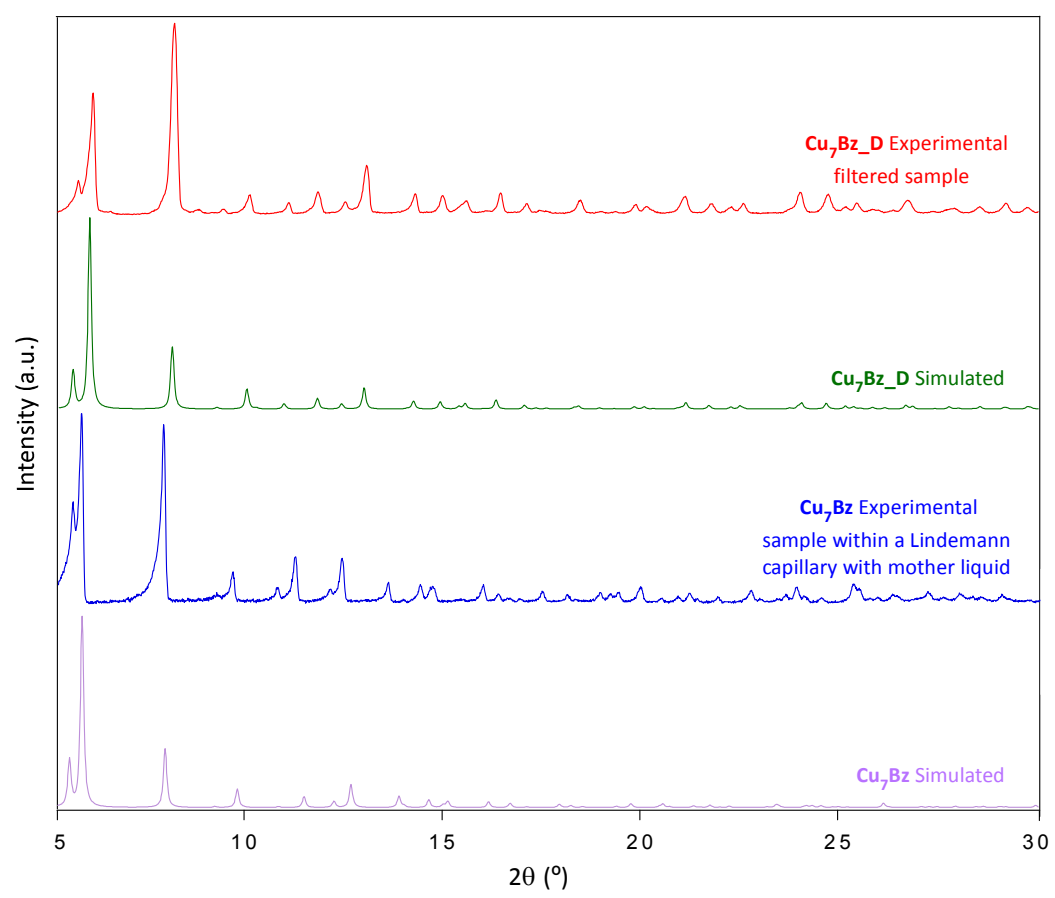

(a)

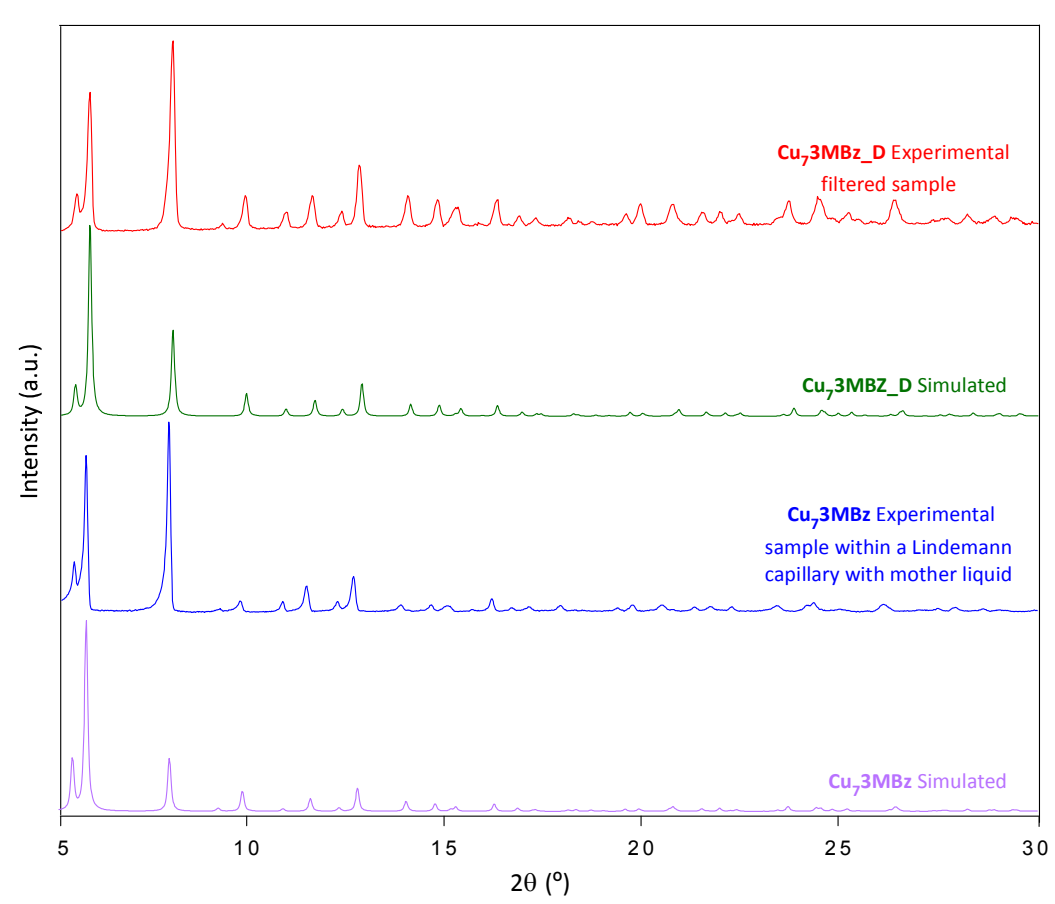

(b)

Figure S18. Diffractograms of (a) **Cu_7Bz** and (b) **$Cu_7$3MBz** compounds respectively, comparing the simulated from the crystal structure and as obtained after vacuum filtration and in the Lindemann capillary with the mother liquid.

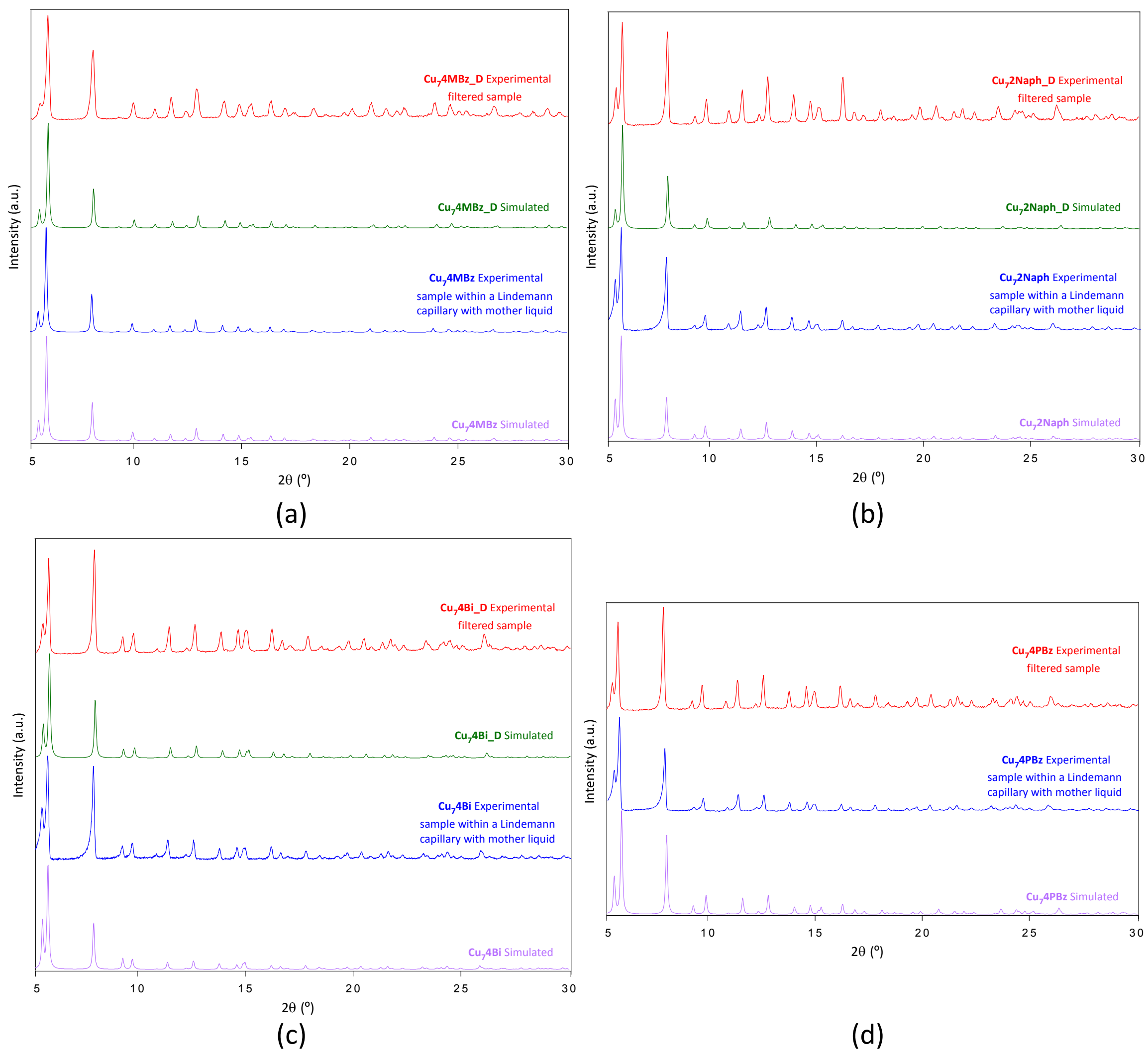

Figure S19. Diffractograms of (a) **$Cu_7$4MBz**, (b) **$Cu_7$2Naph**, (c) **$Cu_7$4Bi** and (d) **$Cu_7$4PBz** compounds respectively, comparing the simulated from the crystal structure and as obtained after vacuum filtration and in the Lindemann capillary with the mother liquid.

S8. INFRARED SPECTRA (FTIR) OF THE BENZOATE DERIVATE COMPOUNDS

All FTIR spectra in Figure S20 exhibit the bands corresponding to the stretching vibration of the C–H and N–H bonds of the purine bases between 3400 and 3100 cm^{-1}, together with the O–H bonds of the water molecules. The bands around 1610 cm^{-1} belong to the nucleobase ligands for the imino groups. The antisymmetric tension of the carboxylate group of anions are observed around 1640 cm^{-1}. The presence of the peak located around 1600 cm^{-1} corresponding to the vibration of the C=C bond and the deformation of the NH_2 group allows the identification of adeninato ligands. The bands corresponding for methyl groups are located around 980 cm^{-1}. The vibrational bands of the M–N bonds appear below 550 cm^{-1}.

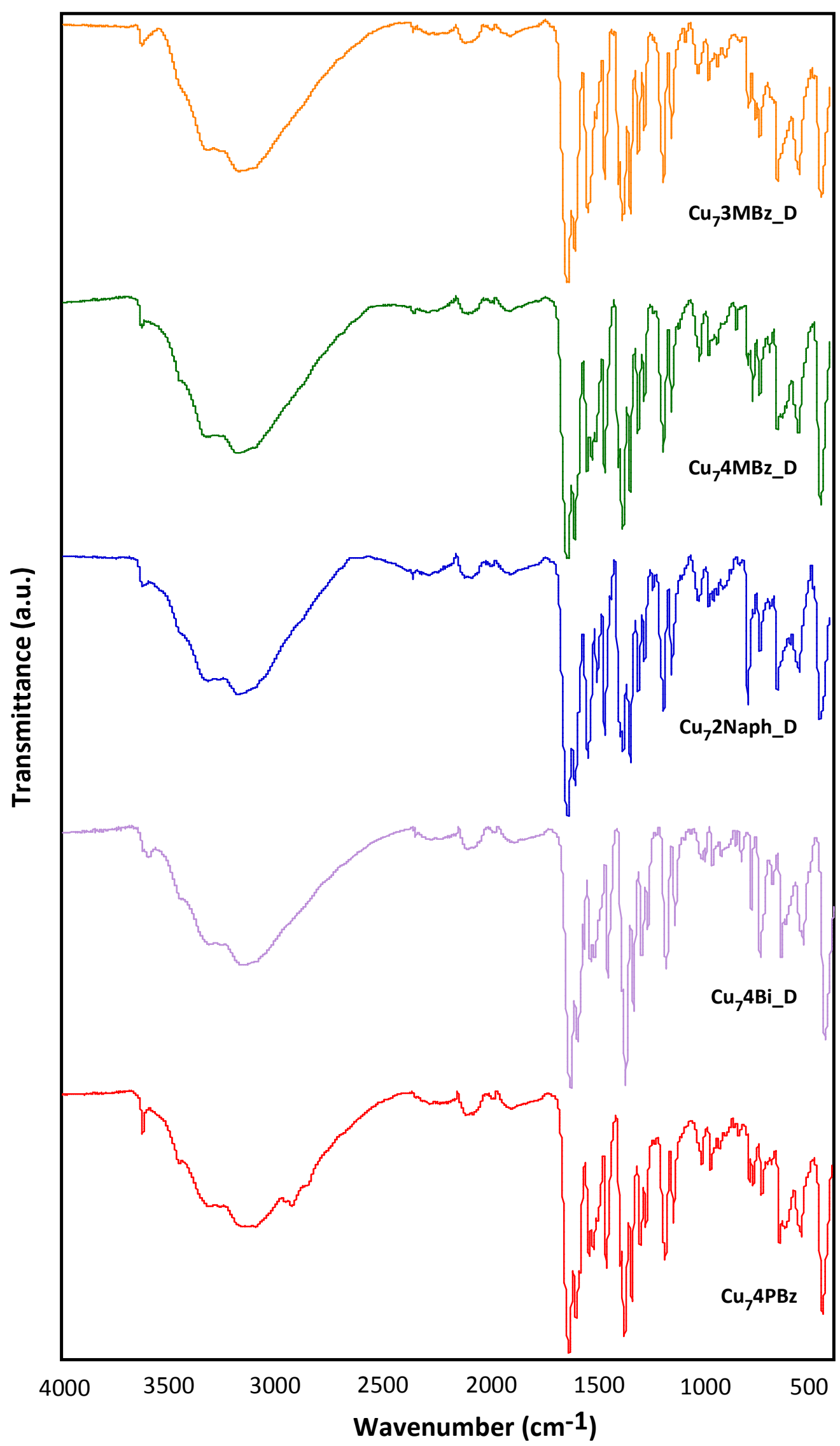

Figure S20. FTIR spectra of the **$Cu_7$3MBz_D** (orange), **$Cu_7$4MBz_D** (green), **$Cu_7$2Naph_D** (blue), **$Cu_7$4Bi_D** (pink), and **$Cu_7$4PBz** (red) compounds.

S9. EXPERIMENTAL MEASUREMENTS OF DRUG LOADED SMOFs

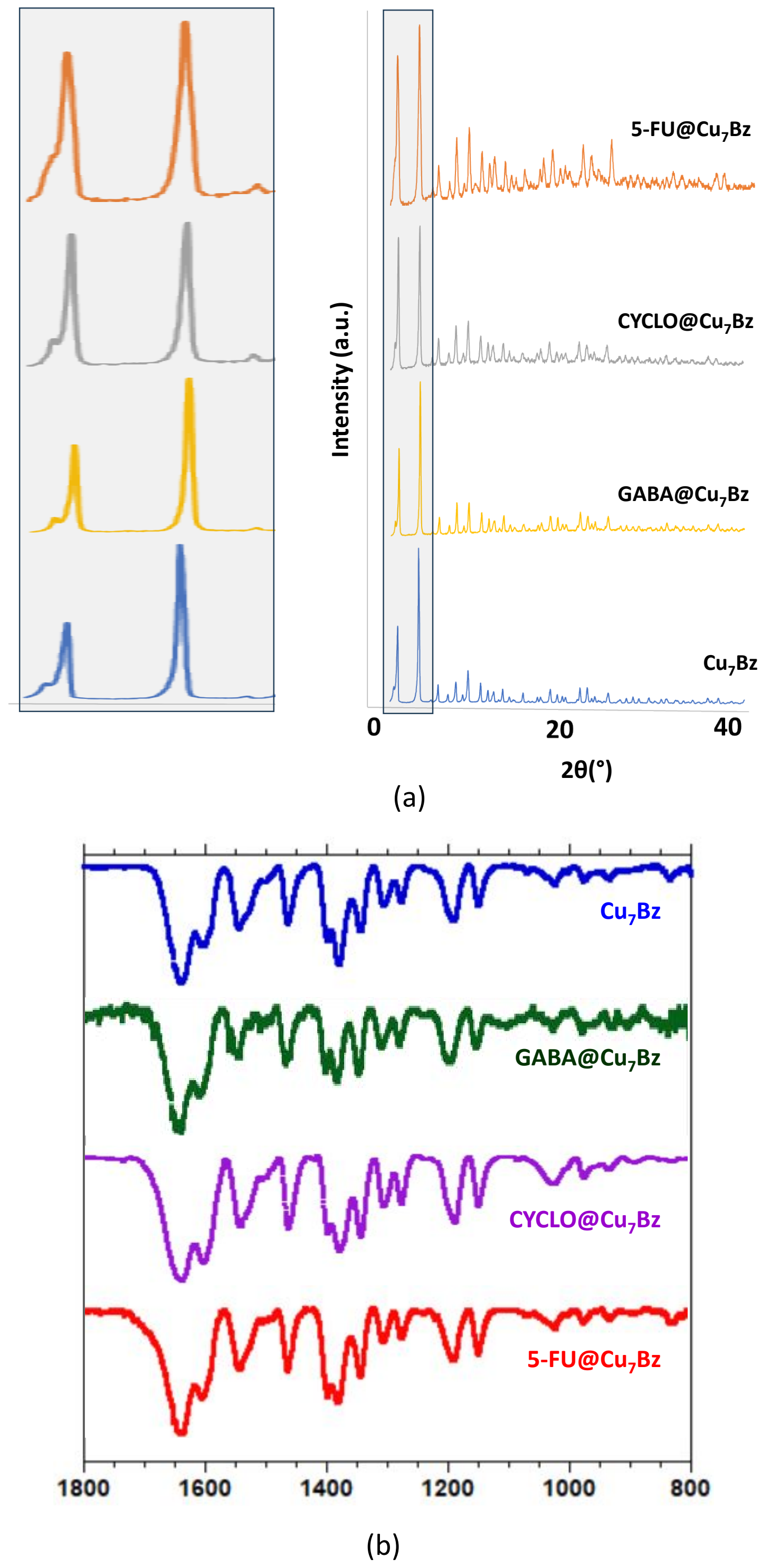

Figure S21.- FTIR spectra and PXRD diffractograms of the **Cu_7Bz** compound after drug loaded process.
